# Crystal Structures of Three Classes of Non-Steroidal Anti-Inflammatory Drugs in Complex with Aldo-Keto Reductase 1C3

**DOI:** 10.1371/journal.pone.0043965

**Published:** 2012-08-28

**Authors:** Jack U. Flanagan, Yuliana Yosaatmadja, Rebecca M. Teague, Matilda Z. L. Chai, Andrew P. Turnbull, Christopher J. Squire

**Affiliations:** 1 Auckland Cancer Society Research Centre, University of Auckland, Auckland, New Zealand; 2 Maurice Wilkins Centre for Molecular Biodiscovery, University of Auckland, Auckland, New Zealand; 3 School of Biological Sciences, University of Auckland, Auckland, New Zealand; 4 Cancer Research Technology Discovery Laboratories, Wolfson Institute for Biomedical Research, London, United Kingdom; National Institute for Medical Research, Medical Research Council, London, United Kingdom

## Abstract

Aldo-keto reductase 1C3 (AKR1C3) catalyses the NADPH dependent reduction of carbonyl groups in a number of important steroid and prostanoid molecules. The enzyme is also over-expressed in prostate and breast cancer and its expression is correlated with the aggressiveness of the disease. The steroid products of AKR1C3 catalysis are important in proliferative signalling of hormone-responsive cells, while the prostanoid products promote prostaglandin-dependent proliferative pathways. In these ways, AKR1C3 contributes to tumour development and maintenance, and suggest that inhibition of AKR1C3 activity is an attractive target for the development of new anti-cancer therapies. Non-steroidal anti-inflammatory drugs (NSAIDs) are one well-known class of compounds that inhibits AKR1C3, yet crystal structures have only been determined for this enzyme with flufenamic acid, indomethacin, and closely related analogues bound. While the flufenamic acid and indomethacin structures have been used to design novel inhibitors, they provide only limited coverage of the NSAIDs that inhibit AKR1C3 and that may be used for the development of new AKR1C3 targeted drugs. To understand how other NSAIDs bind to AKR1C3, we have determined ten crystal structures of AKR1C3 complexes that cover three different classes of NSAID, *N*-phenylanthranilic acids (meclofenamic acid, mefenamic acid), arylpropionic acids (flurbiprofen, ibuprofen, naproxen), and indomethacin analogues (indomethacin, sulindac, zomepirac). The *N*-phenylanthranilic and arylpropionic acids bind to common sites including the enzyme catalytic centre and a constitutive active site pocket, with the arylpropionic acids probing the constitutive pocket more effectively. By contrast, indomethacin and the indomethacin analogues sulindac and zomepirac, display three distinctly different binding modes that explain their relative inhibition of the AKR1C family members. This new data from ten crystal structures greatly broadens the base of structures available for future structure-guided drug discovery efforts.

## Introduction

**Figure 1:**
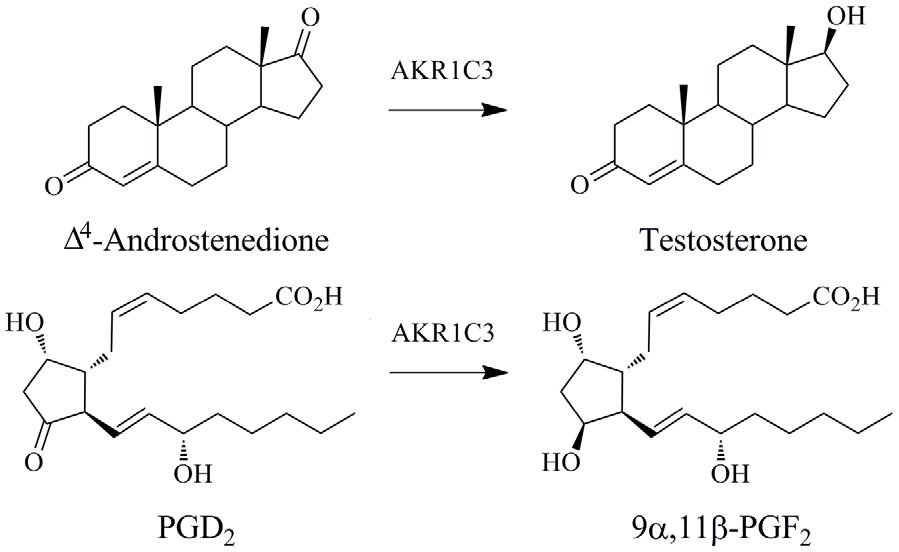
Two examples of reactions catalysed by AKR1C3. The 17-carbonyl of Δ4-androstenedione is reduced to a hydroxyl forming testosterone. The 9-carbonyl of prostaglandin D_2_ is reduced to an hydroxyl group to produce 9α,11β-PGF_2_. Figure drawn using ChemBioDraw Ultra 12.0 (CambridgeSoft).

Aldo-keto reductase 1C3 (AKR1C3; also known as prostaglandin F synthase, type 5 17β-hydroxysteroid dehydrogenase, type 2 3α-hydroxysteroid dehydrogenase, and dihydrodiol dehydrogenase X) is a human enzyme that catalyses the reduction of carbonyl groups on both steroids and prostaglandins (Figure 1). It converts 4-androstene-3,17-dione to testosterone, estrone to 17β-estradiol, and progesterone to 20α-hydroxyprogesterone, changing receptor affinities; testosterone has increased androgen receptor affinity, 17β-estradiol has increased estrogen receptor affinity, and 20α-hydroxyprogesterone has reduced affinity for progesterone receptors [1]–[3]. These products are of importance in the proliferative signalling of hormone responsive cells. The prostaglandin substrates PGH_2_ and PGD_2_ are structurally unrelated to the steroid hormones and are reduced to products PGF_2α_ and 9α,11β-PGF_2_ respectively [4]–[6]. These products display increased F prostanoid receptor affinity, and enhanced proliferative activity. In the absence of AKR1C3, PGD_2_ spontaneously dehydrates and isomerizes to form PGJ_2_ products which are anti-inflammatory, promote differentiation, and display anti-neoplastic effect [2], [7]–[12]. The role of AKR1C3 or its products in hormone-dependent and prostaglandin-dependent cancers has been investigated in a number of systems including breast cancer, prostate cancer, endometrial cancer, and acute myelogenous leukemia [2], [13]–[17]. AKR1C3 is over-expressed in a variety of cancers, most notably prostate and breast cancer, with a correlation between expression levels and the aggressiveness of the disease [18]–[23]. AKR1C3-dependent hormone and prostaglandin metabolism is therefore an attractive target for drug development with application to both hormone-dependent and independent cancers. Moreover, AKR1C3 also metabolises the anti-cancer prodrug PR-104A; this is the first known example of nitroreductase activity by AKR1C3 [18].

A survey of the Protein Data Bank (PDB) reveals structures of AKR1C3 in complex with various molecules including NADP+ cofactor, 4-androsterone-3,17-dione, prostaglandin D2, and high affinity inhibitors [24]–[30]. The enzyme forms an α_8_β_8_ barrel structure with a large and multi-cavity active site that exhibits flexibility at both the level of individual side chains as well as entire loop regions upon ligand binding. The active site contains a conserved catalytic tetrad consisting of H117-Y55-K84-D50 with Tyr-55 acting as a general acid and base [31], as well as the oxidised cofactor, NADP+, deep in the site. The side chains of Tyr-55 and His-117 together with the nicotinamide moiety of the NADP+ molecule form an oxyanion site at which substrate carbonyl groups can bind for catalysis. However, a wealth of structural information and site-directed mutagenesis experiments, suggests a level of promiscuity in potential reductive mechanisms as illustrated by the putative prostaglandin reduction mechanisms proposed by Komoto *et al.*, 2006 [25]. The catalytic mechanism for PGD_2_ 11-ketoreduction comprises the donation of a proton from Tyr-55 or His-117 to the O11 carbonyl of PGD_2_ and the *pro-4R* hydrogen of NADPH is transferred directly to C11 forming 9α,11β-PGF_2_. For PGH_2_ 9,11-endoperoxide reduction, there is no direct involvement of enzyme active site residues in the hydrogen transfer. Here, the PGH_2_ peroxide binds near to the NADPH cofactor and receives the *pro-4R* hydrogen directly; the peroxide bond breaks in a concerted manner. The resulting negatively charged oxygen is protonated from solvent and PGF_2α_ is formed. Structurally characterised AKR1C3 inhibitors bind either at the oxyanion site directly or adjacent to this site, effectively blocking the productive binding of substrate molecules.

Non-steroidal anti-inflammatory drugs (NSAIDs) have been well characterised as potent AKR1C3 inhibitors [32]–[34]. These molecules affect anti-inflammatory and analgesic action as well as side-effects of gastrointestinal irritation through cyclooxygenase inhibition and blockade of downstream prostanoid species [35]–[37]. NSAIDs have been investigated for their anti-proliferative effect through their cyclooxygenase binding and also through multiple other mechanisms including AKR1C3 inhibition [33], [37], [38]. The NSAIDs bind to aldo-keto reductase (AKR) isoforms 1C1, 1C2, and the current focus, 1C3, with varying selectivity [32], [34]. Recent AKR1C3 inhibitor design efforts have focussed on two molecular templates derived from flufenamic acid and indomethacin [29], [34]. These have produced potent inhibitors with significant AKR1C3 selectivity. However, the indomethacin, flufenamic acid, and derivative templates, provide the only atomic-level structural information for structure-guided inhibitor design of NSAID analogues despite the wealth of information describing NSAID inhibition from *in vitro* activity assays.

Here we report ten new crystal structures of NSAIDs bound to AKR1C3 that cover three classes of compound, *N*-phenylanthranilic acids (meclofenamic acid, mefenamic acid), arylpropionic acids (flurbiprofen, ibuprofen, naproxen), and indomethacin analogues (indomethacin, sulindac, zomepirac), that provide unique insight into ligand binding and greatly broaden the base of structures available for future drug discovery efforts.

## Materials and Methods

### Expression, Purification, and Enzymatic Activity of AKR1C3

The AKR1C3 DNA sequence was purchased from Genscript Inc. sub-cloned into NdeI and XhoI sites of the pET21b vector (Merck). The C-terminal his-tagged protein was produced by leaky expression in Terrific Broth supplemented with ampicillin (100 mg/l); overnights were transferred into 500 ml of media and incubated at 37°C with shaking at 160 rpm for 16–18 hours. Cells were harvested by centrifugation at 5000 g for 30 min at 4°C and resuspended in buffer A (40 mM Tris. HCl pH 7.5, 20% glycerol, 0.8% octyl β-D-glucopyranoside, 1 mM NADP+, and 1 complete, EDTA-free protease inhibitor tablet (Roche) per 50 ml volume). Cells were lysed by sonication and the lysate centrifuged at 16,000 rpm for 25 minutes. Supernatent was applied to an immobilised metal (Ni^2+^) affinity chromatography (IMAC) column pre-equilibrated with buffer B (20 mM Tris. HCl pH 7.5, 10% glycerol, 150 mM NaCl) and eluted using a linear imidazole gradient (0–0.5 M). On elution, fractions containing AKR1C3 protein were immediately diluted 1∶4 with buffer C (20 mM Tris. HCl pH 7.5, 10% glycerol, 0.5 mM EDTA, 1 mM DTT) and were applied to a Blue Sepharose affinity column pre-equilibrated with buffer C. Protein was eluted by a linear NaCl gradient (0–2 M). The protein was stored at 4°C overnight before buffer exchanging into buffer D (10 mM potassium phosphate buffer pH 7.0, 1 mM EDTA, 1 mM DTT, 0.005% decyl maltoside, 1.2 mM NADP+) and concentrating to 25 mg/ml using a 30 kDa MWCO spin column (Vivascience). Steady-state kinetic parameters against 9,10-phenanthrenequinone were determined using a loss of NADPH assay by measuring the absorbance of the system at 340 nm. The assay condition comprised 0–1000 nM 9,10-phenanthrenequinone, 40 nM AKR1C3, 200 µM NADPH, in 100 mM sodium phosphate buffer pH 7.5. Initial reaction velocities were measured for 5 minutes at 20°C and fitted to a Michaelis-Menton plot to determine V_max_ and K_m_.

### Crystallization and Data Collection

A fresh protein sample was spiked with an equimolar amount of octyl β-D-glucopyranoside and subjected to hanging drop vapour diffusion crystallization trials. A 1 µL volume of protein was mixed with 1 µL of a crystallization solution consisting of 200 mM sodium acetate and 20% PEG 3350, and was placed over a reservoir volume of 0.5 ml. Large, well-diffracting crystals appear within 5 days and grow to maximum dimensions of 0.1×0.1×0.4 mm. Meclofenamic acid, mefenamic acid, (*R*)/(*S*)-flurbiprofen, (*R*)/(*S*)-ibuprofen, (*S*)-naproxen (Sigma-Aldrich Co. LLC) and (*R*)/(*S*)-naproxen (Santa Cruz) were dissolved in DMSO and added at a final concentration of 5 mM to crystals in a 2 µl drop of crystallization solution. The crystals were soaked for a minimum of 3 days before being briefly dipped in a cryoprotectant solution (200 mM sodium acetate, 20% PEG 3350, 20% ethylene glycol) before flash cooling in liquid nitrogen. Data was collected on the Australian synchrotron beamline MX2 using the BlueIce software package [39] or on a Rigaku MicroMax-007HF rotating anode instrument equipped with Mar345dtb detectors. Statistics are summarized in Table 1.

**Table 1:** Crystal properties and data collection.

	Meclofenamic acid	Mefenamicacid	(*R*)-Flurbiprofen	(*R*)-Ibuprofen	(*R*)-Naproxen	Indomethacin pH 7.5	Zomepirac	Sulindac
Space group	*P*2_1_2_1_2_1_	*P*2_1_2_1_2_1_	*P*2_1_2_1_2_1_	*P*2_1_2_1_2_1_	*P*2_1_2_1_2_1_	*P*2_1_2_1_2_1_	*P*2_1_2_1_2_1_	*P*2_1_2_1_2_1_
Unit-cell parameters*a*, *b*, *c* (Å)	58.32, 64.79,96.28	58.28, 64.56,96.60	56.87, 63.77,95.67	57.79, 64.53,96.18	58.55, 64.58,96.56	56.66, 63.94,96.52	57.29, 64.04,95.98	58.38, 64.48,96.85
Beamline	AS MX-2	AS MX-2	AS MX-2	AS MX-2	Rotating anode	Rotating anode	AS MX-2	AS MX-2
Resolution	1.95 (2.06–1.95)	2.00 (2.11–2.00)	2.00 (2.11–2.00)	1.80 (1.90–1.80)	1.90 (2.00–1.90)	1.73 (1.82–1.73)	1.90 (2.00–1.90)	2.10 (2.21–2.10)
Wavelength (Å)	0.97941	0.97941	0.97941	0.97941	1.541799	1.541799	0.97941	0.97941
*R*_merge_ †	0.082 (0.610)	0.115 (0.673)	0.177 (0.531)	0.104 (0.705)	0.090 (0.881)	0.086 (0.646)	0.109 (0.705)	0.118 (0.614)
Completeness (%)	99.8 (99.7)	99.9 (100.0)	99.1 (98.3)	99.9 (99.9)	100.0 (100.0)	96.7 (91.6)	98.7 (98.1)	99.9 (100.0)
Observed reflections	197246	363587	345079	417231	1145663	889385	410545	156593
*<I/σ(I)>*	17.2 (3.3)	20.5 (4.5)	10.4 (4.5)	17.7 (3.8)	37.1 (5.5)	26.8 (4.6)	21.2 (4.9)	13.7 (3.6)
Multiplicity	7.2 (7.4)	14.4 (14.6)	14.5 (14.9)	12.2 (12.5)	38.8 (38.2)	24.7 (14.9)	14.6 (14.9)	7.1 (7.3)
Wilson *B* (Å^2^)	24.5	23.2	25.4	21.9	26.6	21.5	19.9	24.9

Data for the high resolution shell are shown in parentheses.

† *R*_merge_ = Σ*_hkl_*Σ*_i_* | *I_i_*(*hkl*) − <*I*(*hkl*)> |/Σ*_hkl_*Σ*_i_* I*_i_*(*hkl*), where *I_i_*(*hkl*) is the intensity of the *i*th measurement of an equivalent reflection with indices *hkl*.

### Structure Determination, Refinement, and Model Quality

Data were processed using *XDS* [40] and scaled with *SCALA* [41], and structures determined by molecular replacement using *PHASER* [42] with the AKR1C3/NADP+ structure PDB ID 1S1P as the search model. Models were completed with several cycles of manual building with *Coot* [43] and refinement with *REFMAC* [44]. The active site electron density was closely inspected and the appropriate NSAID molecules, built using the Dundee *PRODRG2* server [45], were fitted and refined by real space refinement. Solvent molecules were added by automatic peak picking from an Fo-Fc electron density map using *Coot*. Peaks above 3σ were selected and water molecules were manually checked in *Coot*. The final structures were refined with *REFMAC* TLS (translation libration screw) paired with maximum-likelihood restrained refinement. Stereochemistry of the final structures was evaluated using the *Molprobity* server [46]. Final refinement statistics are given in Table 2.

**Table 2:** Crystallographic refinement details.

	Meclofenamic acid	Mefenamic acid	(*R*)-Flurbiprofen	(*R*)-Ibuprofen	(*R*)-Naproxen	Indomethacin pH 7.5	Zomepirac	Sulindac
Resolution range	19.8–1.95	19.8–2.00	19.4–2.00	19.7–1.80	53.7–1.90	53.3–1.73	19.5–1.90	19.8–2.10
Reflections used	24437	22648	22082	30547	27977	34149	25152	19680
*R* factor	0.175	0.185	0.175	0.174	0.173	0.172	0.164	0.182
*R*_free_	0.206	0.226	0.220	0.213	0.194	0.200	0.204	0.232
Average *B* factor (Å^2^)	25.7	22.2	24.9	21.1	27.4	24.5	20.0	24.4
R.m.s. Bond lengths (Å)	0.026	0.021	0.023	0.024	0.026	0.026	0.026	0.024
R.m.s. Bond angles (°)	2.00	1.73	1.94	1.88	1.95	2.13	2.01	1.89
Ramachandran plot favoured (%)	97.3	97.7	9.73	97.7	97.3	97.0	96.7	97.7
Missing density (unmodeled)	1–5, 125–137, 322–331‡	1–5, 132–137, 322–331‡	1–5, 125–137, 322–331‡	1–5, 125–137, 322–331‡	1–5, 125–137, 321–331‡	1–5, 125–137, 322–331‡	1–5, 125–137, 322–331‡	1–5, 125–137, 322–331‡
PDB code	3R6I	3R43	3R94	3R8G	3UFY	3UG8	3R8H	3R7M

‡ Includes the C-terminal hexahistidine tag.

### Ligand Docking

Molecular docking was performed using the X-ray crystal structures of AKR1C3 with indomethacin and flufenamic acid bound (PDB codes 1S2A and 1S2C respectively). In preparing the structures for docking, waters were removed and the structures protonated using SYBYL8.0.3 (TRIPOS). The structures were then visually inspected for errors in protonation states. The side chain of His117 was modified allowing the NE2 donor hydrogen to form part of the oxy-anion pocket. Ligands were docked into the AKR1C3 active site with GOLDv5.1 using a docking cavity of 18 Å centered on the hydride transfer site of NADP in chain A in each structure. The docking was performed in the presence of NADP, using the GoldScore fitness function, with 10 poses per ligand saved. The search efficiency was set at 200%, while the ligand flexibility options “flip_all_planar nitrogens” and “flip protonated carboxylic acids” were set to flip, “match_ring_templates” was set to on, as was the “solvate all”. The remaining ligand flexibility settings were turned off and all other remaining settings were left as default. Both protein and ligand atom types were automatically assigned by GOLD. New crystal structures were superimposed onto the indomethacin (PDB: 1S2A) and flufenamic acid structures (PDB: 1S2C) using the method implemented in Hermes 1.5 (Cambridge Crystallographic Data Centre). The results were visually inspected for poses that showed good agreement with the known binding modes. RMSD values for predicted versus actual binding mode were generated using the “smart rms” utility implemented in GOLD 5.1.

## Results and Discussion

### Overall Structure and Active Site Sub-pockets

**Figure 2:**
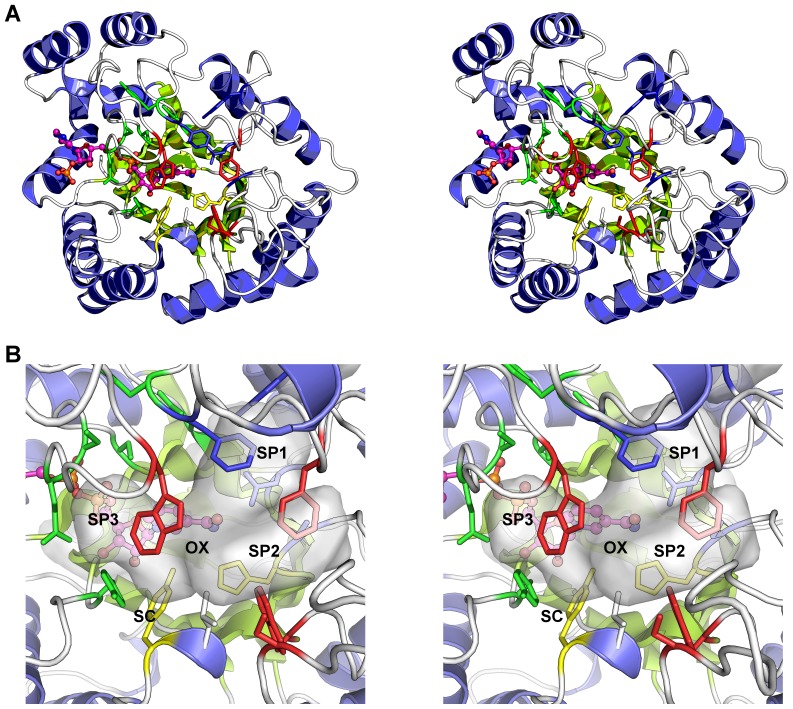
Overall structure and active site of AKR1C3. **A.** Cartoon diagram of the protein structure highlighting the active site residues - shown as multicoloured sticks. The NADP+ molecule is shown as a ball and stick figure with carbon atoms coloured magenta. Alpha helices are coloured blue and beta sheets in green. **B.** Close up view of the active site showing the sub-pocket structure as a semi-transparent surface. The residues lining sub-pocket 1 (SP1) are coloured blue, those lining sub-pocket 2 (SP2) are coloured red, and the sub-pocket 3 (SP3) residues are coloured green. Residues Tyr-55 and His-117 (in yellow sticks), along with the NADP+ molecule, form the oxyanion site (OX), and the steroid channel (SC), the binding site of steroid molecules, is “gated” by residues Trp-227 (foreground, red sticks) and Leu-54 (white sticks). Figures drawn using Pymol v1.3 incentive (Schrödinger, LLC).

Human AKR1C3 protein was expressed in *E. coli*, purified by affinity chromatography, and catalytic activity assayed with 9,10-phenanthrenequinone (Figure S1 inset) in the presence of NADPH by following the decrease in absorbance at 380 nm resulting from NADPH consumption. A K_m_ of 200 nM was retrieved from fitting activity data (Figure S1) using the Michaelis-Menten equation as implemented in GraphPad Prism 5.03 for Windows (GraphPad Software, San Diego California USA, www.graphpad.com), and is comparable to published data [4]. The catalytically active protein was crystallised, and soaked with three different classes of NSAID inhibitors, and structures were determined using molecular replacement in the space group *P*2_1_2_1_2_1_ with one protein molecule in the asymmetric unit. In all structures, the AKR1C3 protein shows its canonical α_8_β_8_ barrel core with an active site formed by the loop structure located at the C-terminal end of the barrel (Figure 2A), while one molecule of NADP+ and one NSAID molecule were clearly described by electron density present in the active site near the catalytic residues Tyr-55 and His-117.

Superimposition of human AKR1C3 onto the human 1C1, 1C2, and 1C4 isoforms shows the largest active site for AKR1C3 protein with multiple sub pockets used by a broad range of substrates and inhibitors to interact with the enzyme (Figure 2B). These sub pockets were recently annotated SP1, SP2, SP3, the oxyanion site (OX), and steroid channel (SC) [33]. SP1 is delineated by residues Ser-118, Asn-167, Phe-306, Phe311, and Tyr-319 and is occupied in all currently available crystal structures with small molecule ligands bound. SP2 is formed by residues Trp-86, Ser-129, Trp-227, and Phe-311 and is only occupied by the 8α chain of PGD_2_ (shallow binding) and the 12β chain of the inhibitor bimatoprost (deep binding). In all the structures described herein, some or all of the residues in the flexible loop located between residues 125 and 137 were not modelled due to disorder; the SP2 pocket is partly formed by this flexible loop that is often not observed in crystal structures unless crystal packing or ligand binding stabilise the loop structure. The SP3 sub-pocket is formed by residues Tyr-24, Glu-192, Ser-217, Ser-221, Gln-222, Tyr-305, and Phe-306, and only the NSAID indomethacin binds in this pocket. Three residues, Trp-227, Phe-306, and Phe-311, exhibit considerable side chain flexibility adopting different conformations depending on the ligand bound. The conformation of Trp-227 defines the size of SP2 and the steroid channel, while Phe-311 lines either SP1 or SP2 depending on the ligand present. Rotation of Phe-306, usually in concert with Phe-311, allows SP3 to be accessed. The oxyanion site is formed around the active site residues Tyr-55, His-117 and the NADP+ cofactor, and is the catalytic site at which aldehyde or ketone reduction occurs. The binding of carboxylic acid or ketone groups of NSAIDs at the oxyanion site explains the general inhibitory effect on AKR1C proteins. The steroid channel is a conserved feature of all AKR1C proteins and describes the open channel that leads to solvent space and is gated by residues Trp-227 and Leu/Val-54. It is the general binding site of the steroid substrate molecules and the high-affinity steroid-like inhibitor EM1404.

### Binding Modes for the *N*-phenylanthranilic Acids Flufenamic Acid, Meclofenamic Acid and Mefenamic Acid

**Figure 3:**
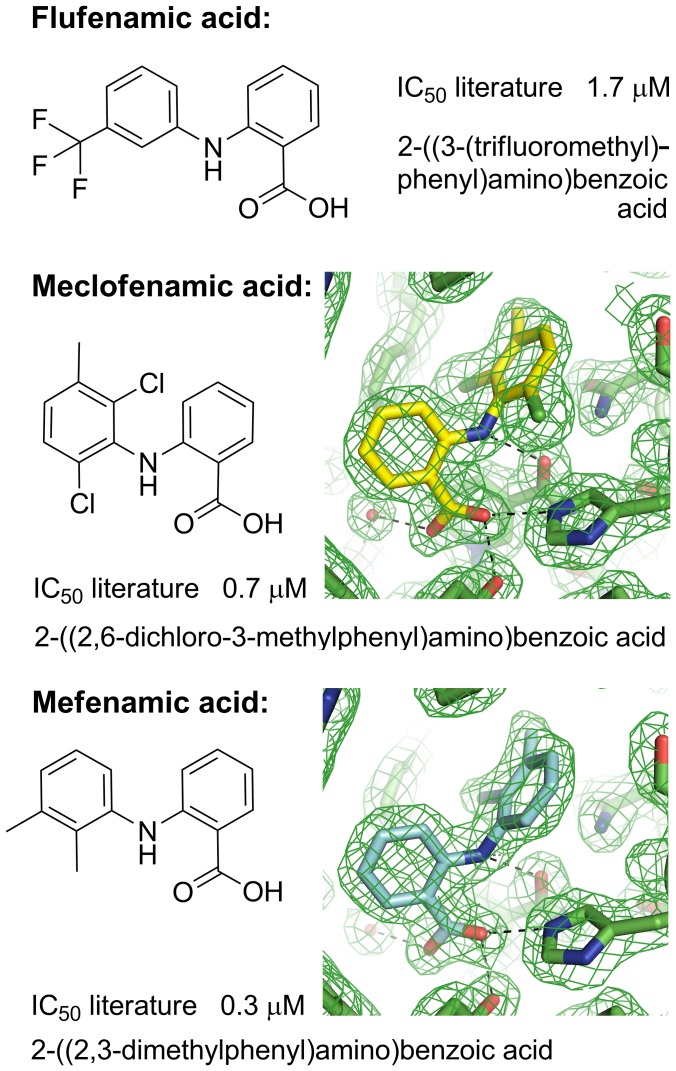
Molecular structures, systematic names, and IC_50_ values against AKR1C3 for the *N*-phenylanthranilic acids (flufenamic acid, meclofenamic acid, mefenamic acid). Electron density maps (2*F*o-*F*c omit maps drawn at the 1σ level) are shown for meclofenamic acid and mefenamic acid active sites (see Figures S2A and S2B for larger versions of the maps). The ligands are modelled at full occupancy. Figures drawn using ChemBioDraw Ultra 12.0 (CambridgeSoft) and Pymol v1.3 incentive (Schrödinger, LLC). IC_50_ values taken from Byrnes and Penning, 2009 [32].

These *N*-phenylanthranilic acid molecules contain a common core of 2-(phenylamino)benzoic acid with various substituents at positions 2,3,5, and 6 of the phenyl ring (Figure 3). Flufenamic acid is a 3-(trifluromethyl) derivative, meclofenamic acid a 2,6-dichloro and 3-methyl derivative, and mefenamic acid a 2,3-dimethyl derivative.

**Figure 4:**
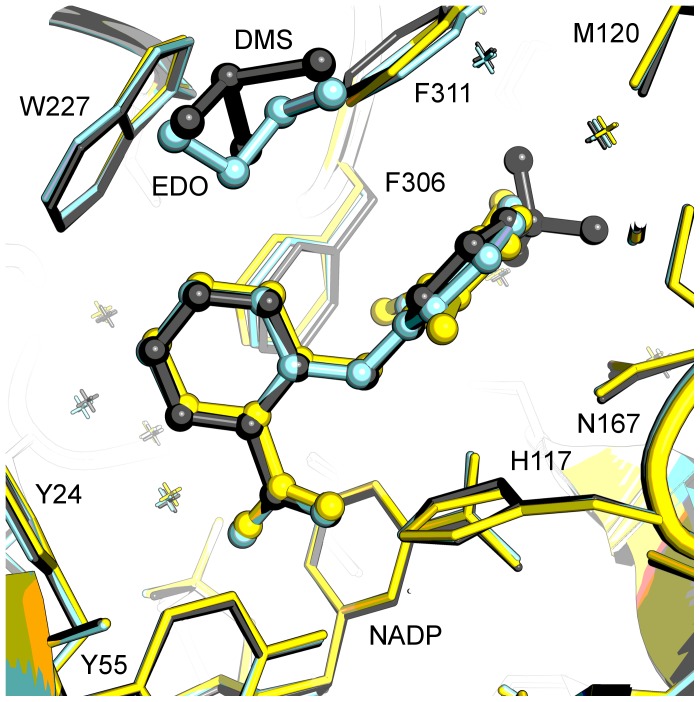
Structure-based overlay of *N*-phenylanthranilic acid ternary complexes. The overlay displays active sites for flufenamic acid (grey; PDB 1S2C), meclofenamic acid (yellow), and mefenamic acid (cyan). Protein side chains are displayed as sticks, drug molecules, dimethylsulfoxide (DMS), and ethylene glycol (EDO) are displayed as ball and stick models. Figure drawn with Pymol v1.3 incentive (Schrödinger, LLC).

The structure of flufenamic acid bound to AKR1C3 has previously been determined at a resolution of 1.8 Å [26]. The most important binding interaction, resulting in the inhibitory effect, is the hydrogen bonding between the drug carboxylic acid atom O1 and the oxyanion site residues Tyr-55 and His-117. The other oxygen atom of this group forms a hydrogen bond to an adjacent water molecule, part of an integral and conserved water network seen in other structures of the enzyme. The carboxylic acid group packs flatly against the nicotinamide ring of NADP+. The ring-linking amine interacts with the NADP+ nicotinamide oxygen with favourable hydrogen bond geometry. The drug CF_3_ group occupies the SP1 active site pocket and hydrogen bonds to a water molecule and to the hydroxyl group of Tyr-216. The two aromatic rings of the drug molecule are held in place by van der Waals interactions particularly by face and edge-stacking interactions with aromatic protein side chains; the benzoic acid ring interacts primarily with side chains Tyr-24, Trp-227, and Phe-306, while the phenylamine ring is surrounded by side chains Trp-86, Asn-167, and Phe-311 (Figure 4). A DMSO molecule from the solvent system binds in an adjacent pocket to the drug and forms van der Waals interactions with side chains Trp-86, Ser-129, Trp-227, Phe-311, benzoic acid (drug), and forms a single hydrogen bond to a two water network. The active site configuration is very similar to PDB 1S1P (containing PEG & acetate in the active site), with little movement of active site side chains to accommodate the drug molecule.

We have determined structures of flufenamic acid analogues meclofenamic acid and mefenamic acid bound to AKR1C3 at 1.9 and 2.0 Å respectively (Figure 3, Tables 1 and 2) by soaking preformed protein crystals with each compound dissolved in DMSO. The molecules have been fitted into clearly defined and unambiguous electron density and display a single binding location, orientation, and conformation. Meclofenamic and mefenamic acid molecules bind very similarly to flufenamic acid, hydrogen bonding the oxyanion site (Tyr-55 and His-117) through their carboxylic acid group and extending into the SP1 pocket and with a close overlay (Figure 4). The three structures share a well-conserved pattern of hydration. Where the flufenamic acid structure contains a DMSO molecule from the solvent in a pocket adjacent to the drug molecule, the mefenamic acid structure contains a molecule of cryoprotectant ethylene glycol; the meclofenamic acid structure contains no solvent or cryoprotectant molecule in this site. The DMSO/ethylene glycol pocket might be an exploitable site for future drug development. In the SP1 pocket of the three compared structures, minor shifts in the side chains of residues Phe-306 and Phe-311 are required to accommodate the different substituent groups of the phenyl rings. This high degree of similarity in molecular structure and binding mode is reflected in the similar IC_50_ values reported for these ligands (flufenamic acid 1.7 µM, meclofenamic acid 0.7 µM, mefenamic acid 0.3 µM) [32]. A full list of intermolecular contacts in the meclofenamic and mefenamic acid structures is given in Tables S2 and S3 and illustrated in Figures S3 and S4.

### Binding Modes for the Arylpropionic Acids Flurbiprofen, Ibuprofen, and Naproxen

**Figure 5:**
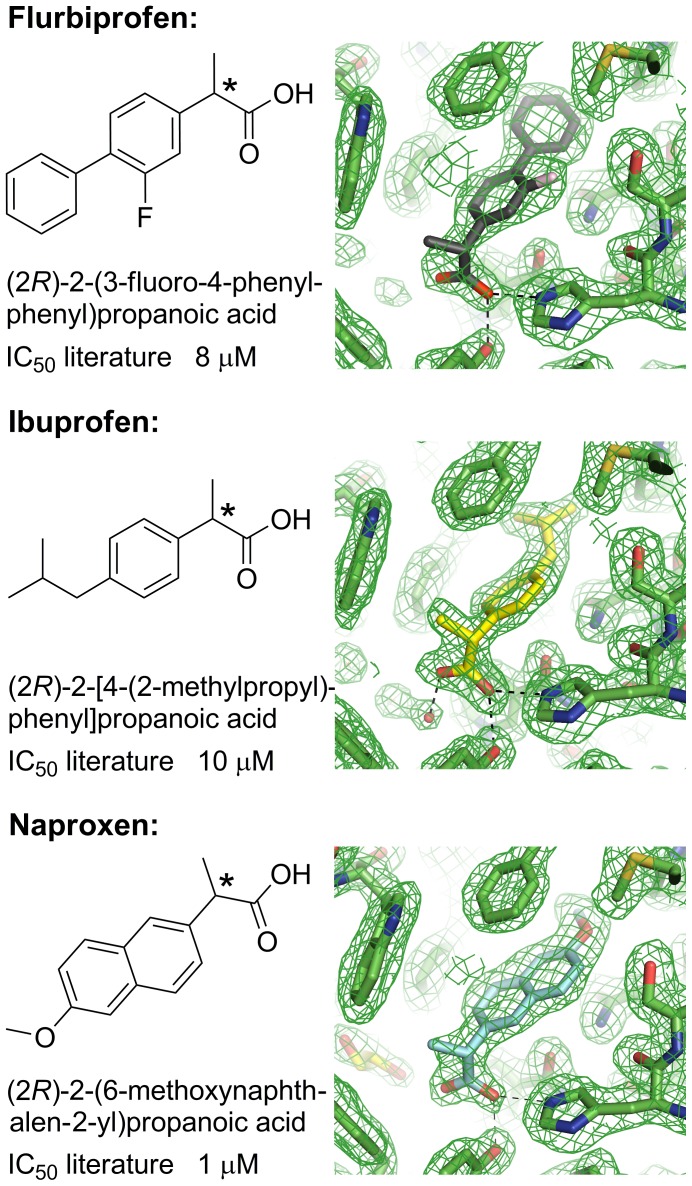
Molecular structures, systematic names, and IC_50_ values against AKR1C3 for the arylpropionic acids (flurbiprofen, ibuprofen, naproxen). The chiral centre of each molecule is labelled with an asterisk. Electron density maps (2*F*o-*F*c omit maps drawn at the 1σ level) are shown for each active site (see Figures S2C, S2D and S2E for larger versions of the maps). The ligands are modelled at full occupancy. Figures drawn using ChemBioDraw Ultra 12.0 (CambridgeSoft) and Pymol v1.3 incentive (Schrödinger, LLC). IC_50_ values taken from Byrnes and Penning, 2009 [32].

These inhibitor molecules contain the common core of 2-phenylpropanoic acid that differ in the substitution of the phenyl ring (Figure 5). Flurbiprofen has 3-fluoro and 4 phenyl substituents, ibuprofen has a 4-(2-methylpropyl) substituent. Naproxen deviates most from the core structure in that it contains a fused 2 ring system and is then best described as a 6-methoxynaphthalene derivative. The core propanoic acid is chiral at the 2 position as indicated in Figure 5.

We determined the structures of flurbiprofen, ibuprofen, and naproxen bound to the AKR1C3 protein at 2.0, 1.8, and 1.9 Å respectively (Figure 5, Tables 1 and 2). The drug molecules were fitted into well-defined electron density that did not show any signs of disorder or multiple binding modes (Figure 5). In each case, a racemic mixture of *R-* and *S-*enantiomer was soaked into preformed protein crystals. In all three structures the protein selects the *R-*enantiomer, the opposite enantiomer to that which binds COX-1 and COX-2. Naproxen was also soaked into crystals as an enantiomerically pure *S-* molecule and showed clear binding in the active site (Figure S2I and Table S1).

**Figure 6:**
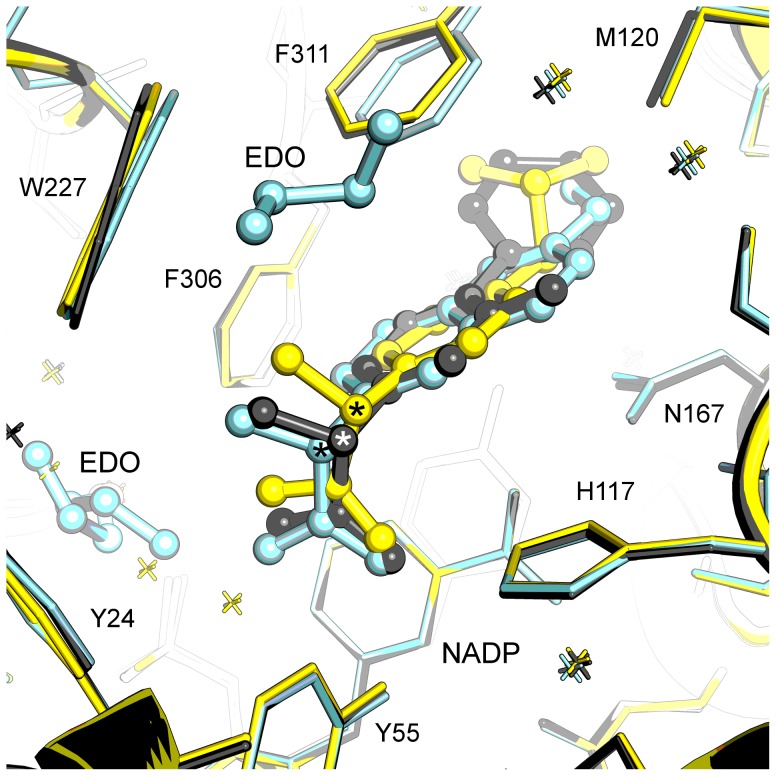
Structure-based overlay of arylproprionic acid ternary complexes. The overlay shows active site for flurbiprofen (grey), ibuprofen (yellow), naproxen (cyan) active sites. Protein side chains are displayed as sticks, drug molecules, and ethylene glycol (EDO) are displayed as ball and stick models. The chiral centre of each drug molecule is labelled with an asterisk. Figure drawn with Pymol v1.3 incentive (Schrödinger, LLC).

An overlay of the three arylproprionic acid NSAIDs in the active site shows similar binding modes that like the *N-*phenylanthranilic acids, have their carboxylic acid group binding the oxyanion site residues Tyr-55 and His-117 through hydrogen bonding, and with the remainder of the molecule extending into the SP1 pocket (Figure 6). While the carbonyl oxygen of each molecule binds in the same location, the remainder of each molecule rotates from this “fixed” point. This results in a poor overlay of the propionic acid moieties although the variable molecular structures beyond this point occupy very similar space in the binding pocket; specifically, the variable structures extend to the same maximum depth in the SP1 pocket. Overall the active sites are very similar to PEG/acetate containing structures (1S1P and our own unpublished structures) as assessed by the side chain conformations of mobile residues Trp-227 and Phe-306. A more significant shift is seen in the third mobile residue Phe-311, which has shifted 1.2 Å in Cα coordinate compared with PDB 1S1P and has also undergone an approximately 90° rotation about the Cα-Cβ bond.

The SP1 binding depth and the oxyanion site interaction by a single oxygen atom appear to be the primary determinants of binding for this set of compounds. While the variable structures extend to the same depth their rings are not coplanar and these differences are accommodated through minor rearrangement of Trp-227 and Phe-311 side chains. The naproxen structure is unique in having a disordered cryoprotectant ethylene glycol molecule occupy the SP3 pocket – this location in the other two structures contains conserved water molecules that overlay the ethylene glycol molecule closely. The naproxen active site also contains an ethylene glycol molecule located similarly to the organic solvent molecules of flufenamic and mefenamic acid structures. The reported IC_50_ values [32] for the three molecules are comparable (flurbiprofen 8 µM, ibuprofen 10 µM, naproxen 1 µM) and this is also reflected in their similar binding modes in the active site of the enzyme. Naproxen inhibition appears better by a factor of ∼10 but the cause of this is not obvious from a comparison of our structures or from analysis of the full list of protein-ligand contacts (Tables S4, S5, and S6 and illustrated in Figures S5, S6, and S7).

We observe that AKR1C3 exhibits a distinct preference in stereoisomers in this class, with the *R-*enantiomer from the racemic mixture of flurbiprofen, ibuprofen, or naproxen molecules captured in the active site. A detailed comparison of (*R*)- and (*S*)-naproxen AKR1C3 complexes shows subtle differences in the protein-ligand contacts in each system (Tables S6 and S11 and illustrated in Figures S7 and S11). The (*S*)-naproxen molecule displays 86 protein ligand contacts with 8 active site side chains and the NADP molecule, while (*R*)-naproxen makes fewer contacts at 78 but with 12 active site side chains and NADP. These intermolecular interactions translate to different percentages of the ligand surfaces in contact with the active site; (*S*)-naproxen shows a 384 Å^2^ complementary surface with the protein out of a theoretical 429 Å^2^, or 90% complementarity, while (*R*)-naproxen shows greater complementarity of 95% which might explain the subtle preference of AKR1C3 for the *R*-stereoisomer we observe in our crystallographic experiments.

Like many other racemic drugs, these NSAIDs display stereoselectivity in their action. They bind COX-2 as the *S-*enantiomers giving the well-characterised anti-inflammatory effect [47], [48] although a recent report has shown the *R*-enantiomers of flurbiprofen, ibuprofen and naproxen are also active against COX-2 and act as inhibitors of endo­cannabinoid oxygenation, and suggest a mechanism for the measurable analgesic effect of (*R*)-flurbiprofen [49], [50]. (*R*)-flurbiprofen and (*R*)-ibuprofen have been investigated for anticancer effect in prostate, ovarian, and various other cell lines [51], [52]. The anticancer mechanism is COX-2 independent and involves the induction of p75(NTR) tumour suppressing protein. Our crystal structures clearly show a preference of AKR1C3 for binding (*R*)-flurbiprofen, (*R*)-ibuprofen, and (*R*)-naproxen and while we cannot comment further on this stereoselectivity in relation to any anticancer effect, the structures do suggest further studies might be appropriate to determine if the anticancer activity of these compounds is due in some part to AKR1C3 inhibition.

### Binding Modes for Indomethacin and Analogues Sulindac and Zomepirac

**Figure 7:**
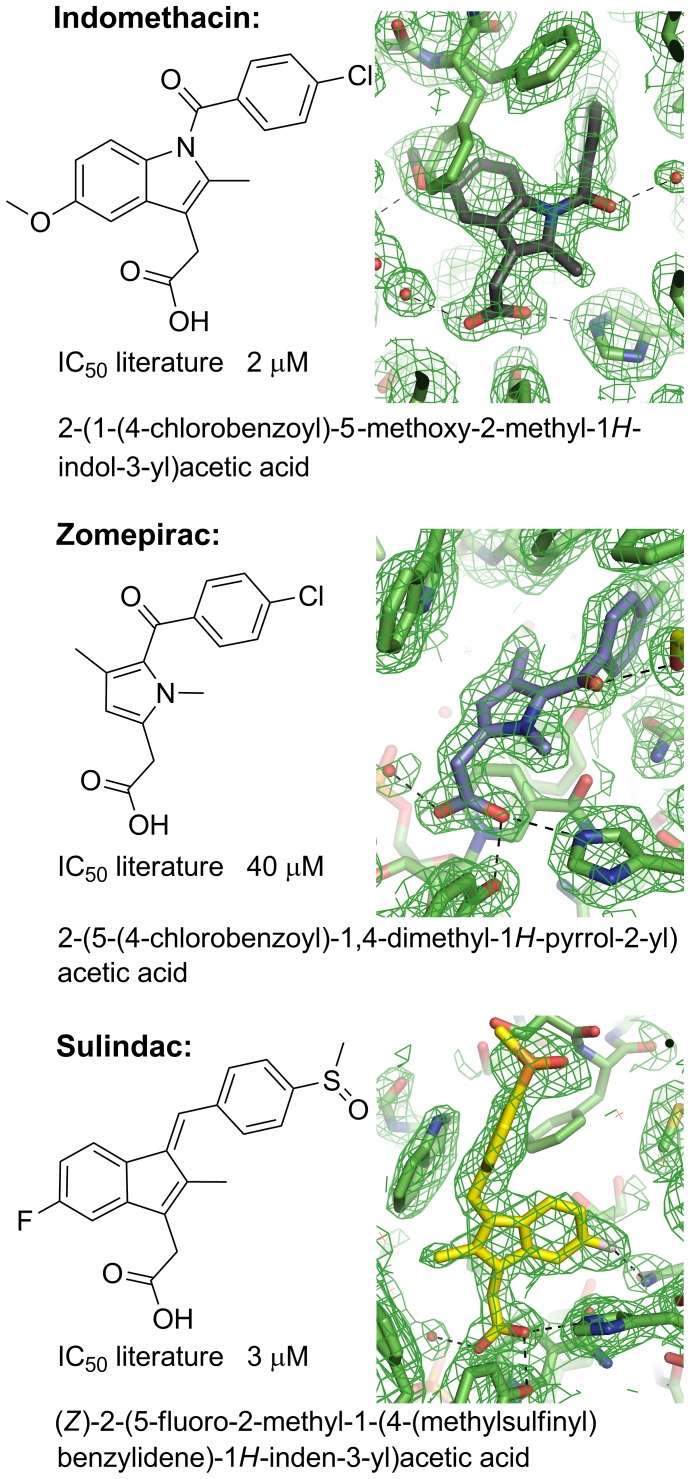
Molecular structures, systematic names, and IC_50_ values against AKR1C3 for the indomethacin analogues (indomethacin, sulindac, zomepirac). Electron density maps (2*F*o-*F*c omit maps drawn at the 1σ level) are shown for indomethacin (pH 7.5), sulindac and zomepirac acid active sites (see Figures S2F, S2G and S2H for larger versions of the maps). Indomethacin has been modelled at an occupancy of 0.7 and two alternative conformations of phenylalanine 306 are shown at complementary occupancies modelled at 0.3 and 0.7. Figures drawn using ChemBioDraw Ultra 12.0 (CambridgeSoft) and Pymol v1.3 incentive (Schrödinger, LLC). IC_50_ values taken from Byrnes and Penning, 2009 [32].

The final class of compound are more variable in the core structure than the previous examples (Figure 7). The common core might best be described as containing benzyl, cyclopentene, and acetic acid groups. Inter-ring linkers and ring substituent groups are clearly different and suggest an influence on protein binding and ligand conformation. The most different of the molecules is zomepirac where the central ring consists of a single 5-membered system rather than the fused 5/6-membered ring systems of indomethacin and sulindac.

We have determined the structures of indomethacin complexes at pH 6.8 (Table S1) and pH 7.5 (Figure 7, Tables 1 and 2) to compare with the previously published structure determined at a pH of 6.0. At pH 7.5 the indomethacin molecule can be fitted into clearly defined and unambiguous electron density at a refined occupancy of 0.7, and displays a single binding location, orientation, and conformation; the binding mode is clearly different to that seen at pH 6.0 [26]. At pH 6.8 we see a combination of the two binding modes. We further determined analogous structures with sulindac and zomepirac at 2.1 and 1.9 Å respectively (Figure 7, Tables 1 and 2). In both of these cases, the drug molecules have been fitted into clearly defined and unambiguous electron density and display a single binding location, orientation, and conformation. The three molecules, indomethacin, sulindac and zomepirac collectively display three different binding modes in the active site of AKR1C3 and this is discussed in detail below.

### Indomethacin Binding is Influenced by PH

**Figure 8:**
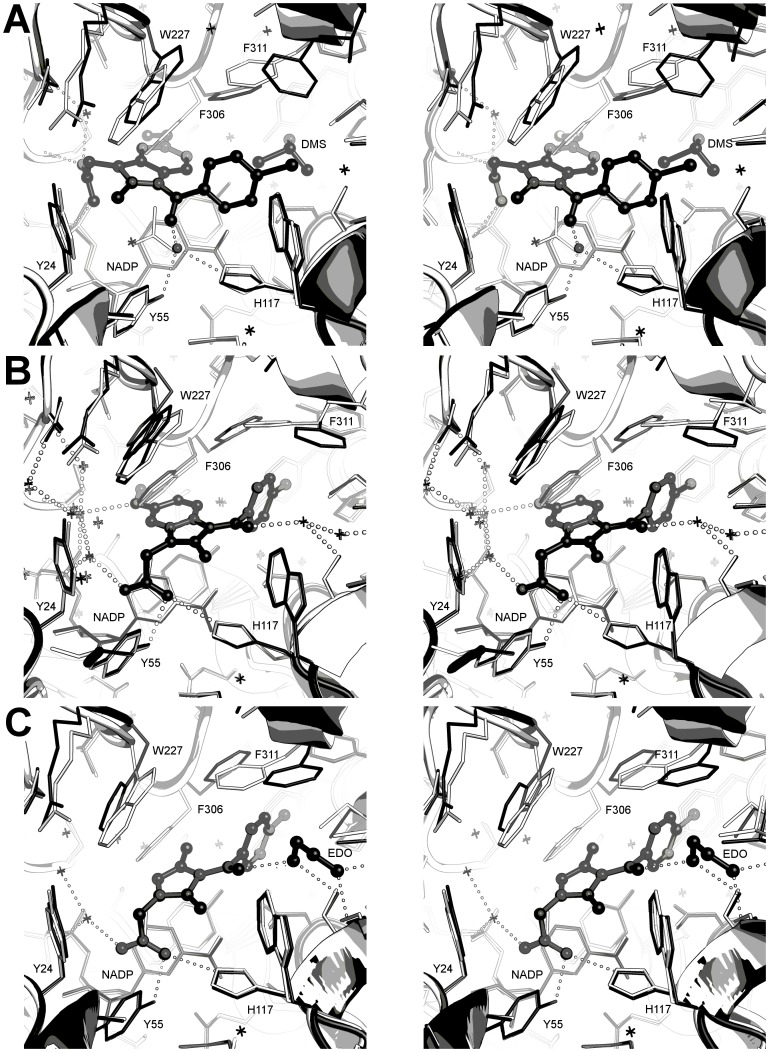
Stereo views of indomethacin analogues in the AKR1C3 active site. **A.** Overlay of PEG/acetate (1S1P; coloured white) and pH 6.0 indomethacin (1S2A; coloured grey) active sites showing the indomethacin hydrogen bonding pattern and protein side chain shifts. **B.** Overlay of PEG/acetate (1S1P; coloured white) and pH 7.5 indomethacin (coloured grey) active sites showing the indomethacin hydrogen bonding pattern and protein side chain shifts. Two alternative conformations of phenylalanine 306 are shown; where indomethacin occupies the active site (only 70% of the molecules in the crystal) clearly the 30% occupancy side chain of phenylalanine 306 cannot co-exist in the same space. **C.** Overlay of PEG/acetate (1S1P; coloured white) and zomepirac (coloured grey) active sites showing the zomepirac hydrogen bonding pattern.

The structure of indomethacin bound to AKR1C3 has previously been determined at a pH of 6.0 and at a resolution of 1.7 Å [26]. The indomethacin molecule is located in the SP3 pocket with its carboxylic acid binding to a phosphate group of NADP rather than at the oxyanion site (Figure 8A). Indomethacin atom O3 hydrogen bonds to O2N and O1N of NADP while the O2 atom hydrogen bonds to the backbone nitrogen of Gln-222 and to an adjacent water molecule. The carboxylic acid group displaces two water molecules found in the PEG/acetate structure 1S1P. The indole ring extends into a pocket normally occupied by Phe-306 in the PEG/acetate structure; Phe-306 swings away by an approximately 110° rotation about the Cα-Cβ bond with the concerted movement of Phe-311 by a rotation of ∼90° about the Cα-Cβ bond. Trp-227 is also displaced by ∼1.3 Å but retains the PEG/acetate side chain orientation. The indole ring binds perpendicular to the NADP nicotinamide ring with the shortest contact distance of 3.3 Å. The inter-ring carbonyl displays a 40° torsion from the plane of the indole ring and binds closely above an “unknown” atom in the oxyanion site. The *p*-chlorobenzoyl ring is observed in a *cis* conformation and approximately perpendicular to the plane of the indole ring. This ring extends towards the SP1 pocket and makes few contacts to the protein.

The pH 7.5 indomethacin structure, by contrast with the pH 6.0 structure, binds the AKR1C3 active site in the ubiquitously occupied SP1 pocket and interacts with the oxyanion site through its carboxylic acid, consistent with other NSAIDs (Figure 8B). The carboxylic acid group overlays almost perfectly the acetate molecule of the PEG/acetate structure (1S1P) and packs tightly against the face of the NADP+ nicotinamide ring with a number of close contacts between 3.1 and 3.3 Å. The carboxylate oxygens overlay exactly a water molecule and the unknown atom of the pH 6.0 structure, and hydrogen bond to a water network in the SP3 pocket - the binding pocket of the pH 6.0 carboxylic acid. The methoxy oxygen of the indole ring also hydrogen bonds to the SP3 water network. The inter-ring carbonyl displays a 18° torsion from the indole ring plane and hydrogen bonds to a series of two water molecules that further hydrogen bond to the hydroxyls of Ser-87 and Ser-118, and to the backbone carbonyl of Met-120. Like indomethacin at pH 6.0, the *p*-chlorobenzoyl ring is observed in the *cis* conformation and with its ring plane approximately perpendicular to the indole ring plane. The *p*-chlorobenzoyl ring extends into the SP1 pocket making longer range (3.3 to 3.6Å) non-bonded contacts. A detailed list of intermolecular contacts in this structure is given in Table S7 and illustrated in Figure S8.

The indomethacin binding mode at pH 7.0 requires the displacement of the three mobile residues Phe-306, Phe-311, and Trp-227. The inhibitor indole ring displaces Phe-306 by an approximately 130° rotation about the Cα-Cβ bond and a 0.6 Å shift in Cα coordinate, and with the concerted movement of Phe-311 by a smaller rotation of ∼115° about the Cα-Cβ bond. The displacement of this pair of residues is greater than for the pH 6.0 structure, allowing the SP1 pocket to expand and accommodate the *p*-chlorobenzoyl ring. Trp-227 shows two conformations each at occupancies of 0.5. The first conformation closely overlays the PEG/acetate structure while the second conformation requires a displacement of Cα coordinate by 1.3 Å coupled with a 90° rotation about Cα-Cβ and 120° rotation about Cβ-Cγ.

Can we rationalise the two pH-dependent binding modes of indomethacin? Three pH-dependent variables could contribute to the observed differences; the drug exists in the carboxylic acid form at the lower pH rather than the carboxylate anion; the NADP phosphate group is protonated at the lower pH; the p*K*_a_ of the oxyanion hole changes in response to the pH [53] (or a combination of these factors). We have used the PROPKA server [54] to calculate p*K*_a_ values for all residues in the PEG/acetate structure (PDB 1S1P, minus acetate), indomethacin pH 6 structure (PDB 1S2A, minus UNK atom), and our indomethacin pH 7.5 structure - the results are tabulated in Table S8. The oxyanion site appears unresponsive to the pH change and appears protonated in all structures. The largest shift in predicted p*K*_a_ is seen at the NADP O1N/O2N phosphate oxygens. Here we see a large increase to 11.8 in the low pH 1S2A structure from values of 6.7–6.9 in the other two structures. This suggests the most dominant effect at the lower pH is the protonation of the phosphate group of NADP.

### Details of Zomepirac Binding in Sub-pocket 1

Zomepirac has very similar functional groups to indomethacin but lacks a full indole ring. It binds the AKR1C3 active site in the SP1 pocket and overlays the pH 7.0 indomethacin coordinates very closely (19 atoms overlay with an average 0.24 Å shift in atomic position), as illustrated in Figure 8C. The ring-linking carbonyl hydrogen bonds an ethylene glycol molecule that is placed with its oyxgen atoms exactly overlaying two water molecules of the pH 7.0 indomethacin and also hydrogen bonding to Ser-118 OG, Ser-87 OG, and Met-120 O. This solvent molecule also binds directly below the side chain of Phe-311 with two close contacts between atom O2 and the aromatic ring. A detailed list of intermolecular contacts in this sturcture is given in Table S9 and illustrated in Figure S9.

A number of active site side chain movements are required to accommodate the ligand. The dimethylpyrrole ring displaces Phe-306 and Phe-311 similarly to pH 7.0 indomethacin to avoid a clash with the dimethylpyrrole ring and to accomodate the bulky *p*-chlorobenzoyl ring. The largest conformational change observed in the protein structure is seen at Trp-227. This residue moves only 1.0 Å in the Cα coordinate but displays a 105° rotation about Cα-Cβ bond and a 130° rotation about Cβ-Cγ bond. The major rearrangements of the active site residues paired with the relatively small number of ligand-protein contacts might account for the high IC_50_ for zomepirac of 40 µM compared to 2 µM for indomethacin and 3 µM for sulindac.

### Details of Sulindac Binding in Sub-pocket 2

**Figure 9:**
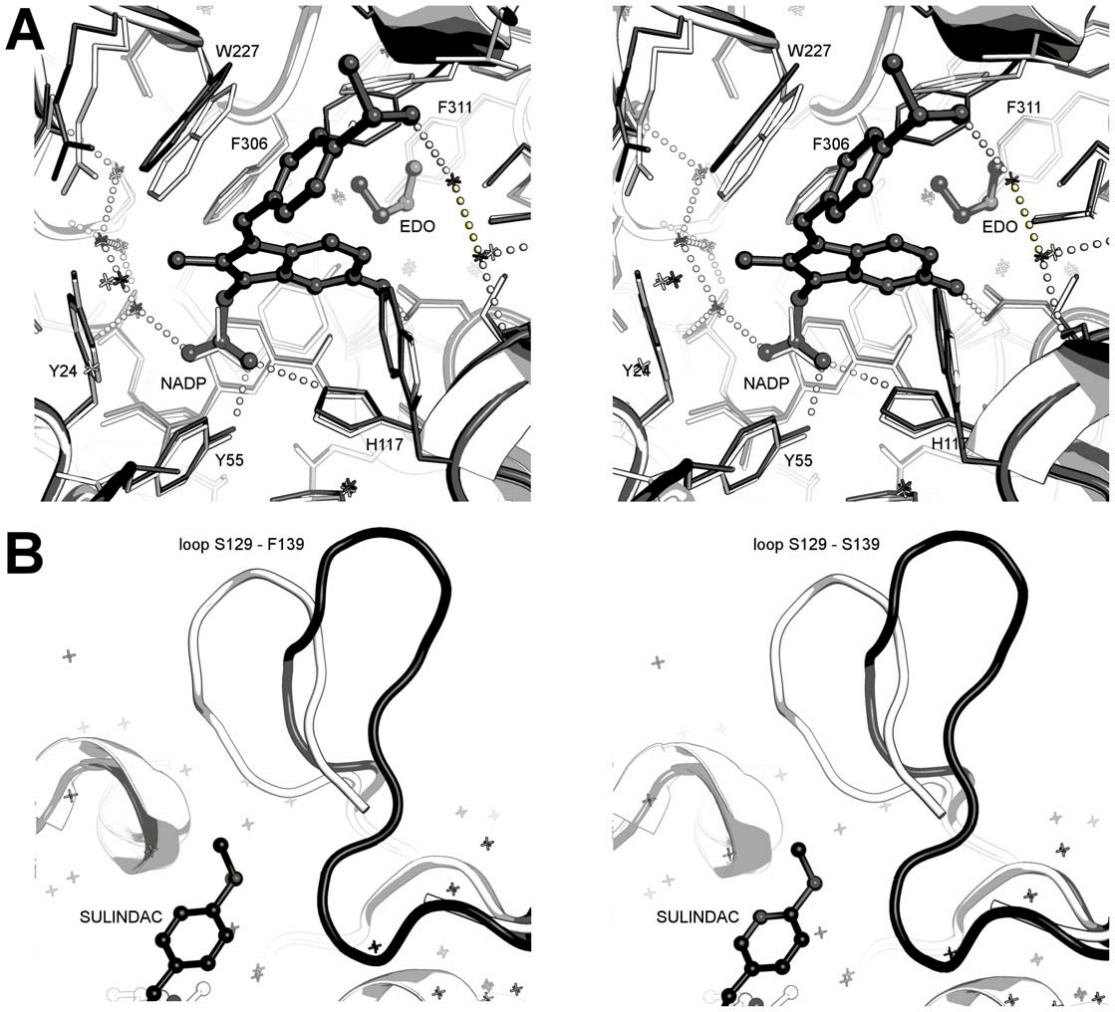
Stereo views of sulindac binding in the AKR1C3 active site. **A.** Overlay of PEG/acetate (1S1P; coloured white) and sulindac (coloured grey) active sites showing the sulindac hydrogen bonding pattern. **B.** Overlay of loop region between residues Ser-129 and Phe-139 for indomethacin (1S2A; white) and sulindac (grey) structures. Figures drawn with Pymol v1.3 incentive (Schrödinger, LLC).

The sulindac molecule has a comparable IC_50_ to indomethacin (3 uM vs 2 uM), and contains the same basic ring structure with some subsitutent changes and most critically, a change to the linker group between rings (in indomethacin a carbonyl and in sulidac an alkene linker) and inclusion of a sulfoxide unit. Strikingly, the sulindac binding mode shows little overlap with either of the indomethacin binding modes discussed here. The sulindac molecule binds within the SP2 site with greater binding depth than the natural substrate molecule PGD_2_, although not as deeply as the inhibitor bimatoprost. Like zomepirac and pH 7.0 indomethacin, the sulindac carboxylic acid binds in the oxyanion site and packed against the face of the NADP+ nicotimamide ring (Figure 9A). The indole ring extends towards the SP1 pocket with the F substituent forming a hydrogen bond to Asn-167 atom ND2. An ethylene glycol molecule binds deeper in the SP1 pocket in this structure closely overlaying the terminus of the PEG molecule of PDB 2FGB. The 4-(methylsulfinyl)benzyl ring displays an approximately 70° angle to the indole ring plane and extends into the relatively solvent exposed SP2 pocket. The sulfoxide group hydrogen bonds to a conserved water network in this pocket anchored by protein residues Ser-87, Ser-118, and Met-120. The attached benzyl ring makes a close contact of 3.1 Å to the side chain of Ser-129, part of a large loop structure that is displaced in this structure and is discussed further below. The three strong hydrogen bonding interactions involving the sulindac carboxylic acid, fluoride, and sulfoxide groups may account for much of the binding affinity that gives a similar IC_50_ to indomethacin. We should note however, that sulindac is a prodrug whereby in the human gastrointestinal tract the sulfoxide group is reduced to a sulphide – this may have some impact on binding *in vivo* relative to our *in vitro* experiments. A detailed list of intermolecular contacts in this structure is given in Table S10 and illustrated in Figure S10.

Compared to the indomethacin and zomepirac structures, the sulindac structure shows little movement in residue Phe-306 from the PEG/acetate 1S1P structure. A shift of ∼1 Å is seen in Trp-227 to avoid clashing with the indole methyl substituent, the inter-ring linker and the benzyl ring, and a slightly smaller shift is observed in Trp-86 to accommodate indole and benzyl ring edges. A larger displacement is found for Phe-311 with a Cα coordinate shift of 1.3 Å coupled with a rotation of 90° about Cα-Cβ. The largest shift seen in this structure is in the loop feature between residues Ser-129 and Phe-139 (Figure 9B). The loop is displaced at the tip by ∼7 Å relative to the indomethacin or PEG/acetate structure (PDB 1S1P) and assumes a very similar location and conformation to the loop in the PGD_2_ structure (PDB 1RY0) where the ligand extends into this same SP2 pocket. The bimatoprost inhibitor structure shows a similar loop movement but in this case the shift is only ∼4 Å.

### Molecular Docking

While our crystal structures indicate that all three NSAID scaffolds engage the oxyanion site through their carboxylic groups, we wished to determine if this result could be predicted by molecular docking using either the indomethacin or flufenamic acid bound structure (PDB codes 1S2A and 1S2C respectively). Here, the entire active site cavity including binding pockets SP1, SP2 and SP3 were used to challenge Goldscore driven docking simulations and the results are presented in Table S12.

When indomethacin was docked into its native structure (PDB 1S2A), the pose top ranked by Goldscore was within 1 Å RMSD of the pH 6.0 binding mode described herein, and also published elsewhere [26]. This was not the case when either the separation between poses was increased to 2 Å or more, or the non-native, flufenanic acid structure was used (PDB 1S2C). Instead, poses that did not show agreement across the carboxylic acid and aromatic centres with the pH 6.0 or pH 7.5 binding modes were top ranked. A pose related to that determined at pH 7.5 (RMSD 1.51 Å) was only sampled in the native structure when “diverse solutions” was increased to 3 Å RMSD. In the flufenamic acid structure, a pose that interacted with the oxyanion hole and had some overlap with the pH 7.5 binding mode (RMSD >3.5) was sampled when “diverse_solutions” was increased to an RMSD difference of 2 Å or more. Here, the high RMSD appears to be due to the incorrect position of the central indole group.

**Figure 10:**
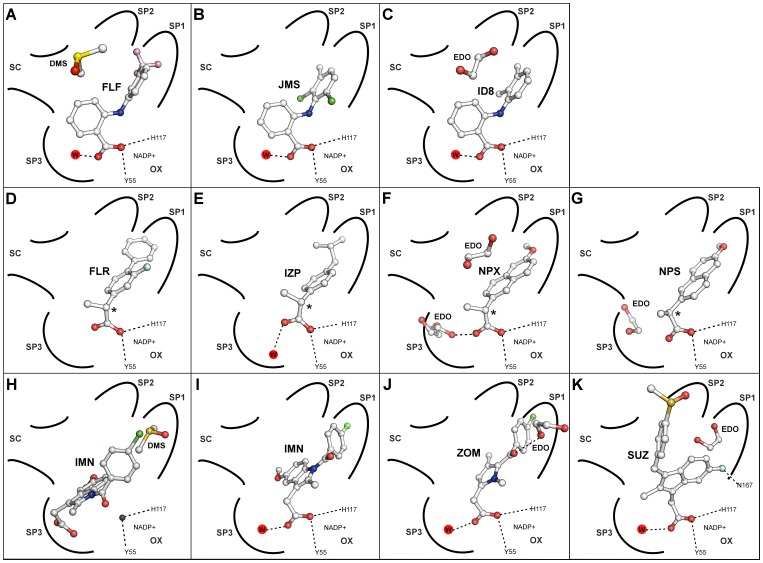
Simplified active site diagrams for eleven NSAID ternary complexes with AKR1C3. Adapted from Byrns *et al.*, 2011 [32]. Binding modes are shown for **A.** Flufenamic acid (FLF); **B.** Meclofenamic acid (JMS); **C.** Mefenamic acid (ID8); **D.** (*R*)-flurbiprofen (FLR); **E.** (*R*)-ibuprofen (IZP); **F.** (*R*)*-*naproxen (NPX); **G.** (*S*)*-*naproxen (NPS); **H.** Indomethacin pH 6.0 (IMN); **I.** Indomethacin pH 7.5; **J.** Zomepirac (ZOM); **K.** Sulindac (SUZ). Sub-pockets 1–3 are labelled SP1–3; the oxyanion site is labelled OX; the steroid channel is labelled SC; drug, water and solvent molecules are shown as ball and stick models, and selected hydrogen bonds as dashed lines.

The *N*-phenylanthranilic acid compound, flufenamic acid, was more difficult to dock correctly in both the native (PDB 1S2C) and non-native structures (PDB 1S2A). The top ranked poses retrieved with the native structure did not agree with our structure data across the carboxylic acid and both aromatic cores, although poses within 2 Å RMSD of the correct binding mode were sampled when the RMSD between poses was increased to 2 Å or more. Under the same conditions, top ranked poses within 3 Å of the correct mode were retrieved with the non-native structure. The other *N*-phenylanthranilic acids also had results consistent with flufenamic acid. Top ranked poses for meclofenamic and mefenamic acid when docked into the flufenamic acid structure showed either no agreement between the carboxylic acid group and both aromatic centres for the predicted and actual binding mode, or had RMSD values over 2 Å for those that did. Interestingly, poses that better sampled the native binding mode (RMSD less than 1 Å) for both compounds were ranked lower. Only mefenamic acid had the best pose as the top ranked solution when the RMSD separation between poses was increased to 3 Å.

Binding modes for the arylpropionic acids (flurbiprofen, ibuprofen and naproxen) were best predicted with the non-native flufenamic acid structure, where top ranked poses within 1 Å RMSD of the crystal structure were retrieved for flurbiprofen, ibuprofen and the (*S*)-naproxen enantiomer; the *R* form had an RMSD of 2.27 Å. When the non-native indomethacin structure was used, only (*R*)-naproxen had a top ranked pose within 2 Å of the correct binding mode. Like the *N*-phenylanthranilic acids, docking the arylproprionic acids under some conditions, gave lower ranked poses more similar to the actual binding modes, as indicated by lower RMSD values.

**Figure 11:**
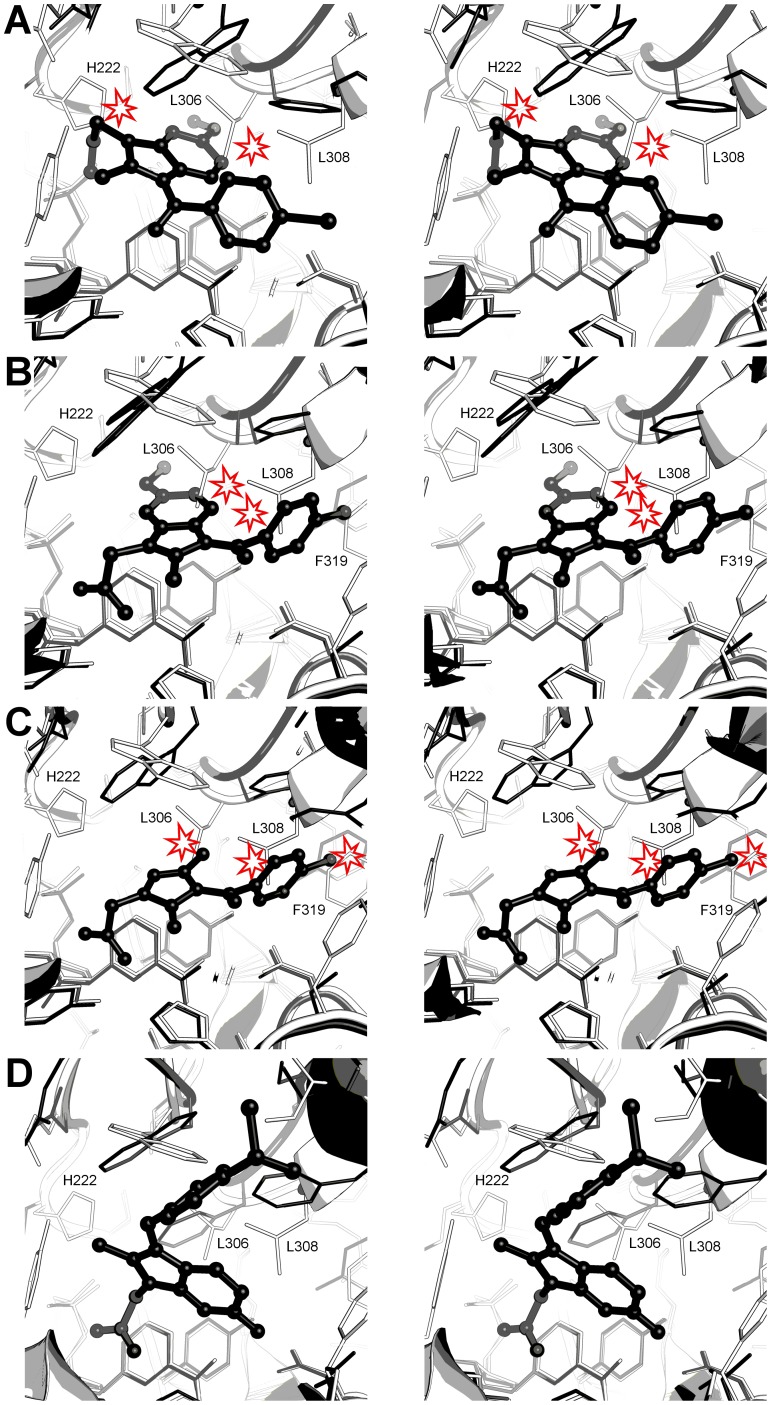
Comparison of AKR1C1, 1C2, and 1C3 active sites and indomethacin or sulindac binding modes. **A.** The indomethancin, pH 6.0 binding mode in AKR1C3 (black) and AKR1C1/2 (white) active sites. Clashes between the inhibitor and AKR1C1/2 are indicated. **B.** The indomethancin, pH 7.5 binding mode in AKR1C3 (black) and AKR1C1/2 (white) active sites. Clashes between the inhibitor and AKR1C1/2 are indicated. **C.** The zomepirac binding mode in AKR1C3 (black) and AKR1C1/2 (white) active sites. Clashes between the inhibitor and AKR1C1/2 are indicated. **D.** Sulindac binding in AKR1C3 (black) and AKR1C1/2 (white) active sites. In each figure the numbering corresponds to the AKR1C2 amino acid sequence.

The NSAIDs zomepirac and sulindac were arguably the most difficult cases for docking. The structure data presented here shows that zomepirac has a similar binding mode to indomethacin at pH 7.5, and as the latter was difficult to sample and score highly it is not surprising that zomepirac was also a challenge in both the indomethacin and flufenamic acid structures. Interestingly, few if any poses sampled in the indomethacin structure were in broad agreement with crystal structure data presented here, while that of flufenamic acid retrieved and top ranked a relevant pose (albeit with an RMSD of approximately 3 Å) when “diverse solutions” was set at 2 Å. Sulindac has a distinct binding mode, engaging both the oxyanion site and the SP2 site with the other subpockets unfilled. This new NSAID binding mode was poorly predicted with both the indomethacin and flufenamic acid structures. Although some modes predicted to engage the oxyanion hole were sampled, these incorrectly positioned the benzyl-sulfinyl tail, burying it in the SP1 binding pocket. Here, like the other scaffolds characterised in this study, the top ranked pose was not necessarily the most similar to that actual binding mode.

An RMSD cutoff of 2 Å between the top ranked docking solution and the binding mode observed in the crystal structure is used to describe docking performance [55]. Using this criterion we find overall that our docking is not predictive of crystal structure poses. Conformational differences in the large multi-cavity AKR1C3 active site clearly have an effect on docking performance, with the flufenamic structure unsurprisingly better for predicting the correct binding modes for compounds that engage the oxyanion binding site. Moreover, the best predictions were for those compounds that bind further into the SP1 pocket. This data suggests that to better sample the diverse range of compounds that bind the AKR1C3 active site using computer-based drug discovery methods, will need to account for any conformational differences. Our crystal structures provide structural data that can be used for this purpose.

### Discussion and Implications for Drug Discovery Targeting AKR1C3

The AKR1C1, 1C2 and 1C3 proteins share a high degree of similarity in sequence and structure; using the current set of crystal structures and the literature what features of each drug and protein can we target to influence isoform selectivity?

Relative to AKR1C3, the active sites of the 1C1 and 1C2 isoforms display a protrusion of four residues into the SP1 pocket, Phe-118, Leu-306, Leu-308, and Phe-319, rendering the AKR1C1/2 SP1 pocket less polar, smaller and more restrictive than the 1C3 equivalent and less able to accommodate the inhibitors discussed above. The 1C1 and 1C2 Leu-306 side chain is also inherently less mobile than the 1C3 Tyr-306 side chain which, as well illustrated in our set of crystal structures, can sample various conformations in response to ligand binding. An additional active site protrusion in AKR1C1 and 1C2 is His-222 - the site in our (*R*)-naproxen structure occupied by an ethylene glycol molecule. Structural comparison of the inhibitor structures across the isoforms may indicate how isoform specificity may be achieved.

#### *N*-phenylanthranilic acids and analogues

The crystal structures described here provide details of the binding modes of the *N*-phenylanthranilic acids (Figure 10A–C) but are these NSAID templates suitable for further discovery of AKR1C3-selective drugs? A recent and comprehensive study by Adeniji *et al.* in 2012, describes SAR experiments based on flufenamic acid derivatives and demonstrates that strong AKR1C3 binding and selectivity is attained by electronic effects [56] and by effective filling of the SP1 pocket [29]. Improved selectivity may arise from the smaller and more restrictive SP1 pocket in AKR1C2 and 1C2 isoforms less able to accommodate these inhibitors.

#### Arylproprionic acids

Binding both the SP1 pocket and oxyanion site may in part improve selectivity for AKR1C3 over the 1C2 isoform for *N*-phenylanthranilic acid analogues [29], [56], and new crystal structure data for arylpropionic acids bound to AKR1C3 show a similar binding mode (Figure 10D–G). Superimposition onto AKR1C1 and 1C2 structures suggests the compounds would not fit in the 1C1 and 1C2 SP1 pockets in a similar binding mode, yet intriguingly, all three compounds preferentially block AKR1C2 over AKR1C3 [32]. This indicates that the AKR1C2 SP1 pocket might adjust to accommodate this ligand, or perhaps more likely, that the ligand may adopt an alternative binding mode. Furthermore, electronic effects like those proposed to be involved in *N*-phenylanthranilic acid ligand binding may have a larger effect than steric effects [56]. Structural data showing the inhibition of the other isoforms is required to assess the potential of this scaffold for future drug discovery and is beyond the scope of the current study.

#### Indomethacin analogues

Indomethacin and related compounds show the greatest deviation in their binding modes (Figure 10H–K). Zomepirac and indomethacin at pH 7.5 bind like most other NSAIDs using the oxyanion site and SP1 pocket. However, indomethacin at pH 6.0 and sulindac molecules bind in the rarely occupied SP3 and SP2 pockets respectively. Their binding modes help explain the pattern of selectivity of these compounds for the AKR family members 1C1, 1C2, and 1C3, particularly indomethacin, which is selective for 1C3, and sulindac, which shows little or no selectivity [32]. Superimposition of these AKR1C3 structures on to AKR1C1 and 1C2 indicate that the smaller active sites of the latter two molecules have a profound effect on the binding of each indomethacin analogue. In the indomethacin binding mode at pH 6.0, the AKR 1C1 or 1C2 side chains His-222, Leu-306 (in the same location as the flexible AKR1C3 residue Phe-306) and Phe-118 would interfere sterically with inhibitor carboxylic acid, indole ring and *p*-chlorobenzoyl units (Figure 11A). In the pH 7.5 binding mode, overlay of our crystal structure on to 1C2 suggests the indomethacin indole ring would be sterically hindered by Leu-306, the *p*-chlorobenzoyl ring by Leu-308, and the chloride atom by Phe-319 (Figure 11B). Superimposition of zomepirac bound AKR1C3 onto AKR1C1 and 1C2 also indicates steric hindrance by the SP1 pocket residues Leu-306, Leu-308 and Phe-319 (Figure 11C). This is not consistent with the reported IC_50_ values of >50, 23, and 40 uM for 1C1, 1C2, and 1C3 isoforms respectively, that show this compound as a weak non-selective inhibitor [32] and suggest dissimilar binding modes for the different AKR isoforms. It also implies that a larger central ring may play a role in the isoform selectivity seen with indomethacin when in the pH 6.0 binding mode. In contrast to indomethacin and zomepirac, the binding path of sulindac from oxyanion site to the SP2 pocket through all three enzymes, AKR1C1, 1C2 and 1C3, is unobstructed within the active site and aided by the observed flexibility of the loop that defines the top of the AKR1C3 SP2 pocket (Figure 11D). This binding mode clearly explains the lack of isoform specificity reported for this ligand [32]. The different binding modes for indomethacin alone suggests that any novel inhibitor, for example, the *N*-(4-chlorobenzoyl)-melatonin (CBM) molecule [34] needs to be interrogated by structure activity relationships against all the indomethacin analogue crystal structures we have described.

## Supporting Information

Figure S1 Michaelis–Menten plot for AKR1C3 activity with substrate 9,10-phenanthrenequinone (inset).
(TIF)

Figure S2 **A.** Electron density maps (2*F*o-*F*c omit map; 1.0 sigma level) covering meclofenamic acid and surrounding active site residues. Figure drawn using Pymol v1.3 incentive (Schrödinger, LLC). **B.** Electron density maps (2*F*o-*F*c omit map; 1.0 sigma level) covering mefenamic acid and surrounding active site residues. Figure drawn using Pymol v1.3 incentive (Schrödinger, LLC). **C.** Electron density maps (2*F*o-*F*c omit map; 1.0 sigma level) covering (*R*)-flurbiprofen and surrounding active site residues. Figure drawn using Pymol v1.3 incentive (Schrödinger, LLC). **D.** Electron density maps (2*F*o-*F*c omit map; 1.0 sigma level) covering (*R*)-ibuprofen and surrounding active site residues. Figure drawn using Pymol v1.3 incentive (Schrödinger, LLC). **E.** Electron density maps (2*F*o-*F*c omit map; 1.0 sigma level) covering (*R*)-naproxen and surrounding active site residues. Figure drawn using Pymol v1.3 incentive (Schrödinger, LLC). **F.** Electron density maps (2*F*o-*F*c omit map; 1.0 sigma level) covering indomethacin pH 7.5 and surrounding active site residues. Figure drawn using Pymol v1.3 incentive (Schrödinger, LLC). **G.** Electron density maps (2*F*o-*F*c omit map; 1.0 sigma level) covering zomepirac and surrounding active site residues. Figure drawn using Pymol v1.3 incentive (Schrödinger, LLC). **H.** Electron density maps (2*F*o-*F*c omit map; 1.0 sigma level) covering sulindac and surrounding active site residues. Figure drawn using Pymol v1.3 incentive (Schrödinger, LLC). **I.** Electron density maps (2*F*o-*F*c omit map; 1.0 sigma level) covering (*S*)-naproxen and surrounding active site residues. Figure drawn using Pymol v1.3 incentive (Schrödinger, LLC).
(TIF)

Figure S3 Ligplot diagram of protein-ligand contacts in the meclofenamic acid structure.
(TIF)

Figure S4 Ligplot diagram of protein-ligand contacts in the mefenamic acid structure.
(TIF)

Figure S5 Ligplot diagram of protein-ligand contacts in the (*R*)-flurbiprofen structure.
(TIF)

Figure S6 Ligplot diagram of protein-ligand contacts in the (*R*)-ibuprofen structure.
(TIF)

Figure S7 Ligplot diagram of protein-ligand contacts in the (*R*)-naproxen structure.
(TIF)

Figure S8 Ligplot diagram of protein-ligand contacts in the indomethacin pH 7.5 structure.
(TIF)

Figure S9 Ligplot diagram of protein-ligand contacts in the zomepirac structure.
(TIF)

Figure S10 Ligplot diagram of protein-ligand contacts in the sulindac structure.
(TIF)

Figure S11 Ligplot diagram of protein-ligand contacts in the (*S*)-naproxen structure.
(TIF)

Table S1 Crystal properties, data collection and refinement statistics.
(PDF)

Table S2 Complementarity values for meclofenamic acid in PDB entry 3R6I and full list of atomic contacts.
(PDF)

Table S3 Complementarity values for mefenamic acid in PDB entry 3R4 and full list of atomic contacts.
(PDF)

Table S4 Complementarity values for (R)-flurbiprofen in PDB entry 3R94 and full list of atomic contacts.
(PDF)

Tables S5 Complementarity values for (R)-ibuprofen in PDB entry 3R8G and f ull list of atomic contacts.
(PDF)

Table S6 Complementarity values for (R)-naproxen in PDB entry 3UFY and full list of atomic contacts.
(PDF)

Table S7 Complementarity values for Indomethacin pH 7.5 in PDB entry 3UG8 and full list of atomic contacts.
(PDF)

Table S8 PROPKA Calculations.
(PDF)

Table S9 Complementarity values for zomepirac in PDB entry 3R8H and full list of atomic contacts.
(PDF)

Table S10 Complementarity values for sulindac in PDB entry 3R7M and full list of atomic contacts.
(PDF)

Table S11 Complementarity values for (S)-naproxen in PDB entry 3R58 and full list of atomic contacts.
(PDF)

Table S12 Comparison of predicted and actual binding poses for NSAIDS docked into Indomethacin or flufenamic acid bound AKR1C3.
(PDF)
