# Supplementary figures and images for: Crystal Structures of Three Classes of Non-Steroidal Anti-Inflammatory Drugs in Complex with Aldo-Keto Reductase 1C3
Source: PLoS One. 2012 Aug 28;7(8):e43965. doi: 10.1371/journal.pone.0043965 (PMC3429426; doi:10.1371/journal.pone.0043965)

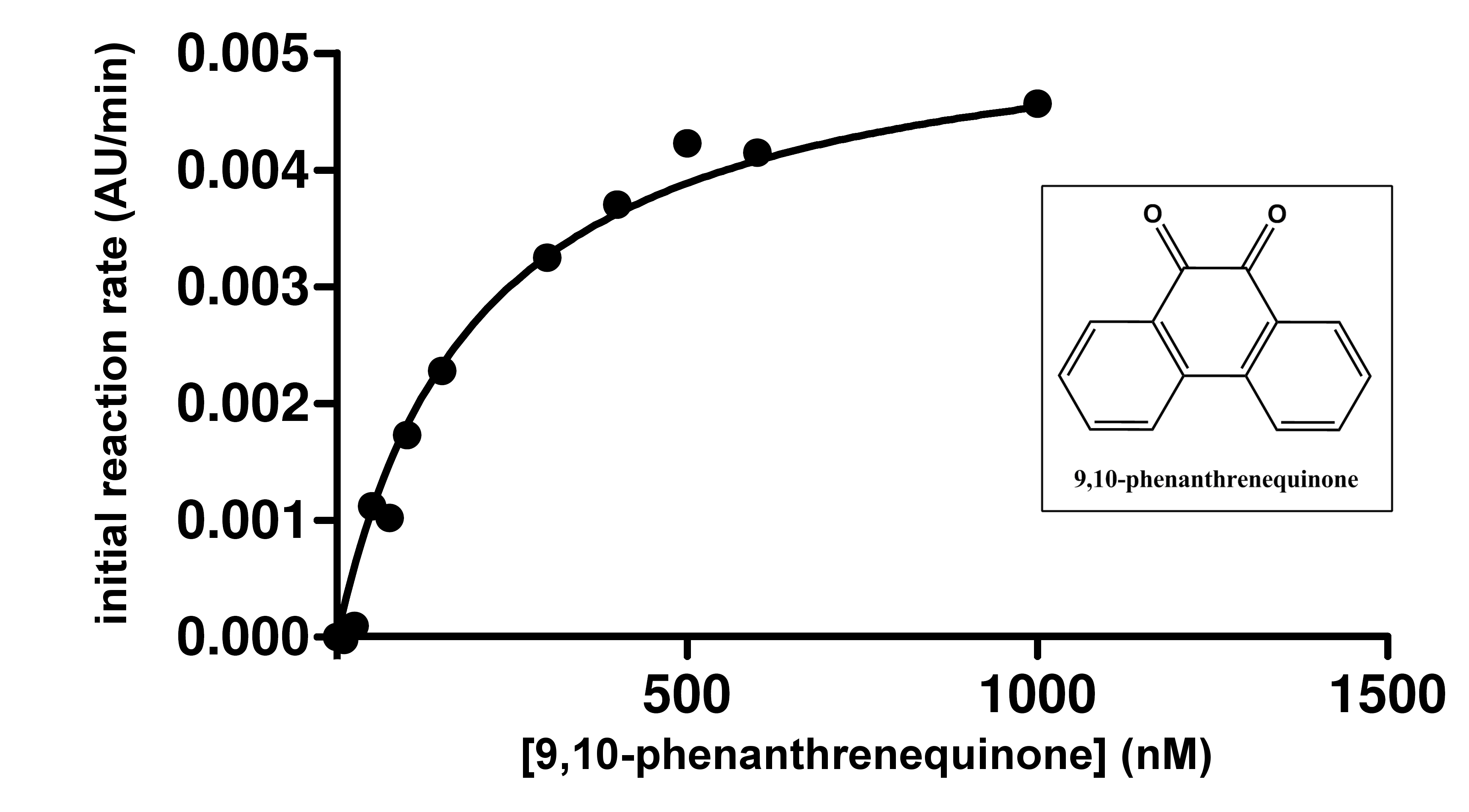

Supplement: Figure S1 — Michaelis–Menten plot for AKR1C3 activity with substrate 9,10-phenanthrenequinone (inset). (TIF) [file pone.0043965.s001.tif]

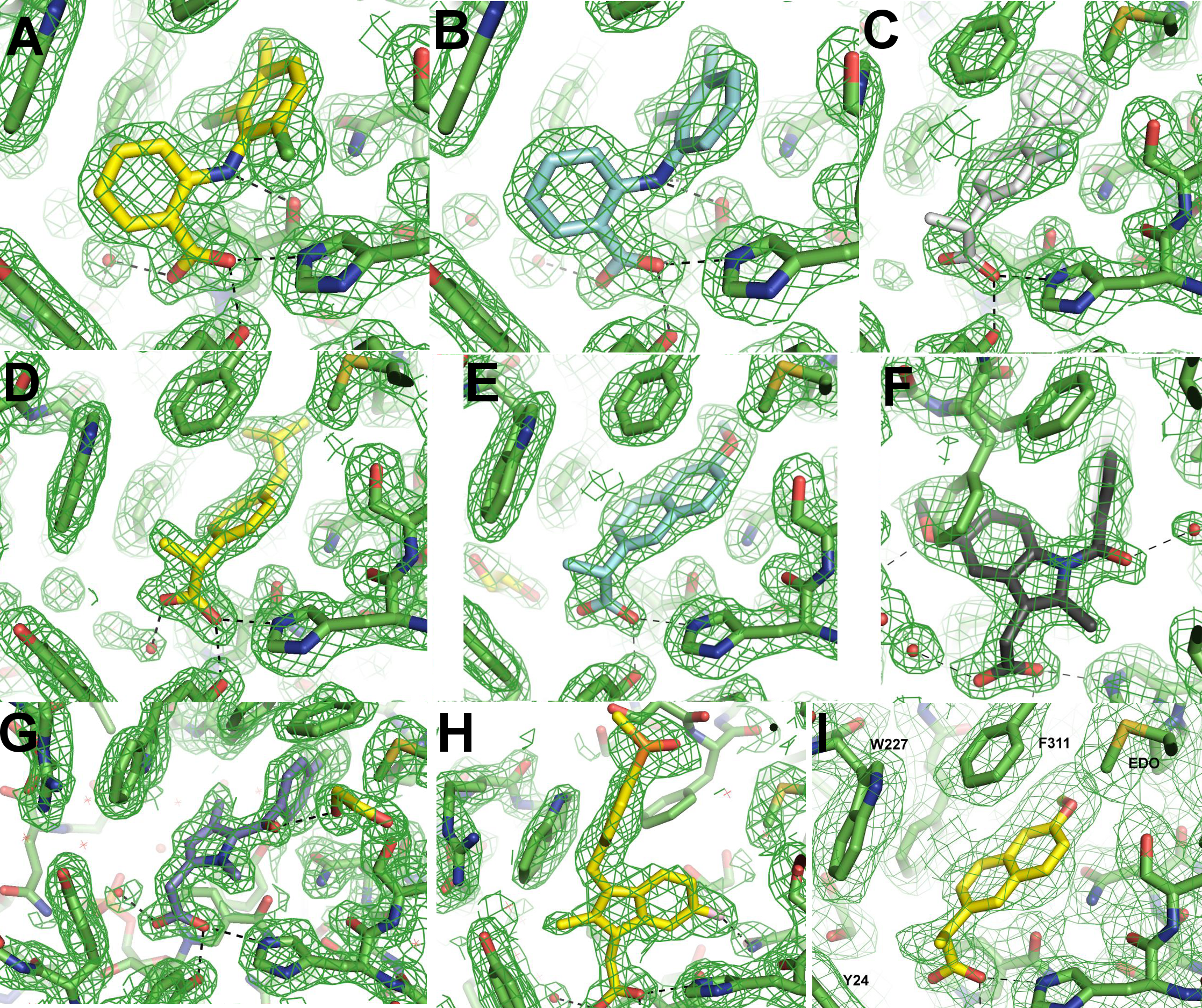

Supplement: Figure S2 — A. Electron density maps (2Fo-Fc omit map; 1.0 sigma level) covering meclofenamic acid and surrounding active site residues. Figure drawn using Pymol v1.3 incentive (Schrödinger, LLC). B. Electron density maps (2Fo-Fc omit map; 1.0 sigma level) covering mefenamic acid and surrounding active site residues. Figure drawn using Pymol v1.3 incentive (Schrödinger, LLC). C. Electron density maps (2Fo-Fc omit map; 1.0 sigma level) covering (R)-flurbiprofen and surrounding active site residues. Figure drawn using Pymol v1.3 incentive (Schrödinger, LLC). D. Electron density maps (2Fo-Fc omit map; 1.0 sigma level) covering (R)-ibuprofen and surrounding active site residues. Figure drawn using Pymol v1.3 incentive (Schrödinger, LLC). E. Electron density maps (2Fo-Fc omit map; 1.0 sigma level) covering (R)-naproxen and surrounding active site residues. Figure drawn using Pymol v1.3 incentive (Schrödinger, LLC). F. Electron density maps (2Fo-Fc omit map; 1.0 sigma level) covering indomethacin pH 7.5 and surrounding active site residues. Figure drawn using Pymol v1.3 incentive (Schrödinger, LLC). G. Electron density maps (2Fo-Fc omit map; 1.0 sigma level) covering zomepirac and surrounding active site residues. Figure drawn using Pymol v1.3 incentive (Schrödinger, LLC). H. Electron density maps (2Fo-Fc omit map; 1.0 sigma level) covering sulindac and surrounding active site residues. Figure drawn using Pymol v1.3 incentive (Schrödinger, LLC). I. Electron density maps (2Fo-Fc omit map; 1.0 sigma level) covering (S)-naproxen and surrounding active site residues. Figure drawn using Pymol v1.3 incentive (Schrödinger, LLC). (TIF) [file pone.0043965.s002.tif]

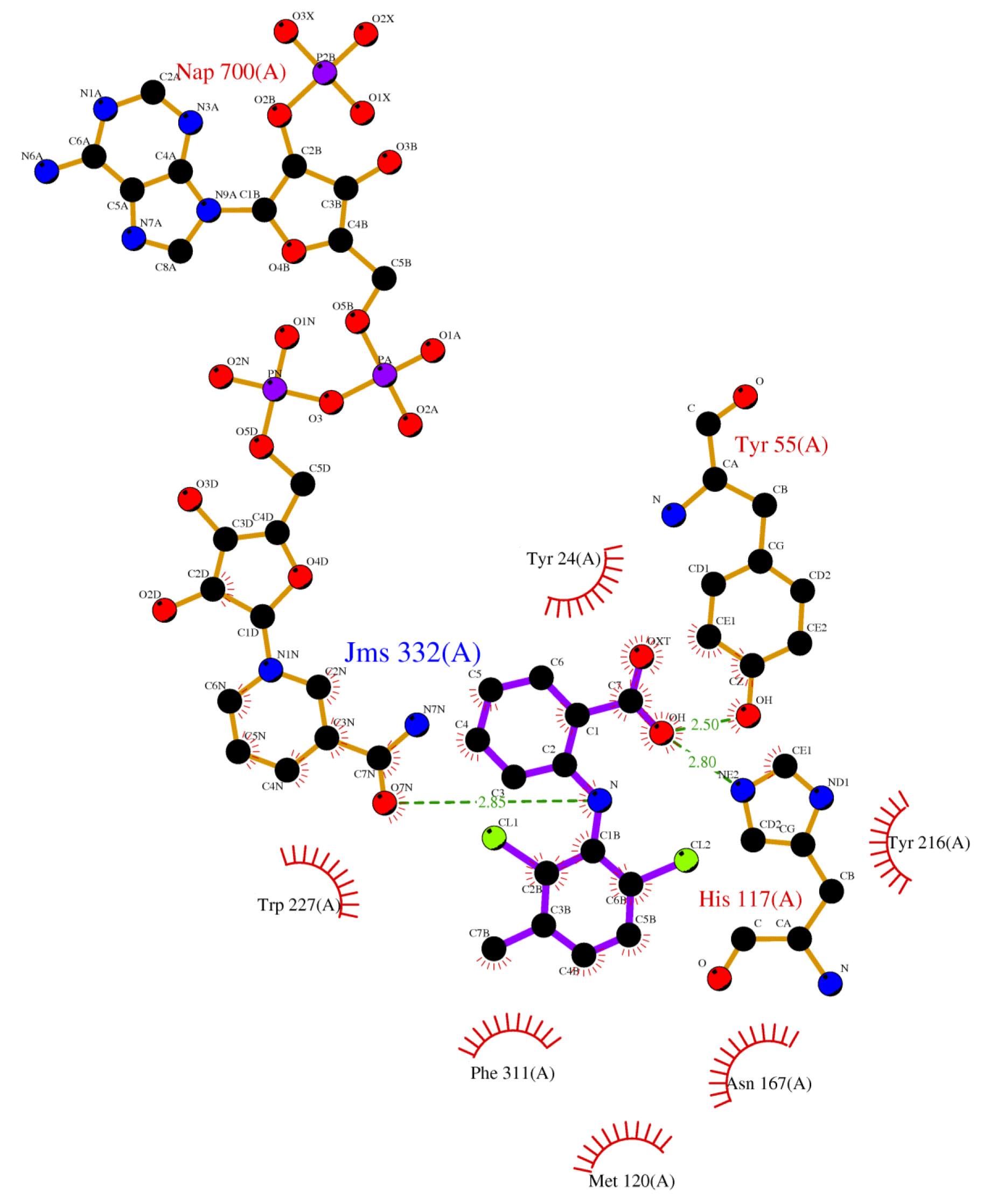

Supplement: Figure S3 — Ligplot diagram of protein-ligand contacts in the meclofenamic acid structure. (TIF) [file pone.0043965.s003.tif]

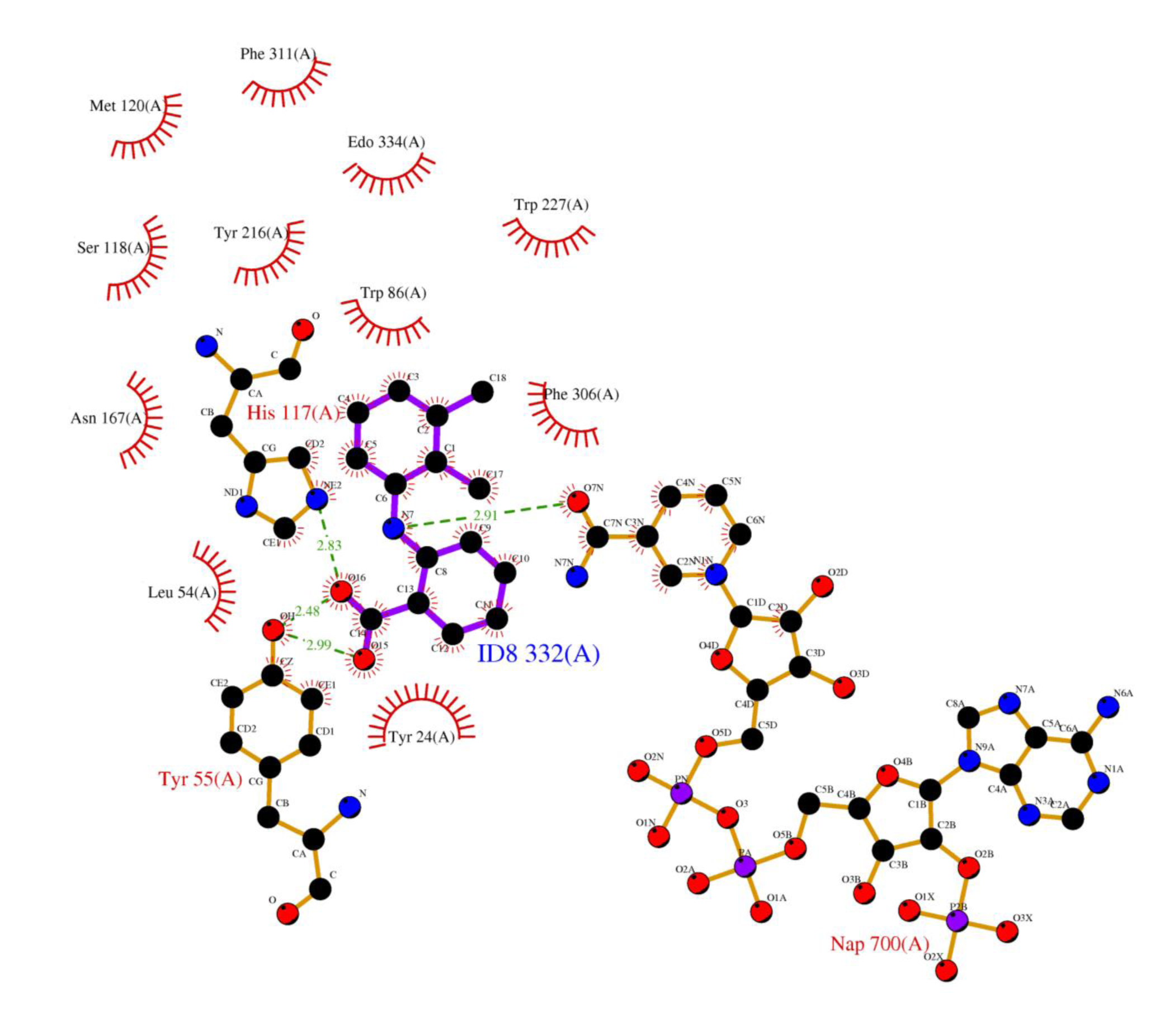

Supplement: Figure S4 — Ligplot diagram of protein-ligand contacts in the mefenamic acid structure. (TIF) [file pone.0043965.s004.tif]

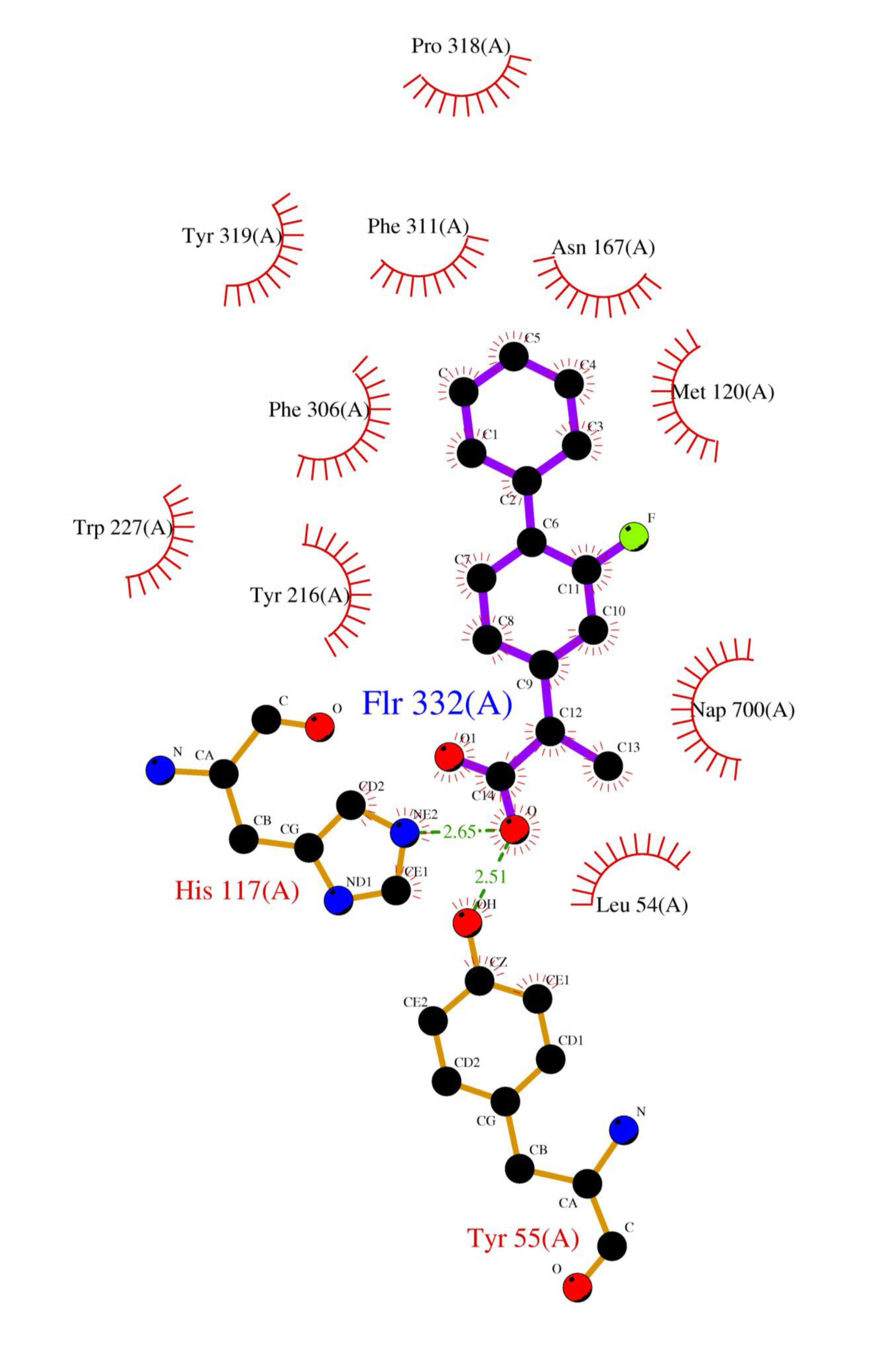

Supplement: Figure S5 — Ligplot diagram of protein-ligand contacts in the (R)-flurbiprofen structure. (TIF) [file pone.0043965.s005.tif]

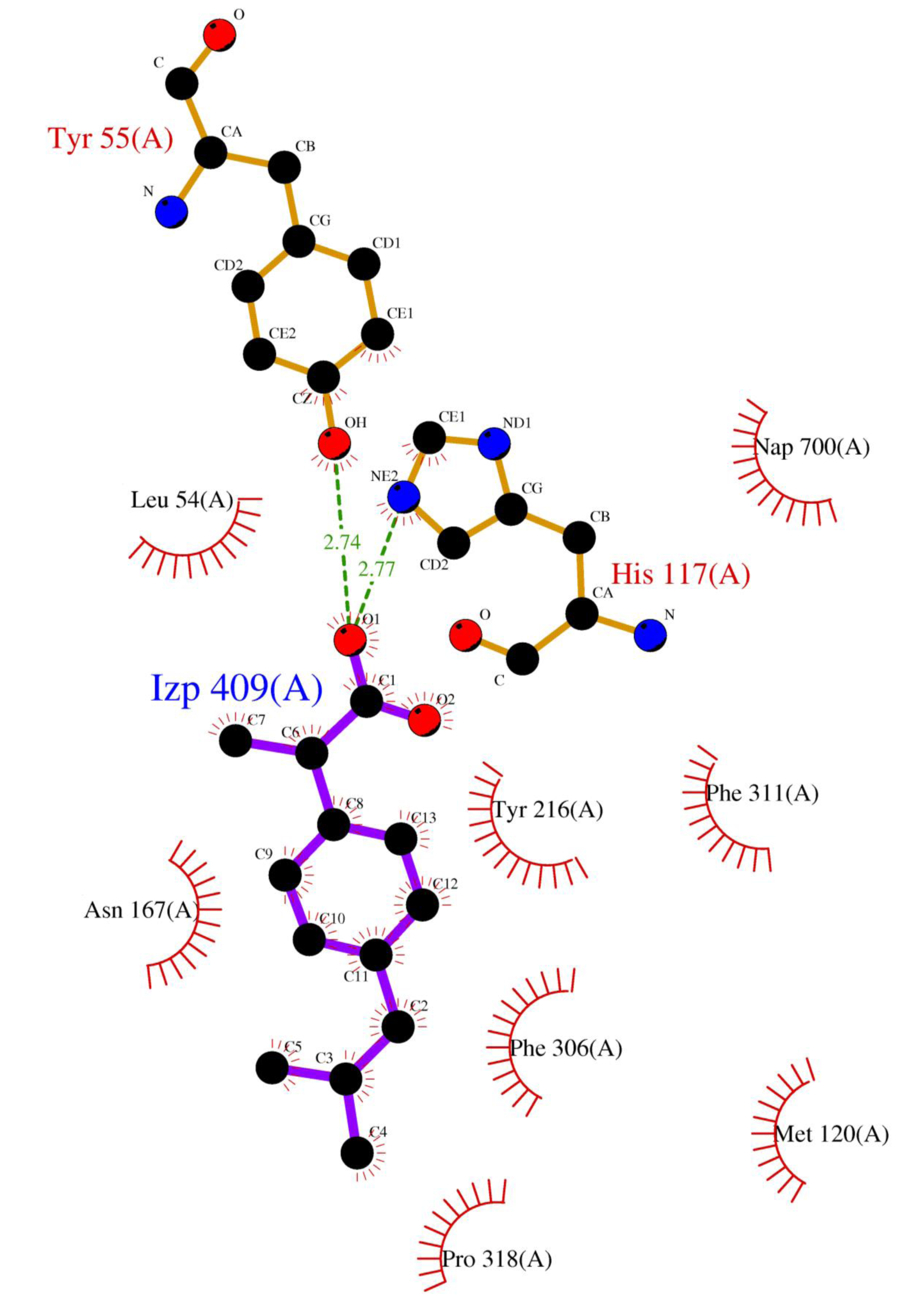

Supplement: Figure S6 — Ligplot diagram of protein-ligand contacts in the (R)-ibuprofen structure. (TIF) [file pone.0043965.s006.tif]

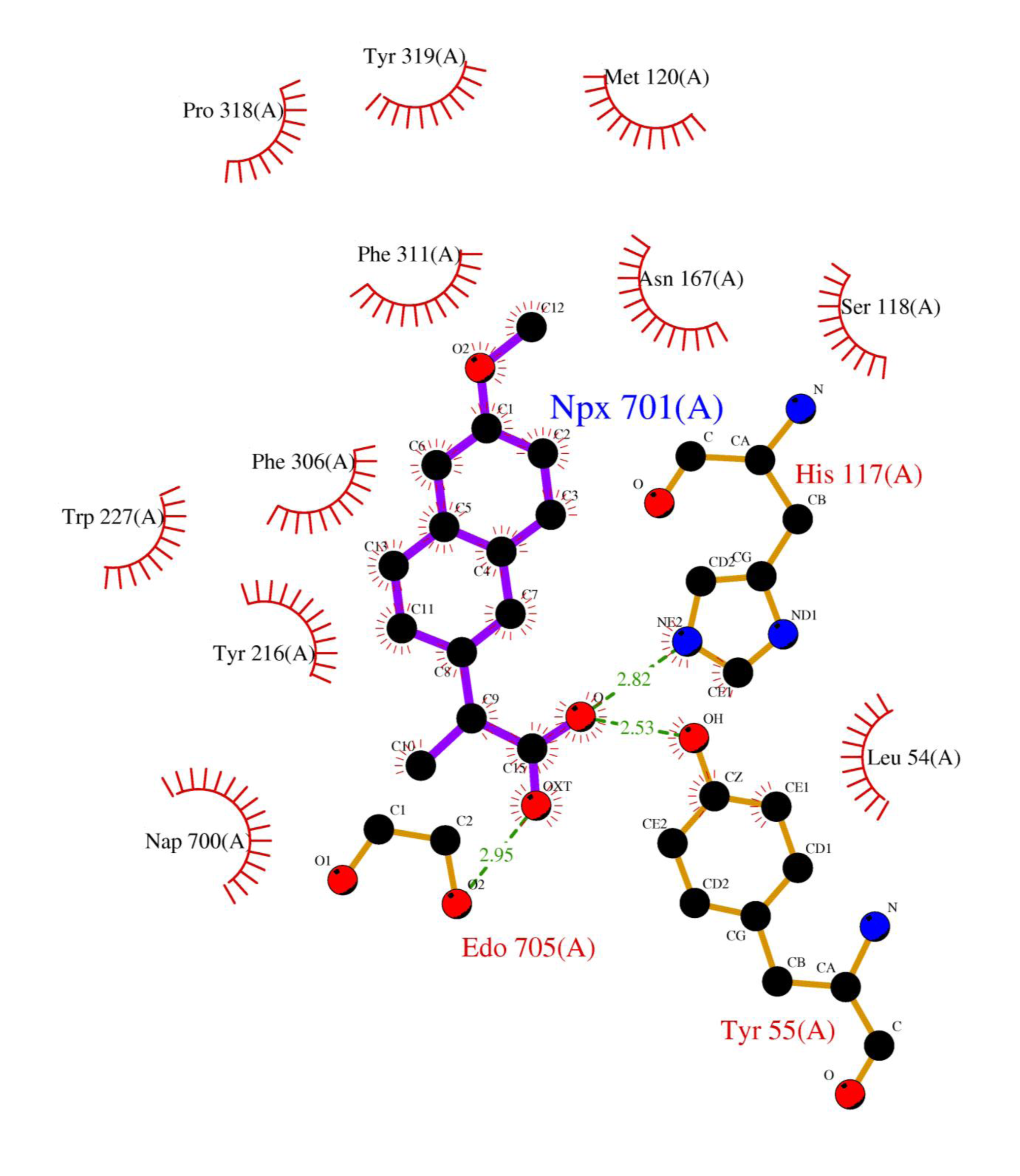

Supplement: Figure S7 — Ligplot diagram of protein-ligand contacts in the (R)-naproxen structure. (TIF) [file pone.0043965.s007.tif]

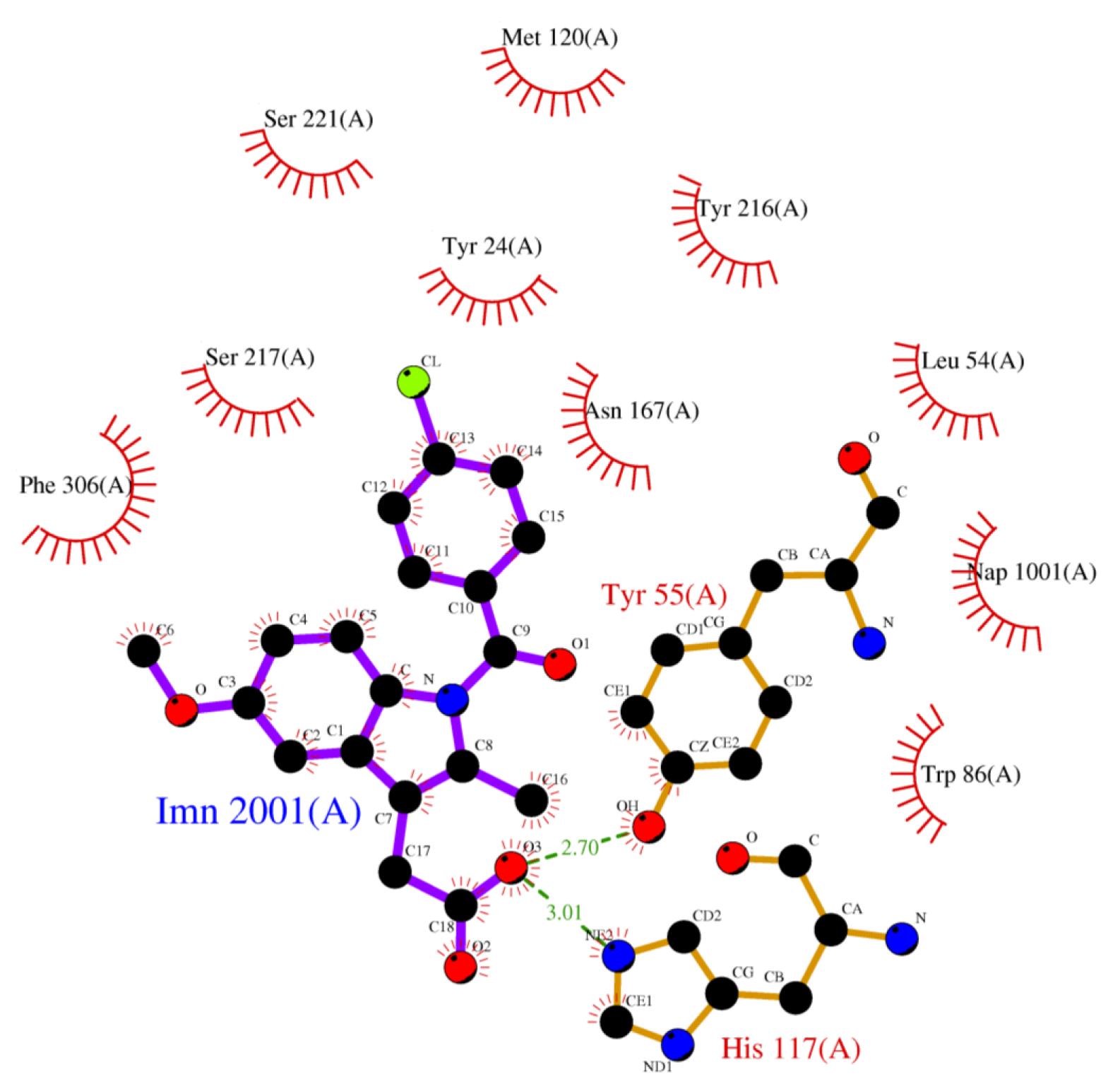

Supplement: Figure S8 — Ligplot diagram of protein-ligand contacts in the indomethacin pH 7.5 structure. (TIF) [file pone.0043965.s008.tif]

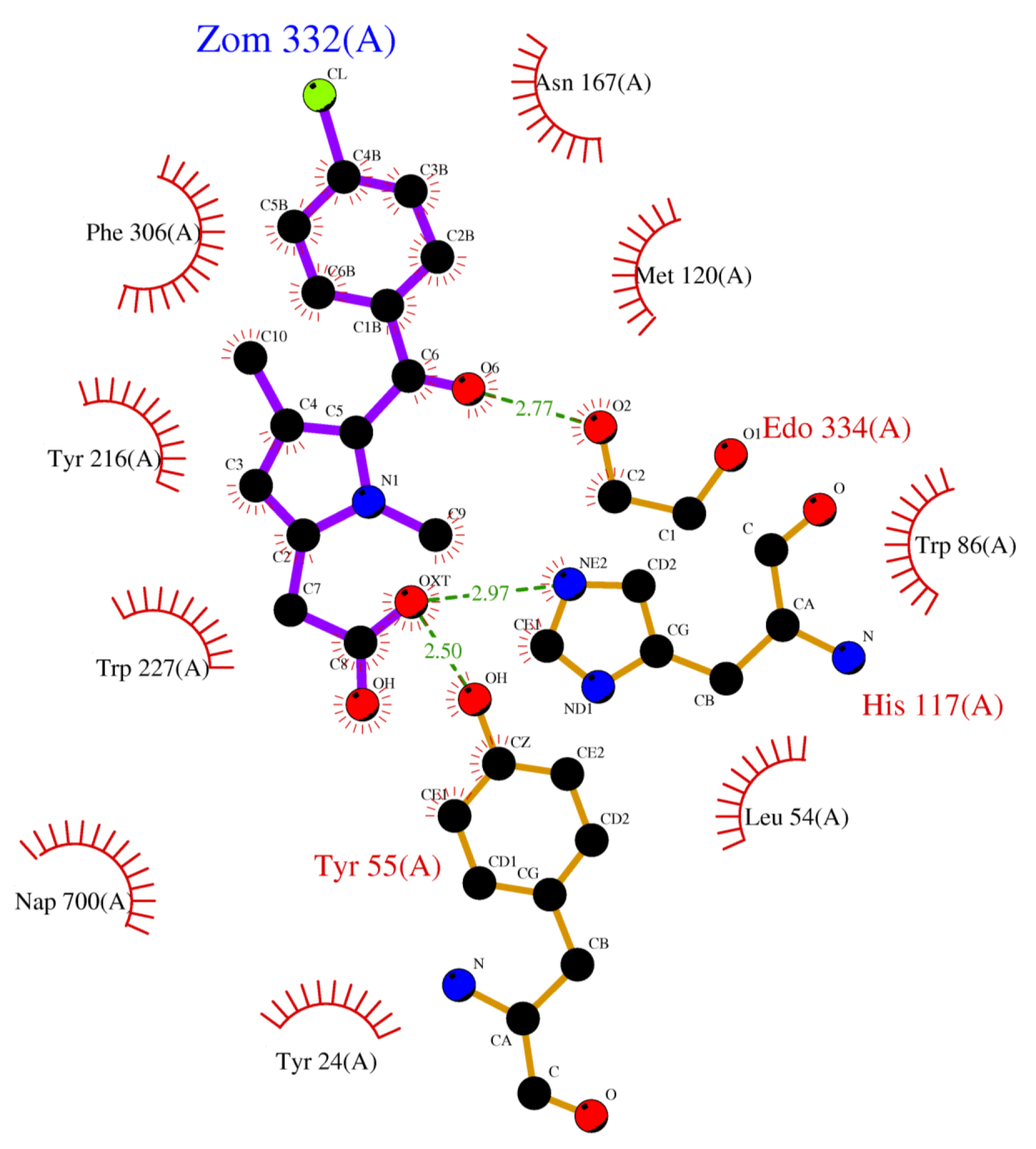

Supplement: Figure S9 — Ligplot diagram of protein-ligand contacts in the zomepirac structure. (TIF) [file pone.0043965.s009.tif]

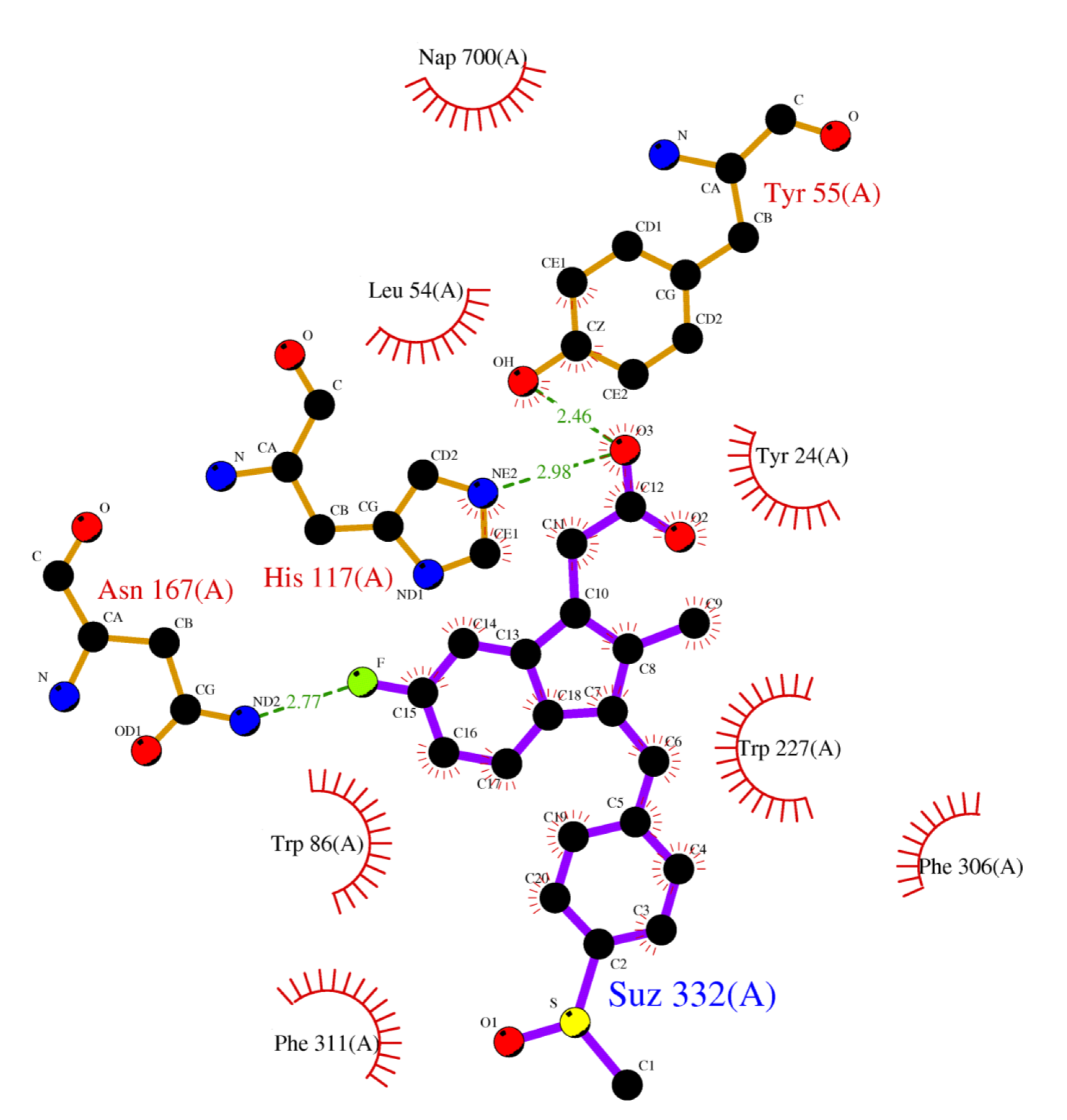

Supplement: Figure S10 — Ligplot diagram of protein-ligand contacts in the sulindac structure. (TIF) [file pone.0043965.s010.tif]

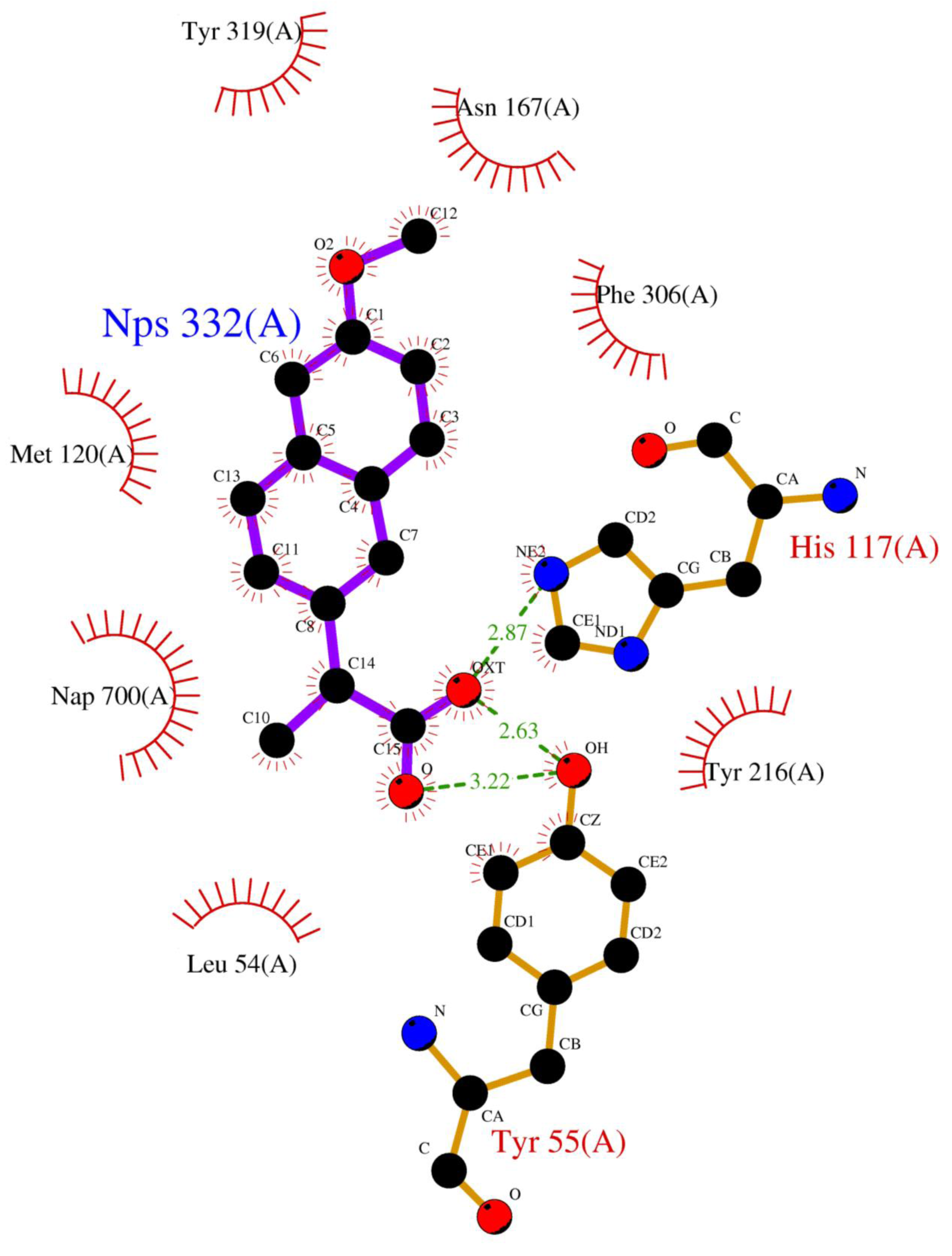

Supplement: Figure S11 — Ligplot diagram of protein-ligand contacts in the (S)-naproxen structure. (TIF) [file pone.0043965.s011.tif]
