# Supplementary material for: Crystal Structures of Three Classes of Non-Steroidal Anti-Inflammatory Drugs in Complex with Aldo-Keto Reductase 1C3
Source: PLoS One. 2012 Aug 28;7(8):e43965. doi: 10.1371/journal.pone.0043965 (PMC3429426; doi:10.1371/journal.pone.0043965)
Supplement: Table S1 — Crystal properties, data collection and refinement statistics. (PDF) [file pone.0043965.s012.pdf]

Table S1. Crystal properties, data collection and refinement statistics.

	(*S*)-naproxen	Indomethacin pH 6.8
PDB code	3R58	3UGR
**Data collection:**		
Space group	$P2_12_12_1$	$P2_12_12_1$
Unit-cell parameters (Å)		
a	58.76	55.96
b	64.93	63.29
c	96.27	96.39
Beamline	AS MX-2	AS MX-2
Resolution	2.30 (2.42-	1.65 (1.74-1.65)
Wavelength (Å)	2.30)	0.97941
R_{merge}†	0.97941	0.070 (0.623)
Completeness (%)	0.172 (0.807)	99.7 (98.2)
Observed reflections	99.8 (100.0)	1202821
$\langle I/\sigma(I)\rangle$	242202	39.3 (6.3)
Multiplicity	12.8 (3.3)	28.6 (27.8)
Wilson *B* (Å^2)	14.3 (14.6)	18.4
	37.5	
**Refinement:**		
Resolution range		48.3-1.65
Reflections used	19.8-2.30	39922
R factor	15194	0.168
R_{free}	0.180	0.203
Average *B* factor (Å^2)	0.220	18.6
R.m.s. deviation from	31.0	
ideal		0.025
Bond lengths (Å)	0.021	2.123
Bond angles (°)	1.813	
Ramachandran plot (%)		97.7
Favoured	96.0	0.0
Outliers	0.0	1-5, 125-130,
Missing density	1-5, 125-137,	320-331‡
(unmodeled)	323-331‡	

Data for the high resolution shell are shown in parentheses.† $R_{merge} = \Sigma_{hkl}\Sigma_i \mid I_i(hkl) - \langle I(hkl)\rangle \mid / \Sigma_{hkl}\Sigma_i I_i(hkl)$, where $I_i(hkl)$ is the intensity of the *i*th measurement of an equivalent reflection with indices *hkl*. ‡ Includes the C-terminal hexahistidine tag.
