# Supplementary material for: Crystal Structures of Three Classes of Non-Steroidal Anti-Inflammatory Drugs in Complex with Aldo-Keto Reductase 1C3
Source: PLoS One. 2012 Aug 28;7(8):e43965. doi: 10.1371/journal.pone.0043965 (PMC3429426; doi:10.1371/journal.pone.0043965)
Supplement: Table S2 — Complementarity values for meclofenamic acid in PDB entry 3R6I and full list of atomic contacts. (PDF) [file pone.0043965.s013.pdf]

Table S2. Complementarity values for meclofenamic acid in PDB entry 3R6I and full list of atomic contacts. Total number of contacts is 98.

Theoretical maximum (Å^2)	464
Actual value (Å^2)	426
Normalised complementarity	0.92

Ligand atom			Protein atom					
N	Name	Class	Residue		Name	Class	Dist	Surf
1	OXT	II	TYR	55A	OH	I	3.0	9.0
1	OXT	II	NAP	700A	C5N	V	3.2	13.0
1	OXT	II	NAP	700A	C6N	V	3.2	3.5
1	OXT	II	TYR	55A	CE1	V	3.2	3.3
1	OXT	II	TYR	55A	CZ	V	3.3	1.2
1	OXT	II	TYR	24A	CB	IV	3.8	6.6*
1	OXT	II	TYR	24A	CG	V	4.0	0.2
1	OXT	II	TYR	24A	CD2	V	4.4	0.3
2	C7	VI	TYR	55A	OH	I	3.1	3.8
2	C7	VI	NAP	700A	C4N	V	3.2	7.4
2	C7	VI	TYR	55A	CE1	V	3.2	4.9
3	OH	II	TYR	55A	OH	I	2.5	17.7
3	OH	II	HIS	117A	NE2	I	2.8	21.3
3	OH	II	TYR	55A	CE1	V	3.0	0.3
3	OH	II	NAP	700A	C3N	V	3.0	2.8
4	C1	V	NAP	700A	C4N	V	3.8	2.2
4	C1	V	LEU	54A	CD2	IV	4.1	3.4
4	C1	V	NAP	700A	C5N	V	4.2	0.7
4	C1	V	PHE	306A	CE1	V	4.9	0.2
5	C6	V	TYR	24A	CZ	V	4.0	13.2
5	C6	V	TYR	24A	CE2	V	4.0	3.4
5	C6	V	TYR	24A	CE1	V	4.1	3.8
5	C6	V	TYR	24A	CD2	V	4.1	0.7
5	C6	V	TYR	24A	CD1	V	4.1	0.4
5	C6	V	LEU	54A	CD2	IV	4.3	3.1
5	C6	V	PHE	306A	CE1	V	4.8	2.5
5	C6	V	NAP	700A	C5N	V	4.9	0.4
5	C6	V	PHE	306A	CZ	V	5.2	0.4
6	C5	V	TRP	227A	CZ3	V	3.6	16.6
6	C5	V	TRP	227A	CH2	V	3.6	4.0
6	C5	V	TYR	24A	CZ	V	4.0	10.1
6	C5	V	TYR	24A	CE2	V	4.1	0.9
6	C5	V	TRP	227A	CE3	V	4.2	0.2
6	C5	V	TYR	24A	CE1	V	4.3	0.7
6	C5	V	PHE	306A	CE1	V	4.4	2.9
6	C5	V	LEU	54A	CD2	IV	4.7	2.9
6	C5	V	PHE	306A	CZ	V	5.1	0.2
7	C4	V	TRP	227A	CH2	V	3.5	17.9
7	C4	V	TRP	227A	CZ3	V	3.6	3.8
7	C4	V	TRP	227A	CZ2	V	3.7	5.8
7	C4	V	TRP	227A	CE3	V	3.9	3.6
7	C4	V	PHE	306A	CE1	V	4.0	1.1
7	C4	V	TRP	227A	CE2	V	4.0	0.7
7	C4	V	LEU	54A	CD2	IV	4.7	2.5
7	C4	V	PHE	311A	CZ	V	4.8	0.2
8	C3	V	PHE	306A	CE1	V	4.0	1.6
8	C3	V	PHE	311A	CZ	V	4.3	8.3
8	C3	V	LEU	54A	CD2	IV	4.5	2.2

8	C3	V	PHE	311A	CE1	V	5.0	0.2
9	C2	V	LEU	54A	CD2	IV	4.1	1.1
10	N	I	NAP	700A	O7N	II	2.9	11.3
11	C1B	V	NAP	700A	O7N	II	3.1	3.4
12	C2B	V	NAP	700A	O7N	II	3.7	2.5
12	C2B	V	TYR	216A	OH	I	3.7	1.1
12	C2B	V	PHE	311A	CZ	V	4.2	0.9
13	CL1	IV	PHE	306A	CG	V	3.2	30.9
13	CL1	IV	PHE	306A	CD1	V	3.2	5.5
13	CL1	IV	TYR	216A	OH	I	3.4	13.1*
13	CL1	IV	PHE	306A	CD2	V	3.5	3.1
13	CL1	IV	PHE	306A	CE1	V	3.5	1.4
13	CL1	IV	NAP	700A	C4N	V	3.7	5.9
14	C3B	V	PHE	311A	CZ	V	3.9	2.9
14	C3B	V	TYR	216A	OH	I	4.0	2.5
14	C3B	V	ASN	167A	OD1	II	4.0	0.9
15	C7B	IV	PHE	311A	CE2	V	3.9	15.3
15	C7B	IV	PHE	311A	CD2	V	3.9	5.4
15	C7B	IV	TYR	216A	OH	I	4.0	8.3*
15	C7B	IV	ASN	167A	OD1	II	4.1	3.4*
15	C7B	IV	PHE	306A	CB	IV	4.2	7.9
15	C7B	IV	PHE	311A	CG	V	4.3	1.1
15	C7B	IV	TYR	319A	CE2	V	4.5	17.9
15	C7B	IV	ASN	167A	CG	VI	4.6	0.4
15	C7B	IV	TYR	319A	OH	I	4.6	0.4*
15	C7B	IV	MET	120A	CE	IV	4.8	2.0
15	C7B	IV	PHE	311A	CB	IV	5.1	0.7
15	C7B	IV	PRO	318A	CG	IV	5.4	3.6
15	C7B	IV	TYR	317A	CE1	V	5.7	0.4
16	C4B	V	MET	120A	CE	IV	3.6	18.6
16	C4B	V	ASN	167A	ND2	III	3.9	5.4
16	C4B	V	PHE	311A	CE1	V	3.9	4.3
16	C4B	V	ASN	167A	CG	VI	4.0	1.8
16	C4B	V	ASN	167A	CB	IV	4.7	0.2
17	C5B	V	ASN	167A	ND2	III	3.7	6.1
17	C5B	V	SER	118A	CB	VI	3.9	13.2
17	C5B	V	MET	120A	CE	IV	4.0	4.5
17	C5B	V	SER	118A	OG	I	4.0	0.2
17	C5B	V	PHE	311A	CE1	V	4.1	6.1
17	C5B	V	TRP	86A	CH2	V	5.2	1.8
18	C6B	V	NAP	700A	O7N	II	3.5	3.8
18	C6B	V	ASN	167A	ND2	III	3.7	0.7
18	C6B	V	PHE	311A	CE1	V	4.6	1.6
19	CL2	IV	HIS	117A	CD2	V	3.3	23.5
19	CL2	IV	TRP	86A	CZ3	V	3.3	24.7
19	CL2	IV	HIS	117A	NE2	I	3.4	1.0*
19	CL2	IV	TRP	86A	CH2	V	3.8	2.7
19	CL2	IV	LEU	54A	CD2	IV	4.0	1.0
19	CL2	IV	HIS	117A	CG	V	4.0	0.2
19	CL2	IV	SER	118A	CB	VI	4.4	0.8

Legend:
N - ligand atom number in PDB entry
Dist - distance (A) between the ligand and protein atoms
Surf - contact surface area (A**2) between the ligand and protein atoms
* - indicates destabilizing contacts

I Hydrophilic - N and O that can donate and accept hydrogen bonds (e.g., oxygen of hydroxyl group of Ser. or Thr)
II Acceptor - N or O that can only accept a hydrogen bond
III Donor - N that can only donate a hydrogen bond

IV	Hydrophobic	- Cl, Br, I and all C atoms that are not in aromatic rings and do not have a covalent bond to a N or O atom
V	Aromatic	- C in aromatic rings irrespective of any other bonds formed by the atom
VI	Neutral	- C atoms that have a covalent bond to at least one atom of class I or two or more atoms from class II or III; atoms; S, F, P, and metal atoms in all cases
VII	Neutral-donor	- C atoms that have a covalent bond with only one atom of class III
VIII	Neutral-acceptor	- C atoms that have a covalent bond with only one atom of class II
