# Supplementary material for: Crystal Structures of Three Classes of Non-Steroidal Anti-Inflammatory Drugs in Complex with Aldo-Keto Reductase 1C3
Source: PLoS One. 2012 Aug 28;7(8):e43965. doi: 10.1371/journal.pone.0043965 (PMC3429426; doi:10.1371/journal.pone.0043965)
Supplement: Table S3 — Complementarity values for mefenamic acid in PDB entry 3R4 and full list of atomic contacts. (PDF) [file pone.0043965.s014.pdf]

Table S3. Complementarity values for mefenamic acid in PDB entry 3R4 and full list of atomic contacts. Total number of contacts is 91.

Theoretical maximum ($Å^2$)	448
Actual value ($Å^2$)	378
Normalised complementarity	0.84

Ligand atom			Protein atom				Dist	Surf
N	Name	Class	Residue		Name	Class		
1	O16	II	TYR	55A	OH	I	2.5	17.0
1	O16	II	HIS	117A	NE2	I	2.8	13.9
1	O16	II	TYR	55A	CE1	V	3.0	0.7
1	O16	II	NAP	700A	C3N	V	3.0	3.1
2	C14	VI	TYR	55A	OH	I	3.1	4.3
2	C14	VI	NAP	700A	C4N	V	3.2	9.9
2	C14	VI	TYR	55A	CE1	V	3.2	3.6
3	O15	II	TYR	55A	OH	I	3.0	9.0
3	O15	II	NAP	700A	C6N	V	3.1	16.1
3	O15	II	TYR	55A	CE1	V	3.2	3.8
3	O15	II	TYR	55A	CZ	V	3.3	1.0
3	O15	II	NAP	700A	C2D	VI	3.7	2.9
3	O15	II	TYR	24A	CB	IV	3.7	4.9*
3	O15	II	TYR	24A	CD2	V	4.4	0.3
4	C13	V	NAP	700A	C4N	V	3.9	2.0
4	C13	V	LEU	54A	CD2	IV	3.9	2.9
4	C13	V	NAP	700A	C5N	V	4.1	0.7
4	C13	V	PHE	306A	CE1	V	4.5	0.4
5	C12	V	TYR	24A	CD2	V	3.9	16.6
5	C12	V	TYR	24A	CE2	V	3.9	0.2
5	C12	V	TYR	24A	CG	V	4.0	2.0
5	C12	V	TYR	24A	CZ	V	4.0	0.9
5	C12	V	TYR	24A	CD1	V	4.1	1.8
5	C12	V	TYR	24A	CE1	V	4.1	0.2
5	C12	V	LEU	54A	CD2	IV	4.3	2.2
5	C12	V	PHE	306A	CE1	V	4.6	2.5
5	C12	V	PHE	306A	CZ	V	4.7	1.8
6	C11	V	TRP	227A	CZ3	V	3.6	16.2
6	C11	V	TRP	227A	CH2	V	3.7	2.5
6	C11	V	TYR	24A	CZ	V	3.9	11.7
6	C11	V	TYR	24A	CE2	V	4.0	1.8
6	C11	V	PHE	306A	CE1	V	4.3	2.9
6	C11	V	TYR	24A	CE1	V	4.3	0.4
6	C11	V	LEU	54A	CD2	IV	4.5	2.5
7	C10	V	TRP	227A	CH2	V	3.5	19.5
7	C10	V	TRP	227A	CZ3	V	3.5	3.1
7	C10	V	TRP	227A	CZ2	V	3.8	4.9
7	C10	V	TRP	227A	CE3	V	3.8	3.6
7	C10	V	PHE	306A	CE1	V	3.9	1.3
7	C10	V	EDO	334A	C1	VI	3.9	5.6
7	C10	V	LEU	54A	CD2	IV	4.3	0.4
8	C9	V	EDO	334A	C1	VI	3.9	10.5
8	C9	V	PHE	306A	CE1	V	3.9	2.5
8	C9	V	LEU	54A	CD2	IV	4.0	0.2
8	C9	V	EDO	334A	C2	VI	4.4	2.0
8	C9	V	PHE	306A	CD1	V	4.5	0.9
8	C9	V	PHE	311A	CZ	V	4.6	1.3
9	C8	V	LEU	54A	CD2	IV	3.7	2.5
9	C8	V	PHE	306A	CE1	V	4.2	0.2

10	N7	I	NAP	700A	O7N	II	2.9	4.2
11	C6	V	NAP	700A	O7N	II	3.1	2.9
12	C1	V	NAP	700A	O7N	II	3.4	3.1
12	C1	V	ASN	167A	ND2	III	3.9	0.2
12	C1	V	PHE	311A	CZ	V	4.3	0.4
13	C17	IV	TYR	216A	OH	I	3.4	19.5*
13	C17	IV	PHE	306A	CD1	V	3.5	29.2
13	C17	IV	PHE	306A	CG	V	3.6	2.7
13	C17	IV	NAP	700A	O7N	II	3.7	2.0*
13	C17	IV	PHE	306A	CE1	V	3.7	1.3
13	C17	IV	NAP	700A	C4N	V	3.9	3.6
14	C5	V	TRP	86A	CZ3	V	3.7	17.7
14	C5	V	NAP	700A	O7N	II	3.8	3.6
14	C5	V	HIS	117A	CD2	V	3.8	3.8
14	C5	V	TRP	86A	CH2	V	3.8	1.3
14	C5	V	EDO	334A	C1	VI	4.8	0.2
15	C4	V	SER	118A	CB	VI	3.6	20.6
15	C4	V	TRP	86A	CH2	V	3.8	13.0
15	C4	V	PHE	311A	CE1	V	4.2	3.8
15	C4	V	ASN	167A	ND2	III	4.2	0.2
15	C4	V	EDO	334A	O2	I	4.6	0.2
15	C4	V	MET	120A	CE	IV	4.6	0.2
16	C3	V	MET	120A	CE	IV	3.6	19.5
16	C3	V	PHE	311A	CE1	V	3.7	5.6
16	C3	V	SER	118A	OG	I	3.9	3.4
16	C3	V	SER	118A	CB	VI	3.9	1.8
16	C3	V	ASN	167A	ND2	III	4.0	1.6
17	C2	V	ASN	167A	ND2	III	3.8	2.7
17	C2	V	PHE	311A	CE1	V	3.9	2.5
18	C18	IV	PHE	311A	CE2	V	4.1	15.5
18	C18	IV	PHE	311A	CD2	V	4.1	5.4
18	C18	IV	MET	120A	CE	IV	4.1	9.9
18	C18	IV	PHE	311A	CG	V	4.1	0.2
18	C18	IV	ASN	167A	OD1	II	4.1	11.4*
18	C18	IV	ASN	167A	CG	VI	4.2	0.9
18	C18	IV	TYR	216A	OH	I	4.3	1.6*
18	C18	IV	PHE	306A	CB	IV	4.8	4.5
18	C18	IV	PHE	311A	CB	IV	4.9	0.2
18	C18	IV	ASN	167A	CB	IV	5.0	0.7
18	C18	IV	TYR	319A	CE2	V	5.3	11.0
18	C18	IV	PRO	318A	CG	IV	5.6	2.5
18	C18	IV	TYR	317A	CE1	V	6.2	0.9

Legend:
N - ligand atom number in PDB entry
Dist - distance (A) between the ligand and protein atoms
Surf - contact surface area (A**2) between the ligand and protein atoms
* - indicates destabilizing contacts

I	Hydrophilic	- N and O that can donate and accept hydrogen bonds (e.g., oxygen of hydroxyl group of Ser. or Thr)
II	Acceptor	- N or O that can only accept a hydrogen bond
III	Donor	- N that can only donate a hydrogen bond
IV	Hydrophobic	- Cl, Br, I and all C atoms that are not in aromatic rings and do not have a covalent bond to a N or O atom
V	Aromatic	- C in aromatic rings irrespective of any other bonds formed by the atom
VI	Neutral	- C atoms that have a covalent bond to at least one

atom of class I or two or more atoms from class II or III; atoms; S, F, P, and metal atoms in all cases

VII Neutral-donor - C atoms that have a covalent bond with only one atom of class III

VIII Neutral-acceptor - C atoms that have a covalent bond with only one atom of class II
