# Supplementary material for: Crystal Structures of Three Classes of Non-Steroidal Anti-Inflammatory Drugs in Complex with Aldo-Keto Reductase 1C3
Source: PLoS One. 2012 Aug 28;7(8):e43965. doi: 10.1371/journal.pone.0043965 (PMC3429426; doi:10.1371/journal.pone.0043965)
Supplement: Table S4 — Complementarity values for (R)-flurbiprofen in PDB entry 3R94 and full list of atomic contacts. (PDF) [file pone.0043965.s015.pdf]

Table S4. Complementarity values for (*R*)-flurbiprofen in PDB entry 3R94 and full list of atomic contacts. Total number of contacts is 83.

Theoretical maximum (Å^2)	444
Actual value (Å^2)	447
Normalised complementarity	1.01

Ligand atom			Protein atom			Dist	Surf
N	Name	Class	Residue	Name	Class		
1	C1	V	PHE 306A	CB	IV	3.7	12.1
1	C1	V	PHE 311A	CD2	V	3.8	10.1
1	C1	V	PHE 311A	CG	V	3.8	0.2
1	C1	V	TYR 216A	OH	I	4.2	2.0
2	C	V	TYR 319A	CE2	V	3.7	13.5
2	C	V	PHE 311A	CG	V	3.8	13.2
2	C	V	PHE 311A	CD2	V	3.8	2.7
2	C	V	PHE 306A	CB	IV	4.0	5.4
2	C	V	PHE 311A	CB	IV	4.1	1.3
2	C	V	TYR 319A	CZ	V	4.4	0.4
2	C	V	TYR 317A	CE1	V	4.4	1.3
2	C	V	PHE 306A	O	II	4.4	1.3
3	C5	V	TYR 319A	CE2	V	3.6	19.5
3	C5	V	PRO 318A	CG	IV	3.8	8.1
3	C5	V	MET 120A	CE	IV	3.9	5.4
3	C5	V	TYR 319A	CZ	V	4.0	0.2
3	C5	V	PRO 318A	CD	IV	4.1	1.8
3	C5	V	TYR 317A	CE1	V	4.1	4.9
4	C4	V	MET 120A	CE	IV	3.4	12.8
4	C4	V	PRO 318A	CG	IV	3.6	15.5
4	C4	V	ASN 167A	OD1	II	3.7	7.0
4	C4	V	ASN 167A	CG	VI	3.7	1.6
4	C4	V	ASN 167A	CB	IV	3.8	0.9
4	C4	V	TYR 319A	OH	I	4.6	0.4
5	C3	V	ASN 167A	CG	VI	3.2	16.2
5	C3	V	ASN 167A	OD1	II	3.3	0.2
5	C3	V	MET 120A	CE	IV	3.6	3.4
5	C3	V	ASN 167A	CB	IV	3.7	1.1
6	C2	V	TYR 216A	OH	I	3.9	2.2
6	C2	V	PHE 311A	CE1	V	4.0	1.1
7	C6	V	NAP 700A	O7N	II	3.9	1.8
7	C6	V	PHE 311A	CE1	V	3.9	1.6
8	C11	V	NAP 700A	O7N	II	3.7	3.1
8	C11	V	PHE 311A	CE1	V	4.0	3.6
9	C10	V	NAP 700A	O7N	II	3.5	4.9
9	C10	V	HIS 117A	NE2	I	3.9	7.4
9	C10	V	HIS 117A	CD2	V	4.1	0.9
9	C10	V	TRP 86A	CZ3	V	4.2	7.9
9	C10	V	TRP 86A	CH2	V	4.2	2.9
9	C10	V	PHE 311A	CE1	V	4.6	2.9
10	F	VI	SER 118A	CB	VI	3.4	23.6
10	F	VI	ASN 167A	ND2	III	3.8	1.0
10	F	VI	SER 118A	OG	I	3.8	1.0
10	F	VI	MET 120A	CE	IV	4.0	4.8
10	F	VI	PHE 311A	CE1	V	4.1	3.0
10	F	VI	TRP 86A	CH2	V	4.3	5.5
11	C7	V	PHE 306A	CD1	V	3.7	12.6
11	C7	V	TYR 216A	OH	I	3.8	11.4
11	C7	V	PHE 306A	CG	V	3.8	0.4

11	C7	V	NAP	700A	O7N	II	3.9	0.2
11	C7	V	PHE	311A	CZ	V	4.1	1.1
12	C8	V	NAP	700A	C4N	V	3.5	8.5
12	C8	V	PHE	306A	CE1	V	3.7	11.0
12	C8	V	PHE	306A	CD1	V	3.9	0.2
12	C8	V	PHE	311A	CZ	V	4.6	1.1
13	C9	V	NAP	700A	O7N	II	3.6	0.2
13	C9	V	PHE	311A	CZ	V	4.9	0.7
14	C12	IV	LEU	54A	CD2	IV	3.5	10.3
15	C13	IV	LEU	54A	CD2	IV	3.8	12.6
15	C13	IV	TRP	227A	CH2	V	3.8	28.9
15	C13	IV	TRP	227A	CZ3	V	4.0	4.5
15	C13	IV	TRP	227A	CZ2	V	4.3	2.2
15	C13	IV	PHE	306A	CE1	V	4.7	0.4
15	C13	IV	TRP	227A	CE2	V	4.9	0.4
15	C13	IV	TYR	55A	CE1	V	4.9	0.9
15	C13	IV	TYR	24A	CE1	V	5.3	5.6
15	C13	IV	TYR	24A	CZ	V	5.3	0.2
15	C13	IV	PHE	311A	CZ	V	5.6	0.9
16	C14	VI	NAP	700A	C4N	V	3.2	4.0
16	C14	VI	TYR	55A	OH	I	3.4	3.4
16	C14	VI	TYR	55A	CE1	V	3.5	5.6
17	O1	II	NAP	700A	C5N	V	3.2	16.5
17	O1	II	TYR	55A	OH	I	3.7	1.9
17	O1	II	NAP	700A	C6N	V	3.7	1.6
17	O1	II	TYR	55A	CE1	V	3.7	3.1
17	O1	II	TYR	24A	CG	V	4.7	7.4
17	O1	II	PHE	306A	CZ	V	4.7	1.4
17	O1	II	TYR	24A	CD2	V	5.0	1.2
17	O1	II	TRP	227A	CZ3	V	5.4	0.2
18	O	II	TYR	55A	OH	I	2.5	23.0
18	O	II	HIS	117A	NE2	I	2.6	16.6
18	O	II	NAP	700A	C3N	V	3.0	4.5
18	O	II	TYR	55A	CE1	V	3.1	0.2

Legend:
N - ligand atom number in PDB entry
Dist - distance (A) between the ligand and protein atoms
Surf - contact surface area (A**2) between the ligand and protein atoms
* - indicates destabilizing contacts

I	Hydrophilic	- N and O that can donate and accept hydrogen bonds (e.g., oxygen of hydroxyl group of Ser. or Thr)
II	Acceptor	- N or O that can only accept a hydrogen bond
III	Donor	- N that can only donate a hydrogen bond
IV	Hydrophobic	- Cl, Br, I and all C atoms that are not in aromatic rings and do not have a covalent bond to a N or O atom
V	Aromatic	- C in aromatic rings irrespective of any other bonds formed by the atom
VI	Neutral	- C atoms that have a covalent bond to at least one atom of class I or two or more atoms from class II or III; atoms; S, F, P, and metal atoms in all cases
VII	Neutral-donor	- C atoms that have a covalent bond with only one atom of class III
VIII	Neutral-acceptor	- C atoms that have a covalent bond with only one atom of class II
