# Supplementary material for: Crystal Structures of Three Classes of Non-Steroidal Anti-Inflammatory Drugs in Complex with Aldo-Keto Reductase 1C3
Source: PLoS One. 2012 Aug 28;7(8):e43965. doi: 10.1371/journal.pone.0043965 (PMC3429426; doi:10.1371/journal.pone.0043965)
Supplement: Tables S5 — Complementarity values for (R)-ibuprofen in PDB entry 3R8G and f ull list of atomic contacts. (PDF) [file pone.0043965.s016.pdf]

Table S5. Complementarity values for (*R*)-ibuprofen in PDB entry 3R8G and full list of atomic contacts. Total number of contacts is 82.

Theoretical maximum (Å^2)	426
Actual value (Å^2)	374
Normalised complementarity	0.88

Ligand atom			Protein atom			Dist	Surf
N	Name	Class	Residue	Name	Class		
1	C1	VI	NAP 700A	C4N	V	3.4	4.3
1	C1	VI	HIS 117A	NE2	I	3.7	0.2
1	C1	VI	TYR 55A	CE1	V	3.7	8.1
1	C1	VI	LEU 54A	CD2	IV	4.2	0.4
2	O1	I	TYR 55A	OH	I	2.7	24.6
2	O1	I	HIS 117A	NE2	I	2.8	14.7
2	O1	I	TYR 55A	CE1	V	3.1	0.7
2	O1	I	NAP 700A	C3N	V	3.1	4.9
3	C2	IV	ASN 167A	OD1	II	3.4	16.8*
3	C2	IV	ASN 167A	CG	VI	3.4	2.7
3	C2	IV	TYR 216A	OH	I	3.7	1.6*
3	C2	IV	ASN 167A	CB	IV	4.1	0.4
3	C2	IV	MET 120A	CE	IV	4.3	0.9
4	O2	II	NAP 700A	C4N	V	3.7	4.5
4	O2	II	NAP 700A	C5N	V	3.8	4.7
4	O2	II	TYR 55A	CE1	V	3.9	5.7
4	O2	II	TYR 55A	CZ	V	4.4	0.2
4	O2	II	NAP 700A	C6N	V	4.5	0.3
4	O2	II	PHE 306A	CE1	V	4.5	3.3
4	O2	II	PHE 306A	CZ	V	4.6	0.7
4	O2	II	TYR 55A	CD1	V	4.8	0.2
4	O2	II	TYR 24A	CG	V	4.9	7.1
4	O2	II	TYR 24A	CD2	V	5.0	2.9
4	O2	II	TYR 24A	CD1	V	5.1	0.5
4	O2	II	TRP 227A	CZ3	V	5.2	2.1
4	O2	II	TRP 227A	CH2	V	5.2	0.2
4	O2	II	TYR 24A	CE2	V	5.3	0.2
4	O2	II	TYR 24A	CE1	V	5.3	0.5
5	C3	IV	MET 120A	CE	IV	3.7	8.1
5	C3	IV	PHE 311A	CD1	V	3.9	4.0
6	C4	IV	PRO 318A	CG	IV	3.5	32.3
6	C4	IV	MET 120A	CE	IV	3.6	12.6
6	C4	IV	PRO 318A	CD	IV	3.6	2.2
6	C4	IV	TYR 319A	CE2	V	3.9	12.1
6	C4	IV	TYR 317A	CE1	V	4.2	6.3
6	C4	IV	TYR 319A	CZ	V	4.4	1.1
6	C4	IV	ASN 167A	OD1	II	4.6	0.2*
6	C4	IV	ASN 167A	CB	IV	4.7	0.4
7	C5	IV	PHE 306A	CB	IV	3.5	24.2
7	C5	IV	PHE 311A	CD2	V	3.7	17.7
7	C5	IV	PHE 311A	CG	V	3.8	0.2
7	C5	IV	PHE 306A	O	II	4.2	6.3*
7	C5	IV	TYR 319A	CE2	V	4.2	7.2
7	C5	IV	PHE 311A	CB	IV	4.3	1.1
7	C5	IV	TYR 319A	OH	I	4.8	0.7*
7	C5	IV	TYR 216A	OH	I	4.8	0.2*
7	C5	IV	TYR 317A	CE1	V	4.9	0.7
8	C6	IV	LEU 54A	CD2	IV	3.6	9.6
8	C6	IV	HIS 117A	NE2	I	3.9	0.2*
9	C7	IV	LEU 54A	CD2	IV	3.9	11.9

9	C7	IV	TRP	227A	CZ2	V	4.1	26.5
9	C7	IV	TRP	227A	CH2	V	4.1	4.3
9	C7	IV	PHE	306A	CE1	V	4.4	3.8
9	C7	IV	TRP	227A	CE2	V	4.5	2.0
9	C7	IV	PHE	311A	CZ	V	4.6	4.7
9	C7	IV	TRP	86A	CH2	V	5.4	1.1
9	C7	IV	TYR	24A	CZ	V	6.3	0.4
10	C8	V	NAP	700A	O7N	II	3.5	0.7
10	C8	V	PHE	311A	CZ	V	4.6	0.2
11	C9	V	NAP	700A	C4N	V	3.6	9.2
11	C9	V	PHE	306A	CE1	V	3.7	12.1
11	C9	V	PHE	306A	CD1	V	3.8	0.4
11	C9	V	PHE	311A	CZ	V	4.5	0.9
12	C10	V	TYR	216A	OH	I	3.5	14.4
12	C10	V	NAP	700A	O7N	II	3.8	0.4
12	C10	V	PHE	306A	CD1	V	4.0	6.5
12	C10	V	PHE	306A	CG	V	4.1	0.4
12	C10	V	PHE	311A	CZ	V	4.5	0.4
13	C11	V	ASN	167A	ND2	III	3.6	2.9
13	C11	V	PHE	311A	CE1	V	4.3	0.4
14	C12	V	ASN	167A	ND2	III	3.7	8.7
14	C12	V	PHE	311A	CE1	V	4.1	7.4
14	C12	V	SER	118A	CB	VI	4.4	12.3
14	C12	V	SER	118A	OG	I	4.7	0.4
14	C12	V	MET	120A	CE	IV	4.7	0.9
14	C12	V	TRP	86A	CH2	V	5.0	0.7
15	C13	V	NAP	700A	O7N	II	3.6	5.2
15	C13	V	HIS	117A	NE2	I	4.0	7.0
15	C13	V	HIS	117A	CD2	V	4.1	2.7
15	C13	V	TRP	86A	CZ3	V	4.2	11.0
15	C13	V	TRP	86A	CH2	V	4.3	2.2
15	C13	V	PHE	311A	CE1	V	4.3	2.9

Legend:
N - ligand atom number in PDB entry
Dist - distance (A) between the ligand and protein atoms
Surf - contact surface area (A**2) between the ligand and protein atoms
* - indicates destabilizing contacts

I Hydrophilic - N and O that can donate and accept hydrogen bonds (e.g., oxygen of hydroxyl group of Ser. or Thr)
II Acceptor - N or O that can only accept a hydrogen bond
III Donor - N that can only donate a hydrogen bond
IV Hydrophobic - Cl, Br, I and all C atoms that are not in aromatic rings and do not have a covalent bond to a N or O atom
V Aromatic - C in aromatic rings irrespective of any other bonds formed by the atom
VI Neutral - C atoms that have a covalent bond to at least one atom of class I or two or more atoms from class II or III; atoms; S, F, P, and metal atoms in all cases
VII Neutral-donor - C atoms that have a covalent bond with only one atom of class III
VIII Neutral-acceptor - C atoms that have a covalent bond with only one atom of class II
