# Supplementary material for: Crystal Structures of Three Classes of Non-Steroidal Anti-Inflammatory Drugs in Complex with Aldo-Keto Reductase 1C3
Source: PLoS One. 2012 Aug 28;7(8):e43965. doi: 10.1371/journal.pone.0043965 (PMC3429426; doi:10.1371/journal.pone.0043965)
Supplement: Table S6 — Complementarity values for (R)-naproxen in PDB entry 3UFY and full list of atomic contacts. (PDF) [file pone.0043965.s017.pdf]

Table S6. Complementarity values for (*R*)-naproxen in PDB entry 3UFY and full list of atomic contacts. Total number of contacts is 78.

Theoretical maximum (Å^2)	434
Actual value (Å^2)	411
Normalised complementarity	0.95

Ligand atom			Protein atom				Dist	Surf
N	Name	Class	Residue		Name	Class		
1	C1	V	ASN	167A	CG	VI	3.5	4.3
1	C1	V	MET	120A	CE	IV	3.7	2.0
1	C1	V	PHE	311A	CE1	V	3.9	2.0
2	O2	II	MET	120A	CE	IV	3.2	7.4*
2	O2	II	ASN	167A	CG	VI	3.6	2.6
2	O2	II	ASN	167A	CB	IV	3.8	1.2*
3	C2	V	ASN	167A	ND2	III	3.4	8.5
3	C2	V	MET	120A	CE	IV	3.5	17.0
3	C2	V	SER	118A	OG	I	3.6	8.1
3	C2	V	ASN	167A	CG	VI	3.7	0.7
3	C2	V	PHE	311A	CE1	V	3.9	1.8
4	C3	V	ASN	167A	ND2	III	3.5	7.0
4	C3	V	SER	118A	CB	VI	3.9	11.2
4	C3	V	PHE	311A	CE1	V	3.9	5.2
4	C3	V	HIS	117A	CD2	V	4.5	2.2
4	C3	V	TRP	86A	CZ3	V	4.5	5.6
4	C3	V	TRP	86A	CH2	V	4.6	0.9
5	C4	V	NAP	700A	O7N	II	3.4	2.9
5	C4	V	PHE	311A	CZ	V	3.9	2.5
6	C5	V	PHE	311A	CZ	V	3.6	2.7
6	C5	V	NAP	700A	O7N	II	3.7	2.0
7	C6	V	TYR	216A	OH	I	3.5	9.2
7	C6	V	PHE	311A	CZ	V	3.8	6.3
7	C6	V	PHE	311A	CE2	V	4.1	1.3
7	C6	V	PHE	306A	CB	IV	4.7	2.2
8	C7	V	NAP	700A	O7N	II	3.4	5.8
8	C7	V	HIS	117A	NE2	I	3.8	6.1
8	C7	V	HIS	117A	CD2	V	4.2	0.2
8	C7	V	EDO	704A	C2	VI	4.3	9.6
8	C7	V	TRP	86A	CZ3	V	4.5	2.9
9	C8	V	NAP	700A	O7N	II	3.7	0.2
9	C8	V	EDO	704A	C2	VI	4.8	1.1
10	C9	IV	LEU	54A	CD2	IV	3.7	8.5
11	C10	IV	TRP	227A	CH2	V	3.7	29.6
11	C10	IV	LEU	54A	CD2	IV	3.9	11.9
11	C10	IV	TRP	227A	CZ3	V	4.0	3.6
11	C10	IV	TRP	227A	CZ2	V	4.1	2.7
11	C10	IV	EDO	705A	O2	I	4.5	2.5*
11	C10	IV	EDO	704A	C1	VI	4.6	1.1
11	C10	IV	EDO	705A	C2	VI	4.6	0.9
11	C10	IV	TYR	55A	CE1	V	4.9	0.9
11	C10	IV	TYR	24A	CE1	V	4.9	7.2
12	C13	V	PHE	306A	CD1	V	3.4	20.4
12	C13	V	PHE	306A	CG	V	3.7	2.7
12	C13	V	TYR	216A	OH	I	3.8	7.4
12	C13	V	NAP	700A	C4N	V	3.8	1.1
12	C13	V	PHE	311A	CZ	V	3.9	2.2
13	C11	V	NAP	700A	C4N	V	3.3	8.7
13	C11	V	PHE	306A	CE1	V	3.5	12.3

13	C11	V	PHE	306A	CD1	V	3.7	0.4
13	C11	V	PHE	311A	CZ	V	4.5	1.1
14	C12	VIII	TYR	319A	CE2	V	3.6	31.0
14	C12	VIII	PRO	318A	CG	IV	3.7	19.3
14	C12	VIII	TYR	319A	CZ	V	3.9	3.4
14	C12	VIII	TYR	319A	OH	I	3.9	2.2
14	C12	VIII	ASN	167A	OD1	II	3.9	4.3*
14	C12	VIII	MET	120A	CE	IV	4.1	4.9
14	C12	VIII	PRO	318A	CD	IV	4.3	0.9
14	C12	VIII	TYR	319A	CD2	V	4.3	0.7
14	C12	VIII	PHE	311A	CG	V	4.6	8.1
14	C12	VIII	PHE	311A	CD2	V	4.7	1.6
14	C12	VIII	TYR	317A	CE1	V	4.8	2.2
14	C12	VIII	PHE	306A	CB	IV	5.3	1.6
14	C12	VIII	PHE	306A	O	II	5.5	0.2*
15	O	I	TYR	55A	OH	I	2.5	22.3
15	O	I	HIS	117A	NE2	I	2.8	16.1
15	O	I	NAP	700A	C3N	V	3.0	5.7
15	O	I	TYR	55A	CE1	V	3.0	0.2
16	OXT	II	EDO	705A	O2	I	3.0	21.7
16	OXT	II	NAP	700A	C5N	V	3.2	4.3
16	OXT	II	TYR	55A	CE1	V	3.5	7.4
16	OXT	II	TYR	55A	OH	I	3.5	1.0
16	OXT	II	NAP	700A	C6N	V	3.5	0.5
16	OXT	II	TYR	55A	CZ	V	3.8	0.5
16	OXT	II	EDO	705A	C2	VI	3.9	0.3
16	OXT	II	TYR	24A	CG	V	4.2	2.6
17	C15	VI	NAP	700A	C4N	V	3.2	3.4
17	C15	VI	TYR	55A	CE1	V	3.4	8.1

Legend:
N - ligand atom number in PDB entry
Dist - distance (A) between the ligand and protein atoms
Surf - contact surface area (A**2) between the ligand and protein atoms
* - indicates destabilizing contacts

I	Hydrophilic	- N and O that can donate and accept hydrogen bonds (e.g., oxygen of hydroxyl group of Ser. or Thr)
II	Acceptor	- N or O that can only accept a hydrogen bond
III	Donor	- N that can only donate a hydrogen bond
IV	Hydrophobic	- Cl, Br, I and all C atoms that are not in aromatic rings and do not have a covalent bond to a N or O atom
V	Aromatic	- C in aromatic rings irrespective of any other bonds formed by the atom
VI	Neutral	- C atoms that have a covalent bond to at least one atom of class I or two or more atoms from class II or III; atoms; S, F, P, and metal atoms in all cases
VII	Neutral-donor	- C atoms that have a covalent bond with only one atom of class III
VIII	Neutral-acceptor	- C atoms that have a covalent bond with only one atom of class II
