# Supplementary material for: Crystal Structures of Three Classes of Non-Steroidal Anti-Inflammatory Drugs in Complex with Aldo-Keto Reductase 1C3
Source: PLoS One. 2012 Aug 28;7(8):e43965. doi: 10.1371/journal.pone.0043965 (PMC3429426; doi:10.1371/journal.pone.0043965)
Supplement: Table S7 — Complementarity values for Indomethacin pH 7.5 in PDB entry 3UG8 and full list of atomic contacts. (PDF) [file pone.0043965.s018.pdf]

Table S7. Complementarity values for Indomethacin pH 7.5 in PDB entry 3UG8 and full list of atomic contacts. Total number of contacts is 122.

Theoretical maximum (Å^2)	550
Actual value (Å^2)	521
Normalised complementarity	0.95

Ligand atom			Protein atom			Dist	Surf
N	Name	Class	Residue	Name	Class		
1	C	V	NAP 1001A	C4N	V	3.9	2.0
1	C	V	PHE 306A	CD1	V	4.5	0.9
1	C	V	TRP 227A	CE3	V	5.0	1.3
1	C	V	TRP 227A	CD2	V	5.4	0.7
2	C1	V	NAP 1001A	C4N	V	3.3	2.7
2	C1	V	TRP 227A	CZ3	V	4.3	2.7
3	C2	V	NAP 1001A	C5N	V	3.3	10.8
3	C2	V	TRP 227A	CZ3	V	4.1	10.3
3	C2	V	NAP 1001A	O2N	I	5.4	0.4
4	C3	V	NAP 1001A	C5N	V	3.8	4.7
4	C3	V	TYR 216A	CE2	V	4.1	0.2
4	C3	V	TRP 227A	CE3	V	4.5	4.5
5	C4	V	PHE 306A	CB	IV	3.4	14.6
5	C4	V	TYR 216A	CE2	V	4.0	8.5
5	C4	V	TYR 216A	OH	I	4.0	0.4
5	C4	V	PHE 306A	CD1	V	4.1	1.8
5	C4	V	GLU 192A	OE1	II	4.6	0.2
5	C4	V	TRP 227A	CB	IV	5.6	0.2
6	C5	V	PHE 306A	CD1	V	3.8	7.0
6	C5	V	PHE 306A	CB	IV	3.8	0.7
6	C5	V	TYR 216A	OH	I	3.9	2.0
7	C6	VIII	SER 217A	OG	I	3.3	19.5
7	C6	VIII	SER 221A	CB	VI	3.8	16.8
7	C6	VIII	PHE 306A	N	III	3.8	20.2
7	C6	VIII	TYR 305A	CB	IV	3.9	6.7
7	C6	VIII	GLU 192A	OE1	II	4.0	5.6*
7	C6	VIII	TYR 305A	C	VI	4.4	0.2
7	C6	VIII	SER 221A	OG	I	4.4	0.2
7	C6	VIII	PHE 306A	CA	VII	4.5	0.4
7	C6	VIII	TYR 216A	CE2	V	4.5	0.4
7	C6	VIII	VAL 228A	CG2	IV	4.8	6.5
7	C6	VIII	TRP 227A	CE3	V	5.7	0.2
7	C6	VIII	TRP 227A	CB	IV	6.0	0.2
8	C7	V	TRP 227A	CZ3	V	4.3	2.5
9	C8	V	NAP 1001A	O7N	II	4.0	1.3
9	C8	V	TRP 227A	CH2	V	5.0	2.0
9	C8	V	TRP 227A	CZ2	V	5.4	0.9
10	C9	VI	NAP 1001A	O7N	II	4.2	1.6
10	C9	VI	ASN 167A	ND2	III	4.8	0.7
10	C9	VI	HIS 117A	NE2	I	5.4	0.2
10	C9	VI	HIS 117A	CD2	V	5.4	0.4
10	C9	VI	SER 118A	CB	VI	5.4	0.2
11	C10	V	MET 120A	CE	IV	4.5	1.1
11	C10	V	SER 118A	CB	VI	5.4	0.2
12	C11	V	ASN 167A	ND2	III	3.5	18.6
12	C11	V	ASN 167A	CG	VI	3.6	0.2
12	C11	V	ASN 167A	OD1	II	3.6	0.7
12	C11	V	TYR 216A	OH	I	3.6	0.4
12	C11	V	ASN 167A	CB	IV	4.5	0.2
12	C11	V	MET 120A	CE	IV	4.6	1.6

12	C11	V	SER	118A	CB	VI	5.1	0.9
13	C12	V	ASN	167A	OD1	II	3.3	16.4
13	C12	V	ASN	167A	CG	VI	3.5	4.5
13	C12	V	TYR	216A	OH	I	3.6	5.8
13	C12	V	ASN	167A	CB	IV	4.2	1.8
13	C12	V	MET	120A	CE	IV	4.2	1.3
13	C12	V	TYR	319A	OH	I	4.7	1.1
13	C12	V	PHE	306A	CB	IV	4.9	0.2
14	C13	V	MET	120A	CE	IV	3.6	3.4
14	C13	V	PHE	306A	CD2	V	3.9	2.7
14	C13	V	PHE	306A	CG	V	3.9	0.9
14	C13	V	PHE	306A	CB	IV	4.5	0.2
15	C14	V	PHE	306A	CD2	V	3.4	17.9
15	C14	V	PHE	306A	CE2	V	3.4	5.6
15	C14	V	MET	120A	CE	IV	3.4	11.4
15	C14	V	PHE	306A	CZ	V	3.5	0.4
16	C15	V	PHE	306A	CE1	V	3.5	18.2
16	C15	V	PHE	306A	CD1	V	3.6	0.4
16	C15	V	PHE	306A	CZ	V	3.8	0.7
16	C15	V	MET	120A	CE	IV	3.9	5.4
16	C15	V	PHE	311A	CD1	V	4.5	6.1
17	C16	IV	LEU	54A	CD2	IV	2.8	38.6
17	C16	IV	HIS	117A	NE2	I	3.6	8.7*
17	C16	IV	TRP	86A	CZ3	V	3.9	3.1
17	C16	IV	TRP	86A	CH2	V	4.1	0.9
17	C16	IV	TRP	227A	CH2	V	5.5	1.8
17	C16	IV	TRP	227A	CZ2	V	5.7	0.9
18	C17	IV	TYR	55A	CE2	V	4.1	2.9
18	C17	IV	TRP	227A	CZ3	V	4.1	12.6
18	C17	IV	TRP	227A	CH2	V	4.2	2.9
18	C17	IV	TYR	24A	CG	V	4.6	6.7
18	C17	IV	LEU	54A	CD2	IV	4.6	1.1
18	C17	IV	TYR	24A	CD2	V	4.7	0.9
18	C17	IV	TYR	24A	CD1	V	4.8	1.3
18	C17	IV	TYR	24A	CE1	V	5.1	0.2
19	C18	VI	TYR	55A	CE2	V	3.1	11.9
19	C18	VI	TYR	55A	OH	I	3.2	0.7
19	C18	VI	NAP	1001A	C4N	V	3.3	7.0
20	N	I	NAP	1001A	O7N	II	3.9	0.2
20	N	I	TRP	227A	CE2	V	5.9	0.2
21	O	II	SER	217A	OG	I	3.4	4.9
21	O	II	NAP	1001A	C5N	V	4.0	0.3
21	O	II	SER	221A	CB	VI	4.2	2.3
21	O	II	NAP	1001A	O2N	I	4.4	1.6
21	O	II	TRP	227A	CE3	V	4.9	0.9
22	O1	II	TRP	86A	CH2	V	4.0	12.5
22	O1	II	TRP	86A	CZ3	V	4.2	1.4
22	O1	II	SER	118A	CB	VI	4.9	4.3
22	O1	II	HIS	117A	CD2	V	5.1	0.2
22	O1	II	MET	120A	CE	IV	5.3	0.2*
22	O1	II	PHE	311A	CE1	V	5.5	3.1
23	O2	II	TYR	55A	OH	I	3.1	8.7
23	O2	II	NAP	1001A	C6N	V	3.2	16.1
23	O2	II	NAP	1001A	C5N	V	3.2	0.2
23	O2	II	TYR	55A	CE2	V	3.3	3.5
23	O2	II	TYR	55A	CZ	V	3.3	1.4
23	O2	II	NAP	1001A	N1N	I	3.3	0.2
23	O2	II	TYR	24A	CB	IV	3.6	11.4*
23	O2	II	TYR	24A	CG	V	3.8	0.5
23	O2	II	NAP	1001A	C3D	VI	4.0	0.2
23	O2	II	TYR	24A	CD2	V	4.3	0.7

23	O2	II	NAP 1001A	O1N	I	4.9	0.3
24	O3	II	TYR 55A	OH	I	2.7	14.7
24	O3	II	TYR 55A	CE2	V	3.0	4.3
24	O3	II	HIS 117A	NE2	I	3.0	15.1
24	O3	II	NAP 1001A	C3N	V	3.1	4.7
25	CL	IV	TYR 319A	CE2	V	3.3	29.6
25	CL	IV	PRO 318A	CG	IV	3.7	19.6
25	CL	IV	PRO 318A	CD	IV	3.8	4.7
25	CL	IV	TYR 317A	CE1	V	3.8	9.2
25	CL	IV	TYR 319A	CZ	V	3.9	0.2
25	CL	IV	PHE 306A	CD2	V	3.9	1.2

Legend:
N - ligand atom number in PDB entry
Dist - distance (A) between the ligand and protein atoms
Surf - contact surface area (A**2) between the ligand and protein atoms
* - indicates destabilizing contacts

I	Hydrophilic	- N and O that can donate and accept hydrogen bonds (e.g., oxygen of hydroxyl group of Ser. or Thr)
II	Acceptor	- N or O that can only accept a hydrogen bond
III	Donor	- N that can only donate a hydrogen bond
IV	Hydrophobic	- Cl, Br, I and all C atoms that are not in aromatic rings and do not have a covalent bond to a N or O atom
V	Aromatic	- C in aromatic rings irrespective of any other bonds formed by the atom
VI	Neutral	- C atoms that have a covalent bond to at least one atom of class I or two or more atoms from class II or III; atoms; S, F, P, and metal atoms in all cases
VII	Neutral-donor	- C atoms that have a covalent bond with only one atom of class III
VIII	Neutral-acceptor	- C atoms that have a covalent bond with only one atom of class II
