# Supplementary material for: Crystal Structures of Three Classes of Non-Steroidal Anti-Inflammatory Drugs in Complex with Aldo-Keto Reductase 1C3
Source: PLoS One. 2012 Aug 28;7(8):e43965. doi: 10.1371/journal.pone.0043965 (PMC3429426; doi:10.1371/journal.pone.0043965)
Supplement: Table S8 — PROPKA Calculations. (PDF) [file pone.0043965.s019.pdf]

**Table S8. PROPKA Calculations.**

| Residue/Atom | Theoretical p$K_a$ | 1S1P (minus acetate) p$K_a$ | 1S2A (minus UNK atom) p$K_a$ | Indomethacin pH 7.5 p$K_a$ |
|---|---|---|---|---|
| H117 NE2 | 6.5 | 1.6 | 1.5 | 1.7 |
| Y55 OH | 10.0 | 16.1 | 16.8 | 20.4 |
| NADP 01N/O2N | 6.0 | 6.9 | 11.8 | 6.7 |
| IMN $CO_2^-$/$CO_2H$ | 4.5 | n/a | 6.6 | 9.3 |
