# Supplementary material for: Crystal Structures of Three Classes of Non-Steroidal Anti-Inflammatory Drugs in Complex with Aldo-Keto Reductase 1C3
Source: PLoS One. 2012 Aug 28;7(8):e43965. doi: 10.1371/journal.pone.0043965 (PMC3429426; doi:10.1371/journal.pone.0043965)
Supplement: Table S9 — Complementarity values for zomepirac in PDB entry 3R8H and full list of atomic contacts. (PDF) [file pone.0043965.s020.pdf]

Table S9. Complementarity values for zomepirac in PDB entry 3R8H and full list of atomic contacts. Total number of contacts is 92.

Theoretical maximum (Å^2)	484
Actual value (Å^2)	436
Normalised complementarity	0.90

Ligand atom			Protein atom					
N	Name	Class	Residue		Name	Class	Dist	Surf
1	CL	IV	TYR	319A	CE2	V	3.3	28.2
1	CL	IV	TYR	317A	CE1	V	3.6	20.2
1	CL	IV	PRO	318A	CG	IV	3.6	14.1
1	CL	IV	PHE	306A	CD1	V	3.8	1.6
1	CL	IV	PRO	318A	CD	IV	3.8	1.8
2	N1	I	NAP	700A	O7N	II	3.8	0.2
3	C2	V	TRP	227A	CZ2	V	4.3	3.1
3	C2	V	TRP	227A	CH2	V	4.7	0.9
4	C3	V	NAP	700A	C4N	V	3.4	11.2
4	C3	V	NAP	700A	C5N	V	3.5	3.8
4	C3	V	TRP	227A	CZ2	V	3.9	15.0
4	C3	V	TRP	227A	NE1	III	4.2	1.1
5	C4	V	NAP	700A	C4N	V	3.8	2.0
5	C4	V	TRP	227A	NE1	III	4.3	1.3
5	C4	V	TRP	227A	CE2	V	4.5	1.6
5	C4	V	PHE	306A	CD2	V	4.8	0.2
6	C5	V	NAP	700A	O7N	II	3.9	2.2
6	C5	V	TRP	227A	CD2	V	5.9	0.2
7	C6	VIII	EDO	334A	O2	I	3.5	0.4
7	C6	VIII	NAP	700A	O7N	II	4.2	1.6*
7	C6	VIII	ASN	167A	ND2	III	4.8	0.7
7	C6	VIII	HIS	117A	CD2	V	5.5	0.4
8	O6	II	EDO	334A	O2	I	2.8	15.8
8	O6	II	EDO	334A	C2	VI	3.4	4.7
8	O6	II	TRP	86A	CH2	V	3.8	2.6
8	O6	II	TRP	86A	CZ3	V	4.2	0.2
8	O6	II	PHE	311A	CE2	V	4.8	1.6
8	O6	II	SER	118A	CB	VI	5.0	0.2
9	C7	IV	TYR	55A	CE1	V	4.0	4.7
9	C7	IV	TYR	24A	CG	V	4.3	9.4
9	C7	IV	TYR	24A	CD2	V	4.4	4.0
9	C7	IV	LEU	54A	CD2	IV	4.4	4.5
9	C7	IV	TRP	227A	CZ2	V	4.6	5.2
9	C7	IV	TYR	24A	CD1	V	4.6	1.3
9	C7	IV	TYR	24A	CE2	V	4.6	1.3
9	C7	IV	TYR	24A	CZ	V	4.8	0.9
9	C7	IV	TRP	227A	CH2	V	4.8	0.9
10	C8	VI	TYR	55A	OH	I	3.2	5.4
10	C8	VI	TYR	55A	CE1	V	3.2	5.2
10	C8	VI	NAP	700A	C4N	V	3.3	7.2
11	C9	VI	LEU	54A	CD2	IV	3.2	27.4
11	C9	VI	HIS	117A	NE2	I	3.4	12.3
11	C9	VI	TRP	86A	CZ3	V	3.8	6.3
11	C9	VI	TRP	86A	CH2	V	3.9	0.9
12	OH	II	NAP	700A	C6N	V	3.1	17.2
12	OH	II	TYR	55A	OH	I	3.2	5.2
12	OH	II	TYR	24A	CB	IV	3.4	18.0*
12	OH	II	TYR	24A	CG	V	3.6	0.2
12	OH	II	NAP	700A	C3D	VI	3.9	0.2
12	OH	II	TYR	24A	CD2	V	4.0	1.0

12	OH	II	NAP	700A	O1N	I	4.8	0.3
13	C10	IV	PHE	306A	CB	IV	3.7	25.8
13	C10	IV	PHE	306A	CD2	V	3.8	6.1
13	C10	IV	TRP	227A	NE1	III	4.0	7.2*
13	C10	IV	TYR	216A	OH	I	4.1	7.0*
13	C10	IV	NAP	700A	C4N	V	4.4	0.9
13	C10	IV	TYR	216A	CE1	V	4.6	3.6
13	C10	IV	PHE	306A	N	III	5.3	0.2*
13	C10	IV	GLU	192A	OE1	II	5.4	0.2*
14	C1B	V	EDO	334A	O2	I	3.4	2.0
15	C2B	V	EDO	334A	O2	I	3.3	7.9
15	C2B	V	PHE	306A	CE2	V	3.8	15.5
15	C2B	V	PHE	306A	CD2	V	3.8	0.2
15	C2B	V	EDO	334A	C2	VI	3.8	4.7
15	C2B	V	PHE	311A	CD2	V	4.0	2.7
15	C2B	V	PHE	311A	CE2	V	4.5	0.4
16	C3B	V	PHE	306A	CD1	V	3.6	15.3
16	C3B	V	MET	120A	CE	IV	3.6	9.9
16	C3B	V	PHE	306A	CE1	V	3.6	4.3
16	C3B	V	PHE	306A	CZ	V	3.7	0.7
16	C3B	V	PHE	311A	CD2	V	4.3	2.0
16	C3B	V	PHE	311A	CB	IV	4.4	0.7
17	C4B	V	MET	120A	CE	IV	3.6	3.8
17	C4B	V	PHE	306A	CD1	V	3.9	3.8
18	C5B	V	ASN	167A	OD1	II	3.3	16.2
18	C5B	V	TYR	216A	OH	I	3.4	5.8
18	C5B	V	ASN	167A	CG	VI	3.6	3.8
18	C5B	V	ASN	167A	CB	IV	4.3	1.6
18	C5B	V	MET	120A	CE	IV	4.3	0.4
18	C5B	V	TYR	319A	OH	I	4.3	4.5
18	C5B	V	PHE	306A	CG	V	4.7	0.7
18	C5B	V	PHE	306A	CB	IV	4.8	0.7
18	C5B	V	TYR	319A	CZ	V	4.8	0.2
19	C6B	V	TYR	216A	OH	I	3.4	12.8
19	C6B	V	ASN	167A	OD1	II	3.5	4.7
19	C6B	V	ASN	167A	CG	VI	3.6	6.7
19	C6B	V	ASN	167A	ND2	III	3.6	1.1
19	C6B	V	EDO	334A	O2	I	4.2	0.9
20	OXT	I	TYR	55A	OH	I	2.5	17.7
20	OXT	I	HIS	117A	NE2	I	3.0	20.1
20	OXT	I	TYR	55A	CE1	V	3.0	0.5
20	OXT	I	NAP	700A	C3N	V	3.0	5.2

Legend:
N - ligand atom number in PDB entry
Dist - distance (A) between the ligand and protein atoms
Surf - contact surface area (A**2) between the ligand and protein atoms
* - indicates destabilizing contacts

I	Hydrophilic	- N and O that can donate and accept hydrogen bonds (e.g., oxygen of hydroxyl group of Ser. or Thr)
II	Acceptor	- N or O that can only accept a hydrogen bond
III	Donor	- N that can only donate a hydrogen bond
IV	Hydrophobic	- Cl, Br, I and all C atoms that are not in aromatic rings and do not have a covalent bond to a N or O atom
V	Aromatic	- C in aromatic rings irrespective of any other bonds formed by the atom
VI	Neutral	- C atoms that have a covalent bond to at least one atom of class I or two or more atoms from class II

or III; atoms; S, F, P, and metal atoms in all cases

VII	Neutral-donor	- C atoms that have a covalent bond with only one atom of class III
VIII	Neutral-acceptor	- C atoms that have a covalent bond with only one atom of class II
