# Supplementary material for: Crystal Structures of Three Classes of Non-Steroidal Anti-Inflammatory Drugs in Complex with Aldo-Keto Reductase 1C3
Source: PLoS One. 2012 Aug 28;7(8):e43965. doi: 10.1371/journal.pone.0043965 (PMC3429426; doi:10.1371/journal.pone.0043965)
Supplement: Table S10 — Complementarity values for sulindac in PDB entry 3R7M and full list of atomic contacts. (PDF) [file pone.0043965.s021.pdf]

Table S10. Complementarity values for sulindac in PDB entry 3R7M and full list of atomic contacts. Total number of contacts is 104.

Theoretical maximum (Å^2)	581
Actual value (Å^2)	433
Normalised complementarity	0.75

Ligand atom			Protein atom				
N	Name	Class	Residue	Name	Class	Dist	Surf
1	C20	V	TRP 86A	CZ2	V	3.8	14.6
1	C20	V	LEU 54A	CD1	IV	4.6	0.7
1	C20	V	LEU 122A	CD1	IV	5.4	0.2
2	C19	V	LEU 54A	CD1	IV	3.8	19.7
2	C19	V	TRP 86A	CH2	V	3.9	3.1
2	C19	V	TRP 227A	CZ2	V	4.8	0.4
2	C19	V	ARG 226A	NH1	III	6.0	0.4
3	C5	V	TRP 227A	CZ2	V	3.8	1.6
4	C6	IV	TRP 227A	CZ2	V	3.4	10.1
4	C6	IV	LEU 54A	CD2	IV	3.8	3.4
4	C6	IV	LEU 54A	CD1	IV	4.5	0.4
4	C6	IV	ARG 226A	NH1	III	5.3	1.1*
4	C6	IV	TYR 24A	OH	I	5.6	0.2*
5	C7	V	LEU 54A	CD2	IV	3.4	1.6
5	C7	V	TRP 227A	CZ2	V	4.2	0.7
5	C7	V	TRP 227A	CE2	V	4.6	0.7
5	C7	V	PHE 311A	CZ	V	4.8	0.4
6	C18	V	LEU 54A	CD2	IV	3.6	3.1
6	C18	V	PHE 311A	CZ	V	4.3	2.0
7	C17	V	PHE 311A	CE1	V	3.6	4.9
7	C17	V	TRP 86A	CH2	V	3.6	11.2
8	C16	V	PHE 311A	CE1	V	3.7	12.8
8	C16	V	TRP 86A	CZ3	V	3.8	11.9
8	C16	V	TRP 86A	CH2	V	3.9	1.3
8	C16	V	PHE 311A	CD1	V	4.6	0.7
8	C16	V	SER 118A	CB	VI	4.7	4.9
8	C16	V	EDO 333A	C2	VI	4.7	0.2
8	C16	V	SER 118A	OG	I	4.9	0.7
8	C16	V	MET 120A	CE	IV	5.2	0.9
9	C15	V	HIS 117A	NE2	I	3.9	3.6
9	C15	V	HIS 117A	CD2	V	3.9	0.2
9	C15	V	EDO 333A	C2	VI	4.1	3.6
10	F	VI	ASN 167A	ND2	III	2.8	30.5
10	F	VI	NAP 700A	O7N	II	3.2	1.2
10	F	VI	EDO 333A	C2	VI	3.6	7.3
10	F	VI	HIS 117A	CD2	V	3.7	6.1
10	F	VI	SER 118A	CB	VI	3.7	10.1
10	F	VI	MET 120A	CE	IV	5.1	0.2
11	C14	V	NAP 700A	O7N	II	3.1	16.2
11	C14	V	HIS 117A	NE2	I	3.5	3.1
11	C14	V	NAP 700A	C4N	V	4.4	1.1
11	C14	V	EDO 333A	C2	VI	4.5	5.2
11	C14	V	EDO 333A	O2	I	4.8	0.2
11	C14	V	PHE 306A	CE1	V	5.1	0.9
12	C13	V	LEU 54A	CD2	IV	3.9	1.6
12	C13	V	PHE 306A	CE1	V	4.6	2.7
12	C13	V	PHE 311A	CZ	V	4.9	0.2
13	C10	V	LEU 54A	CD2	IV	3.9	0.7
13	C10	V	PHE 306A	CE1	V	4.2	2.0
14	C11	IV	NAP 700A	C4N	V	3.4	11.0

14	C11	IV	NAP	700A	C5N	V	3.6	3.1
14	C11	IV	PHE	306A	CZ	V	3.9	9.4
14	C11	IV	TYR	24A	CD2	V	5.3	0.4
15	C12	VI	TYR	55A	OH	I	3.1	4.5
15	C12	VI	NAP	700A	C4N	V	3.2	8.7
15	C12	VI	TYR	55A	CE1	V	3.3	6.7
16	O3	II	TYR	55A	OH	I	2.5	17.8
16	O3	II	HIS	117A	NE2	I	3.0	20.1
16	O3	II	TYR	55A	CE1	V	3.0	2.3
16	O3	II	NAP	700A	C3N	V	3.1	3.1
17	O2	II	TYR	55A	OH	I	3.1	7.6
17	O2	II	NAP	700A	C6N	V	3.2	14.7
17	O2	II	NAP	700A	N1N	I	3.3	0.2
17	O2	II	TYR	55A	CZ	V	3.4	4.7
17	O2	II	TYR	55A	CE1	V	3.4	0.9
17	O2	II	NAP	700A	C2D	VI	3.6	4.0
17	O2	II	TYR	24A	CB	IV	3.7	9.4*
17	O2	II	NAP	700A	C3D	VI	3.9	0.2
17	O2	II	TYR	24A	CG	V	3.9	0.7
17	O2	II	TYR	24A	CD2	V	4.3	0.7
17	O2	II	NAP	700A	O1N	I	5.0	0.2
18	C8	V	LEU	54A	CD2	IV	3.6	3.1
18	C8	V	TRP	227A	CH2	V	4.0	0.9
18	C8	V	TRP	227A	CZ3	V	4.2	1.6
18	C8	V	PHE	306A	CE1	V	4.5	0.7
19	C9	IV	TRP	227A	CH2	V	3.2	21.3
19	C9	IV	TRP	227A	CZ3	V	3.4	4.9
19	C9	IV	TYR	24A	CZ	V	3.6	25.8
19	C9	IV	TYR	24A	OH	I	3.8	0.7*
19	C9	IV	TYR	24A	CE2	V	3.8	2.2
19	C9	IV	TYR	24A	CE1	V	4.0	3.4
19	C9	IV	LEU	54A	CD2	IV	4.3	1.6
19	C9	IV	TYR	24A	CD2	V	4.4	0.2
19	C9	IV	PHE	306A	CZ	V	5.3	0.2
20	C4	V	TRP	227A	NE1	III	3.6	16.8
20	C4	V	PHE	311A	CZ	V	3.7	4.0
20	C4	V	TRP	227A	CZ2	V	3.8	0.4
21	C3	V	PHE	311A	CE1	V	3.8	13.5
21	C3	V	PHE	311A	CZ	V	4.1	1.1
21	C3	V	TRP	227A	NE1	III	4.1	4.9
21	C3	V	SER	310A	CB	VI	4.4	4.0
22	C2	V	TRP	86A	CZ2	V	4.5	1.8
22	C2	V	TRP	86A	CH2	V	4.5	0.7
23	S	VI	LEU	122A	CD1	IV	4.6	9.9
24	O1	I	SER	310A	CB	VI	4.2	6.9
24	O1	I	SER	310A	O	II	4.2	6.4
24	O1	I	LEU	122A	CD1	IV	4.3	9.2*
24	O1	I	LEU	122A	CD2	IV	4.5	5.9*
24	O1	I	PHE	311A	CE1	V	5.0	1.6
24	O1	I	PHE	311A	CD1	V	5.2	0.3
24	O1	I	TRP	86A	CH2	V	5.4	0.3
24	O1	I	PHE	139A	CE1	V	5.5	2.4
25	C1	IV	SER	310A	OG	I	4.3	13.7*
25	C1	IV	SER	310A	CB	VI	4.3	1.6

Legend:

N - ligand atom number in PDB entry

Dist - distance (A) between the ligand and protein atoms

Surf - contact surface area (A2) between the ligand and protein atoms**

* - indicates destabilizing contacts

I	Hydrophilic	- N and O that can donate and accept hydrogen bonds (e.g., oxygen of hydroxyl group of Ser. or Thr)
II	Acceptor	- N or O that can only accept a hydrogen bond
III	Donor	- N that can only donate a hydrogen bond
IV	Hydrophobic	- Cl, Br, I and all C atoms that are not in aromatic rings and do not have a covalent bond to a N or O atom
V	Aromatic	- C in aromatic rings irrespective of any other bonds formed by the atom
VI	Neutral	- C atoms that have a covalent bond to at least one atom of class I or two or more atoms from class II or III; atoms; S, F, P, and metal atoms in all cases
VII	Neutral-donor	- C atoms that have a covalent bond with only one atom of class III
VIII	Neutral-acceptor	- C atoms that have a covalent bond with only one atom of class II
