# Supplementary material for: Crystal Structures of Three Classes of Non-Steroidal Anti-Inflammatory Drugs in Complex with Aldo-Keto Reductase 1C3
Source: PLoS One. 2012 Aug 28;7(8):e43965. doi: 10.1371/journal.pone.0043965 (PMC3429426; doi:10.1371/journal.pone.0043965)
Supplement: Table S11 — Complementarity values for (S)-naproxen in PDB entry 3R58 and full list of atomic contacts. (PDF) [file pone.0043965.s022.pdf]

Table S11. Complementarity values for (*S*)-naproxen in PDB entry 3R58 and full list of atomic contacts. Total number of contacts is 86.

Theoretical maximum (Å^2)	429
Actual value (Å^2)	384
Normalised complementarity	0.90

Ligand atom			Protein atom				Dist	Surf
N	Name	Class	Residue		Name	Class		
1	C1	V	ASN	167A	CG	VI	3.7	3.6
1	C1	V	MET	120A	CE	IV	3.8	3.6
1	C1	V	PHE	311A	CE1	V	4.2	2.0
2	O2	II	MET	120A	CE	IV	3.1	14.6*
2	O2	II	ASN	167A	CG	VI	3.9	1.6
2	O2	II	ASN	167A	CB	IV	4.1	0.9*
3	C2	V	TYR	216A	OH	I	3.6	4.9
3	C2	V	PHE	311A	CE2	V	4.1	8.1
3	C2	V	PHE	306A	CB	IV	4.3	4.3
4	C14	IV	NAP	700A	C4N	V	3.7	0.7
4	C14	IV	NAP	700A	C5N	V	4.0	0.9
4	C14	IV	PHE	306A	CE1	V	4.9	4.9
4	C14	IV	TRP	227A	CH2	V	5.0	1.1
4	C14	IV	TRP	227A	CZ3	V	5.1	0.7
4	C14	IV	PHE	306A	CZ	V	5.3	0.2
4	C14	IV	EDO	334A	O2	I	5.4	1.6*
5	C3	V	TYR	216A	OH	I	3.4	11.4
5	C3	V	PHE	306A	CG	V	3.9	16.2
5	C3	V	PHE	306A	CB	IV	4.0	1.1
5	C3	V	PHE	311A	CZ	V	4.0	2.9
6	C4	V	NAP	700A	O7N	II	3.5	2.5
6	C4	V	PHE	311A	CZ	V	4.0	2.7
7	C5	V	NAP	700A	O7N	II	3.4	2.9
7	C5	V	PHE	311A	CZ	V	4.2	2.5
8	C6	V	ASN	167A	ND2	III	3.4	10.1
8	C6	V	MET	120A	CE	IV	4.0	11.4
8	C6	V	PHE	311A	CE1	V	4.1	3.8
8	C6	V	SER	118A	OG	I	4.2	7.0
8	C6	V	TRP	86A	CH2	V	5.5	0.7
9	C7	V	NAP	700A	C4N	V	3.4	12.1
9	C7	V	PHE	306A	CE1	V	4.1	12.3
9	C7	V	PHE	306A	CD1	V	4.1	0.9
9	C7	V	PHE	311A	CZ	V	4.6	2.7
9	C7	V	TRP	227A	CE3	V	5.8	0.9
10	C8	V	NAP	700A	O7N	II	3.5	0.2
10	C8	V	PHE	311A	CZ	V	5.2	0.4
10	C8	V	TRP	227A	CH2	V	5.7	0.4
10	C8	V	TRP	227A	CZ2	V	5.8	0.7
11	C10	IV	LEU	54A	CD2	IV	3.7	21.1
11	C10	IV	TYR	55A	CE1	V	4.2	4.9
11	C10	IV	TRP	227A	CH2	V	4.2	22.7
11	C10	IV	TYR	24A	CE1	V	4.5	11.9
11	C10	IV	TRP	227A	CZ3	V	4.6	0.7
11	C10	IV	TRP	227A	CZ2	V	4.7	1.3
11	C10	IV	TYR	24A	CZ	V	4.7	0.4
12	C13	V	NAP	700A	O7N	II	3.3	5.4
12	C13	V	HIS	117A	NE2	I	3.8	5.8
12	C13	V	HIS	117A	CD2	V	3.9	4.3
12	C13	V	TRP	86A	CZ3	V	4.0	12.8
12	C13	V	TRP	86A	CH2	V	4.3	2.2

12	C13	V	PHE	311A	CE1	V	4.8	2.2
13	C11	V	NAP	700A	O7N	II	3.3	1.6
13	C11	V	HIS	117A	NE2	I	3.5	5.6
13	C11	V	HIS	117A	CE1	V	4.3	0.9
13	C11	V	LEU	54A	CD2	IV	4.3	5.4
13	C11	V	TRP	86A	CH2	V	4.7	0.2
13	C11	V	PHE	311A	CZ	V	5.3	0.7
14	C12	VIII	TYR	319A	CE2	V	3.7	28.9
14	C12	VIII	ASN	167A	OD1	II	3.7	10.8*
14	C12	VIII	TYR	319A	OH	I	3.9	1.6
14	C12	VIII	TYR	319A	CZ	V	4.0	1.3
14	C12	VIII	PRO	318A	CG	IV	4.0	16.8
14	C12	VIII	MET	120A	CE	IV	4.2	2.5
14	C12	VIII	PRO	318A	CD	IV	4.3	1.6
14	C12	VIII	TYR	319A	CD2	V	4.4	0.2
14	C12	VIII	PHE	311A	CG	V	4.9	5.4
14	C12	VIII	PHE	311A	CD2	V	4.9	0.9
14	C12	VIII	TYR	317A	CE1	V	5.0	2.5
15	O	II	NAP	700A	C5N	V	2.9	16.1
15	O	II	NAP	700A	C6N	V	3.1	6.1
15	O	II	TYR	55A	OH	I	3.2	4.3
15	O	II	TYR	55A	CE1	V	3.5	3.1
15	O	II	TYR	55A	CZ	V	3.6	0.7
15	O	II	NAP	700A	C2D	VI	4.1	1.6
15	O	II	TYR	24A	CG	V	4.2	7.4
15	O	II	TYR	24A	CB	IV	4.2	0.2*
15	O	II	TYR	24A	CD2	V	4.6	1.0
15	O	II	EDO	334A	C2	VI	4.9	0.9
16	OXT	II	TYR	55A	OH	I	2.6	17.3
16	OXT	II	HIS	117A	NE2	I	2.9	21.8
16	OXT	II	NAP	700A	C3N	V	3.0	3.3
16	OXT	II	TYR	55A	CE1	V	3.1	1.4
17	C15	VI	NAP	700A	C4N	V	3.1	8.1
17	C15	VI	NAP	700A	C3N	V	3.3	0.4
17	C15	VI	TYR	55A	OH	I	3.3	3.4
17	C15	VI	TYR	55A	CE1	V	3.4	1.6

Legend:
N - ligand atom number in PDB entry
Dist - distance (A) between the ligand and protein atoms
Surf - contact surface area (A**2) between the ligand and protein atoms
* - indicates destabilizing contacts

I	Hydrophilic	- N and O that can donate and accept hydrogen bonds (e.g., oxygen of hydroxyl group of Ser. or Thr)
II	Acceptor	- N or O that can only accept a hydrogen bond
III	Donor	- N that can only donate a hydrogen bond
IV	Hydrophobic	- Cl, Br, I and all C atoms that are not in aromatic rings and do not have a covalent bond to a N or O atom
V	Aromatic	- C in aromatic rings irrespective of any other bonds formed by the atom
VI	Neutral	- C atoms that have a covalent bond to at least one atom of class I or two or more atoms from class II or III; atoms; S, F, P, and metal atoms in all cases
VII	Neutral-donor	- C atoms that have a covalent bond with only one atom of class III
VIII	Neutral-acceptor	- C atoms that have a covalent bond with only one atom of class II
