# Supplementary material for: Crystal Structures of Three Classes of Non-Steroidal Anti-Inflammatory Drugs in Complex with Aldo-Keto Reductase 1C3
Source: PLoS One. 2012 Aug 28;7(8):e43965. doi: 10.1371/journal.pone.0043965 (PMC3429426; doi:10.1371/journal.pone.0043965)
Supplement: Table S12 — Comparison of predicted and actual binding poses for NSAIDS docked into Indomethacin or flufenamic acid bound AKR1C3. (PDF) [file pone.0043965.s023.pdf]

Table S12. Comparison of predicted and actual binding poses for NSAIDS docked into Indomethacin or flufenamic acid bound AKR1C3.

	Rank order and RMSD of predicted poses compared to native binding modes (Å)					
	Indomethacin (1S2A)			Flufenamic acid (1S2C)		
Compound	GA10 SE2	DS2	DS3	GA10 SE2	DS2	DS3
Indomethacin	Rank 1 (0.67)*	Rank 2 (0.44)*	Rank 1 (4.16)*<br>Rank 10 (1.51)	-	Rank 2 (3.96)	Rank 2 (4.02)
Flufenamic acid	Rank 3 (2.62)	Rank 1 (2.62)	Rank 1 (2.79)	Rank 7 (2.37)	Rank 5 (1.36)	Rank 3 (1.21)
Meclofenamic acid	Rank 2 (3.05)	Rank 1 (3.11)<br>Rank 9 (2.25)	Rank 4 (3.26)	Rank 1 (2.44)<br>Rank 2 (0.74)	Rank 1 (2.39)<br>Rank 5 (0.59)	Rank 1 (4.20)<br>Rank 3 (0.78)
Mefenamic acid	Rank 1 (2.85)	Rank 1 (2.69)<br>Rank 5 (2.21)	Rank 1 (2.83)	Rank 2 (0.66)	Rank 4 (0.47)	Rank 1 (0.64)
(*R*)-Flurbiprofen	Rank 7 (2.36)	Rank 1 (2.33)	Rank 2 (2.11)	Rank 1 (0.73)	Rank 1 (1.60)<br>Rank 2 (1.26)	Rank 1 (0.76)
(*R*)-Ibuprofen	Rank 1 (5.22)<br>Rank 6 (2.98)	Rank 1 (5.22)<br>Rank 4 (2.98)	-	Rank 1 (0.62)	Rank 1 (0.62)	Rank 1 (0.65)
(*R*)-Naproxen	Rank 1 (0.96)	Rank 1 (1.99)	Rank 1 (1.92)	Rank 1 (2.27)	Rank 1 (1.99)<br>Rank 3 (0.98)	Rank 1(2.20)<br>Rank 3 (1.91)
(*S*)-Naproxen	Rank 8 (2.39)	Rank 1 (2.39)<br>Rank 3 (0.61)	Rank 5 (0.75)	Rank 1 (1.00)<br>Rank 5 (0.63)	Rank 1 (1.05)	Rank 1 (0.97)

Zomepirac	-	-	Rank 5 (2.12)	-	Rank 1 (3.13)<br>Rank 6 (2.09)	Rank 2 (3.26)<br>Rank 3 (2.22)
Sulindac	-	-	-	Rank 1 (6.17)	Rank 1 (6.43)	Rank 1 (6.19)<br>Rank 7 (3.69)

* indicates poses that adopted the binding mode for indomethacin observed at pH6.0

All poses were inspected visually and only those that had agreement with the actual binding mode were further examined. The primary criterion was good agreement in the positions of the carboxylic acid and aromatic centres. Where this was not possible poses clearly similar to the actual binding mode were also used.
